# Lithium‐Aluminate‐Catalyzed Hydrophosphination Applications

**DOI:** 10.1002/anie.201906807

**Published:** 2019-07-25

**Authors:** Victoria A. Pollard, Allan Young, Ross McLellan, Alan R. Kennedy, Tell Tuttle, Robert E. Mulvey

**Affiliations:** ^1^ WestCHEM Department of Pure and Applied Chemistry University of Strathclyde Glasgow G1 1XL UK

**Keywords:** aluminate, homogeneous catalysis, hydrophosphination, lithium, phosphines

## Abstract

Synthesized, isolated, and characterized by X‐ray crystallography and NMR spectroscopic studies, lithium phosphidoaluminate iBu_3_AlPPh_2_Li(THF)_3_ has been tested as a catalyst for hydrophosphination of alkynes, alkenes, and carbodiimides. Based on the collective evidence of stoichiometric reactions, NMR monitoring studies, kinetic analysis, and DFT calculations, a mechanism involving deprotonation, alkyne insertion, and protonolysis is proposed for the [iBu_3_AlHLi]_2_ aluminate catalyzed hydrophosphination of alkynes with diphenylphosphine. This study enhances further the development of transition‐metal‐free, atom‐economical homogeneous catalysis using common sustainable main‐group metals.

## Introduction

Phosphines are utilized in a range of applications spanning agriculture, medicinal chemistry, and organocatalysis, while their ubiquity as ligands in transition metal catalysis is legend.^1^ Hydrophosphination, adding a P−H bond across an unsaturated C−E (E=C,N) bond, offers atom economy to preparing phosphines.^2^ Many transition‐metal based (for example, on Fe, Ni, Pd, and Zr)2b, 2c, 3 and rare‐earth‐metal based(for example, on La, Sm, and Yb)^3^ catalysts have been used for hydrophosphination, while solvent and catalyst‐free hydrophosphinations can also be thermally induced under certain circumstances.^4^ Developing catalysts based on main‐group metals is currently trending in homogeneous catalysis including hydrophosphination.^5^ K, Ca, and Mg complexes have been reported as hydrophosphination catalysts for alkene, alkyne, and carbodiimide substrates with diphenyl phosphine (HPPh_2_), forming alkyl phosphines, vinyl phosphines, and phosphaguanidines, respectively.3a, 3b, 6 Tin complexes are also capable of catalyzing hydrophosphination reactions,2c, 7 though Cp*_2_SnCl_2_ (Cp*=pentamethylcyclopentadienyl) required an H_2_ atmosphere to inhibit competing phosphine dehydrocoupling of HPPh_2_.7b, 8 Attractive industrially owing to its high natural abundance and low toxicity, aluminum is gaining prominence in this main group catalysis enlightenment.^9^ Work by Roesky, Wright, Cowley/Thomas, Harder, Stephan, and others, have successfully employed Al‐based catalysts in hydroboration and hydrogenation.^10^ Uhl has also demonstrated that a P/Al geminal FLP can stoichiometrically hydrophosphinate heteroatom substituted nitriles, generating imines incorporated into 5‐atom AlCPCN heterocycles.^11^ Examples also exist of Al‐catalyzed hydrophosphonylation (using P^V^ reagents).^12^ However, to our knowledge no examples exist of Al‐catalyzed hydrophosphination (using P^III^ reagents) of alkynes, alkenes, or carbodiimides.

Bimetallic ate complexes, which can synergistically enhance stoichiometric reactivities over their neutral monometallic components, are a core theme of our research.^13^ Recently expanding this work into the catalytic regime, we used lithium aluminates as catalysts for hydroboration of aldehydes, ketones, imines and acetylenes.^14^ In general, the charged bimetallic species proved more active catalysts than their neutral monometallic components.14b Herein, we probe the ability of our most active lithium aluminate, [iBu_3_AlHLi]_2_, **1**, as a catalyst for hydrophosphination of alkynes, alkenes, and carbodiimides.

## Results and Discussion

**Figure 1:**
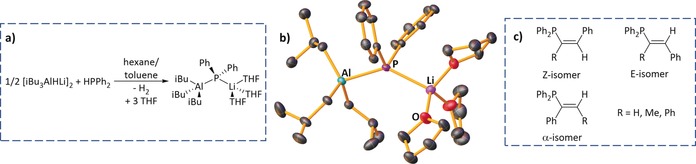
**a)** Synthesis of iBu_3_AlPPh_2_Li(THF)_3_, **2**; **b)** Molecular structure of **2**, H atoms and disordered THF molecules omitted for clarity, thermal ellipsoids drawn at 40 % probability; **c)** Depiction of *E*‐, *Z*‐stereoisomers and α‐regioisomers arising from hydrophosphination of alkyne substrates.

First, we reacted phenylacetylene with HPPh_2_ and 10 mol % of dimeric [iBu_3_AlHLi]_2_ in C_6_D_6_ at 70 °C. This reaction gave a very low 5 % conversion after 20 h and it was proposed a higher boiling point solvent would be required to allow the catalysis to proceed. Moving to [D_8_]toluene at 110 °C yielded 72 % conversion (1:8 *E*/*Z* ratio; Figure 1) within 20 h, to the anti‐Markovnikov product consistent with *anti* addition of H−P across the C≡C bond. Changing the solvent to polar [D_8_]THF (65 °C) lowered the *E*/*Z* selectivity to 1:3. We propose that in [D_8_]THF solution the resulting lithium aluminum phosphide (see below) exists in an equilibrium, as observed by the presence of three separate distinct signals for the *iso*‐butyl ligands in the ^1^H NMR spectrum. Using CD_2_Cl_2_ (40 °C) poisoned the catalyst giving no product. Satisfied this system is a capable hydrophosphination catalyst, a range of alkynes were screened (Table 1). Internal alkynes, diphenylacetylene, and 1‐phenyl‐1‐propyne reacted faster than terminal alkynes. However, more challenging unactivated alkynes, 1‐hexyne, and 3‐hexyne did not react, in common with other reports of main‐group‐catalyzed hydrophosphination.7b

**Table 1:** Hydrophosphination of alkynes, alkenes, and carbodiimides using **1**–**6** as catalysts.^[a]^

Alkynes^[b]^		
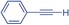	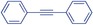	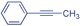
a)	b)	c)
Catalyst; yield, *E*/*Z*/α‐isomer ratio, time		
**1** 72 %, 1:8:0, 20 h **2** 95 % (78 %), 1:3:0, 20 h **3** 86 %, 1:3:0, 20 h **4** 72 %, 1:8:8, 20 h **5** 62 %, 1:12:4, 20 h **6** 62 %, 1:12:4, 20 h	**1** 98 %, 10:1, 5 h **2** 99 % (80 %), 10:1, 1 h **3** 98 %, 10:3, 3 h	**1** 99 %, 1:1:0, 6 h **2** 99 % (79 %), 1:1:0, 1 h **3** 95 %, 1:3:0, 10 h
Alkenes^[b]^		
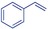	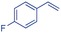	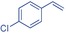
d)	e)	f)
**1** 89 %,6 h **2** 84 % (60 %), 6 h **3** 72 %, 6 h	**2** 84 %, 20 h	**2** 86 %, 18 h
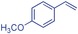		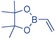
g)		h)
**2** 87 %, 20 h		**2** 93 %, 4 h
Carbodiimides^[c]^		
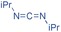		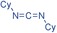
i)		j)
**2** 99 % (80 %), 0.25 h		**2** 86 %, 20 h

[a] General conditions: 0.6 mmol substrate, 0.5 mmol HPPh_2_, [D_8_]toluene. Conversions based against ^1^H NMR internal standard hexamethylcyclotrisiloxane. *E*/*Z*/α stereoselectivity based on ^31^P NMR spectra. Selected isolated yields in parenthesis. [b] 10 mol % [Al] catalyst, 110 °C. [c] 5 mol % [Al] catalyst, RT.

To gain more insight, a 1:1:3 stoichiometric reaction between [iBu_3_AlHLi]_2_, HPPh_2_, and THF, completely consumed the aluminate, giving a solution that deposited crystals of the lithium aluminum phosphide, iBu_3_AlPPh_2_Li(THF)_3_ (**2**; 41 % yield of isolated product; Figure 1 a). Phosphidoaluminate **2** results from deprotonation of HPPh_2_ by **1**, and importantly, implies this process is the first step in catalytic hydrophosphination. Crystalline **2** (Figure 1 b) is monomeric, with three THF molecules solvating Li. ^1^H DOSY NMR studies confirm it remains monomeric in [D_8_]toluene solution.^15^ Alternatively, **2** can be made by co‐complexing LiPPh_2_ with iBu_3_Al/THF.

Aware that dehydrocoupling can compete with hydrophosphination, a control reaction between HPPh_2_ and 10 mol % of **2** in [D_8_]toluene was heated at 110 °C for 20 h. Less than 15 % of HPPh_2_ had undergone dehydrocoupling to form 1,1,2,2‐tetraphenyl diphosphine (determined by ^31^P NMR spectra), signifying that this is unlikely to be a significant problem in this system. Subsequently **2** was tested as a catalyst for the hydrophosphination of alkynes under the previously optimized conditions (10 mol % [Al], [D_8_]toluene, 110 °C; Table 1). For phenylacetylene, a 95 % conversion (1:3 *E*/*Z* ratio) of the anti‐Markovnikov product was obtained after 20 h (compare 72 % using **1**), albeit with reduced *E*/*Z* stereoselectivity. By contrast, Waterman's tin catalyst Cp*_2_SnCl_2_ is poorly active for PhC≡CH (10 mol % catalyst, 18 h, 65 °C, 4 % yield).7b Using **2**, hydrophosphination is much faster with internal alkynes than terminal alkynes, with a 99 % yield (10:1 *E*/*Z* ratio) for diphenylacetylene being obtained within just 1 h (**1** takes 5 h). Similarly, 1‐phenyl‐1‐propyne fully converts into the anti‐Markonikov vinyl phosphine product within 1 h. The catalytic activity of **2** with PhC≡CPh compares favorably with the β‐diketiminato calcium amide catalyst ^DIPP^NacNacCa(HMDS)(THF), which required extended reaction times (10 mol % catalyst, 75 °C, 13 h, 94 % yield).6c However, [Ca(PPh_2_)_2_(THF)_4_] catalytically hydrophosphinated diphenylacetylene after 2 h at room temperature.6e Cui used an imino‐amidinate ligated Ca catalyst for quantitative hydrophosphination of 1‐phenyl‐1‐propyne after 5 h at 60 °C, using 5 mol % [Ca].3a

**Figure 2:**
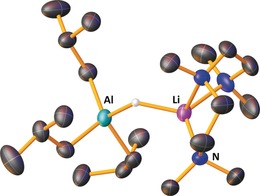
Molecular structure of iBu_3_AlHLi(PMDETA). Ellipsoids are set at 40 % probability, and disordered iBu groups, disordered PMDETA, and hydrogen atoms except hydride are omitted for clarity.

Adding a catalytic amount (30 mol %) of THF to 10 mol % of **1** resulted in hydrophosphination of diphenylacetylene within the same time as that using pre‐formed **2**, suggesting that deaggregation of dimeric **1** by THF is advantageous in catalysis. Again, attempted catalysis with unactivated 1‐hexyne or 3‐hexyne and HPPh_2_ by **2** proved unsuccessful. Deaggregation aside, the coordination shell surrounding a metal cation can play a key role in modulating the Lewis acidity of the metal, thereby providing a potential route to modify reactivity. Thus, we explored the effect of the Lewis donor on hydrophosphination of PhC≡CPh (Table 2). A range of Lewis donor additives were added to the hydrophosphination reactions of diphenylacetylene catalyzed by **1**. Adding either two equiv (with respect to the catalyst) of bidentate donor TMEDA (N,N,N′,N′‐tetramethylethylenediamine) or one equiv of 12‐crown‐4 result in quantitative conversions in 1 h, the same time as when 3 equiv of THF are used. The molecular structure of the organometallic compound in the presence of polydentate 12‐crown‐4 was determined via X‐ray crystallography as the contact ion pair structure, iBu_3_AlPPh_2_Li(12‐crown‐4), with the phosphorus atom bridging the Al and Li centers (Supporting Information). Unfortunately, owing to poor‐quality data no geometric parameters can be discussed, however the structure provides unequivocal proof of atomic connectivity. The use of isolated iBu_3_AlHLi(PMDETA) also results in quantitative product formation within 1 h (tridentate PMDETA=*N*,*N*,*N′*,*N′′N′′*‐pentamethyldiethylenetriamine). This complex was crystallographically characterized (Figure 2), but all organic ligands exhibit significant disorder which precludes a discussion of geometric parameters beyond atomic connectivity.

**Table 2:** Effect of Lewis donor upon hydrophosphination catalysis of diphenylacetylene.^[a]^ 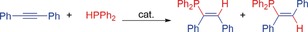

Lewis donor additive	Time [h]	Yield [%]	*E*:*Z* ratio
none [iBu_3_AlHLi]_2_, **1**	5	98	10:1
**1**+3 equiv THF	1	99	10:1
iBu_3_AlHLi(PMDETA)	1	99	2:1
**1**+1 equiv 12‐crown‐4	1	99	5:1
**1**+2 equiv TMEDA	1	99	19:1
**1**+1 equiv Me_6_‐TREN	3	95	10:1
**1**+2 equiv dppe	5	95	16:1
iBu_3_AlPPh_2_Li(THF)_3_	1	99	10:1
**2**+3 equiv PPh_3_	1.5	99	4:1

[a] General conditions: 0.6 mmol substrate, 0.5 mmol HPPh_2_, [D_8_]toluene, 10 mol % [Al] catalyst, 110 °C. Conversions based against ^1^H NMR internal standard hexamethylcyclotrisiloxane. *E*/*Z*/α stereoselectivity based on ^31^P NMR spectra.

Interestingly, the *E*/*Z*‐isomer ratio is dependent on the donor used. PMDETA and 12‐crown‐4 are less selective (*E*/*Z* 2:1; and 5:1 respectively, versus 10:1 with 3 THF), whereas 2 TMEDA donors result in enhanced *E*/*Z*‐selectivity of 19:1. Adding one equiv of bulky tetradentate Me_6_‐TREN, takes 3 h for quantitative conversion (*E*/*Z* 10:1). Adding two equiv of bidentate dppe (diphenylphosphinoethane) results in conversion in 5 h, albeit with good *E*/*Z* selectivity (16:1). Interestingly it appears that when two bidentate donors are added, TMEDA or dppe, marked improvements in selectivity occur. Finally, adding three equiv of PPh_3_ to preformed **2** results in both slower catalysis (1.5 h) and poorer selectivity (*E*/*Z* 4:1) than those observed with the THF variant, indicating the phosphine Lewis donor may inhibit the hydrophosphination process.

Next, the more challenging hydrophosphination of alkenes was examined using **2** (Table 1). Styrene undergoes hydrophosphination in 6 h, at 110 °C, yielding 84 % of the anti‐Markovnikov product. Halo‐substituted styrenes are also tolerated (Table 1, entries e,f). 4‐Vinyl anisole undergoes hydrophosphination to the alkyl phosphine product in 87 % yield after 20 h at 110 °C. Bulkier substrates such as α‐methyl styrene, *trans*‐β‐methyl styrene, and the less activated alkene 1‐hexene did not undergo hydrophosphination with **2** as the catalyst. Similar failures with both Ca and Sn based catalysts have been noted for these substrates.6c, 7b Hydrophosphination of vinyl boronic acid pinacol ester (vinyl Bpin) achieved a 93 % yield after 4 h at 110 °C, producing linear phosphine boronic ester Ph_2_P(CH_2_)_2_Bpin. To our knowledge this is the first time Ph_2_P(CH_2_)_2_Bpin has been made by a hydrophosphination route, since earlier published methods required hydroboration of diphenyl vinyl phosphine.^16^

Phosphidoaluminate **2** is also an able catalyst for hydrophosphination of carbodiimides at room temperature. Thus, using 5 mol % catalyst loading (Table 1, entries i–j), diisopropylcarbodiimide is converted fully into the phosphaguanidine product within 15 min, while bulkier dicyclohexylcarbodiimide required 20 h to achieve 86 % conversion. Hill reports quantitative yields for diisopropyl and dicyclohexyl carbodiimides within 1 h and 4 h, respectively, using 2 mol % Ca(HMDS)_2_ as catalyst, also at room temperature. Significantly longer reaction times were seen when using ^DIPP^NacNacCa(HMDS)(THF) as a catalyst (1.5 mol %; iPr, 6 h, 99 %: Cy, 28 h, 85 %).6d KHMDS is also found to be a good catalyst for carbodiimides requiring low catalyst loadings and short reaction times,6a while a sodium magnesiate also catalyzes hydrophosphination of carbodiimides.6f

**Scheme 1:**
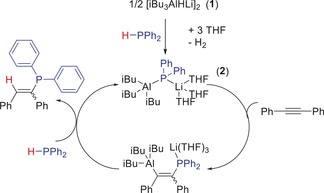
Proposed reaction mechanism for hydrophosphination of diphenylacetylene by pre‐catalyst **1**, showing formation of active species **2**.

Attempting to pinpoint the active catalyst, four compounds, LiPPh_2_ (**3**), iBu_3_Al (**4**), iBu_2_AlH (**5**), and iBu_2_AlPPh_2_ (**6**),^17^ were screened for catalytic viability using PhC≡CH as a model substrate (Table 1). Using LiPPh_2_ as a catalyst yields 86 % conversion to the vinyl phosphine after 20 h, with anti‐Markovnikov regioselectivity, similar to that of **2**. Compounds **4**–**6** afford different product regio‐ and stereoselectivities as well as lower yields for PhC≡CH hydrophosphination. Interestingly when **4** is used as catalyst (72 %; 1:8:8 *E*/*Z*/*α*) the major isomer products are the *Z*‐anti‐Markovnikov isomer, and equally the Markovnikov (α‐isomer; Ph(Ph_2_P)C=CH_2_). In contrast, **2** does not give any appreciable α‐isomer, suggesting **2** is not disproportionating in solution at high temperature into LiPPh_2_ and iBu_3_Al. To ascertain whether LiPPh_2_ is implicated in the catalytic profile, we conducted another stoichiometric reaction (Supporting Information). Monitoring the reaction of **2** and PhC≡CPh in [D_8_]toluene by ^31^P NMR shows that after 2 h at 110 °C full consumption of **2** occurs with concomitant growth of two new signals at *δ*=3.4 and −16.5 ppm. Unable to isolate these species despite several attempts, we tentatively assign them as two isomers resulting from insertion of diphenylacetylene into **2**. Subsequent addition of HPPh_2_ and further heating at 110 °C allows for product formation (*δ*=8.9 ppm (*E*‐isomer) and *δ*=−7.3 ppm (*Z*‐isomer)) and regeneration of **2** (*δ*=−49.3 ppm). We rationalize the intermediate (at *δ*=3.4 ppm) reacts onwards to form the *E*‐stereoisomer as it is consumed faster than the other intermediate. Significantly, there are no resonances corresponding to LiPPh_2_ in the spectrum (*δ*=−52 ppm) further reinforcing the view that **2** is the catalytically active species. We propose a catalytic cycle (Scheme 1) that begins by deprotonation of HPPh_2_ by [iBu_3_AlHLi]_2_, releasing H_2_ and forming compound **2**. Next, a substrate molecule inserts into the Al−P bond. Subsequent protonolysis by a second equiv of HPPh_2_ accesses the hydrophosphinated product whilst regenerating the active catalytic species **2**.

Since after the facile room temperature deprotonation step, alkyne insertion and protonolysis are the other key steps, we performed a deuterium labeling study to investigate the cycle further. Catalytic hydrophosphination between PhC≡CPh and DPPh_2_ favored formation of the *E*‐stereoisomer and deuterium was incorporated into the vinyl phosphine product, Ph(Ph_2_P)C=C(D)Ph, as confirmed by ^2^H NMR spectra and GC‐MS (Supporting Information). Also, in a stoichiometric reaction between [iBu_3_AlHLi]_2_ and DPPh_2_, HD was detected in the ^1^H NMR spectrum (triplet at *δ*=4.45 ppm, ^1^J=42.8 Hz), confirming the initial deprotonation step.

A kinetic isotope effect experiment (KIE) was conducted for the hydrophosphination of diphenylacetylene by recording the reaction profile in duplicate for HPPh_2_ and DPPh_2_ at 100 °C, in [D_8_]toluene, with 10 mol % of **2**. By monitoring the consumption rate of phosphine by ^31^P NMR, rates were obtained, and in each case the overall reaction rate is pseudo first order. From these rates a KIE of 1.38±0.13 was determined (Supporting Information). This is a small value, compared with previous reports, and suggests that cleavage of the P−H bond is only involved to a minor extent in the rate determining step.7b This also indicates that alkyne insertion into **2** is rate‐determining, which given the rather congested structure of **2** and bulky nature of the alkyne is unsurprising.

**Figure 3:**
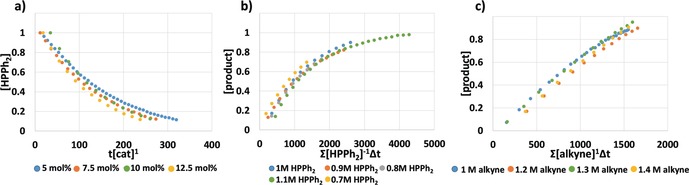
Variable time normalization analysis (VTNA) plots illustrating the order in a) catalyst (first order); b) phosphine (inverse first order); c) diphenylacetylene (first order).

Next, we conducted a kinetic analysis of the reaction using the variable time normalization analysis (VTNA) method reported by Burés, allowing us to obtain valuable mechanistic detail under synthetically relevant conditions to three half‐lives (Figure 3 and the Supporting Information).^18^ The reaction order with respect to [catalyst] was determined by conducting reactions using different catalyst concentrations, while keeping [alkyne] and [phosphine] constant. These data showed the reaction rate increases with increasing [catalyst], and that the order in catalyst is one. This situation is consistent with the reaction proceeding via a monomeric rate determining step during the reaction. Variation of [phosphine] under synthetically relevant conditions revealed that increasing concentration of phosphine inhibits the reaction, giving a phosphine order of −1. This inhibition likely results from pre‐coordination of the phosphine, blocking off the alkyne for insertion. Also, we have already established that bulky mono‐ and bidentate phosphines slow down reactivity in our Lewis donor study (see above). Lastly, variation of [alkyne] revealed a first order dependence in [alkyne], indicating that the alkyne is involved in the rate‐limiting step.

Finally, to reinforce our experimental insight, we turned to DFT calculations. Run on the full system with the internal alkyne, diphenylacetylene, used as the model substrate, the calculations were performed at the B3LYP‐D3/19 6‐311G(d,p)^20^ level of theory employing a continuum solvent with the dielectric constant of toluene within the IEFPCM model.^21^ The relative stability of the formation of **2** from [iBu_3_AlHLi]_2_ with 2 HPPh_2_ and 6 THF molecules was initially investigated. Formation of the catalyst (**2**) is thermodynamically favorable despite the entropic penalty associated with THF coordination, with a calculated Δ*G*=−63.8 kcal mol^−1^ (Δ*H*=−130.8 kcal mol^−1^). The activation barrier for the formation of the catalyst was challenging to isolate as a result of the complex potential energy surface associated with the large dimer species. However, a bond scan along the coordinate associated with the formation of H_2_ provided an indicative barrier (ΔE*) of about 38 kcal mol^−1^, which would be achievable under the reaction conditions and lead irreversibly to **2** given the exothermic nature of this step. In contrast to the induction step, the first step in the catalytic cycle (adding diphenylacetylene to **2**) is mildly endergonic for both the *E* and *Z* isomers of the intermediate shown in Scheme 1. However, the *E* isomer is more stable in the intermediate state of the reaction (Δ*G*=6.9 kcal mol^−1^), with the *Z*‐isomer (Δ*G*=8.3 kcal mol^−1^) being further destabilized by 1.4 kcal mol^−1^ relative to the *E*‐isomer. Finally, generation of the product and reformation of the catalytic species occurs in an exergonic reaction. In this step, the formation of the *Z*‐isomer (Δ*G*=−25.3 kcal mol^−1^) is favored over the *E*‐isomer (Δ*G*=−20.4 kcal mol^−1^). The reversal of the relative stabilities of the isomers in the intermediate state versus the product state suggests that the formation of the intermediate is deterministic for the final product distribution, which favors the experimentally determined *E*‐isomer. The rate‐limiting step for the reaction could not be located as the calculation of transition states proved elusive for these bulky compounds. However, the relative stabilities of the intermediates and products determined for this pathway indicate that the mechanism proposed is achievable under the reaction conditions employed.

## Conclusion

Previously lithium aluminates have been shown to be active catalysts for hydroboration of aldehydes, ketones, imines, and acetylenes. This new study extends the catalytic chemistry of these bimetallic main‐group compounds by reporting the first example of Al‐catalyzed hydrophosphination of alkynes, alkenes, and carbodiimides, using the lithium aluminate (pre)catalyst [iBu_3_AlHLi]_2_. A mechanism is proposed for the alkyne catalysis, elucidated by stoichiometric reactions, thought to proceed via formation of the crystallographically defined lithium aluminum phosphide, iBu_3_AlPPh_2_Li(THF)_3_ (**2**), followed by insertion of the alkyne into the Al−P bond, then protonolysis of a second equiv of the phosphine to generate the vinylphosphine product and regenerate the catalyst. While intuitively the formation of an anionic aluminum center saturated by four anionic ligands as in an aluminate might be expected to have insufficient Lewis acidity to engage in hydrophosphination processes, it is clear from the different results obtained using a number of Lewis donor solvent molecules that the presence of the lithium helps to circumvent this apparent handicap so pointing to bimetallic synergistic behavior.

## Experimental Section

Full experimental characterization and synthetic procedures are described in the Supporting Information.

**Synthesis of iBu_3_AlPPh_2_Li(THF)_3_ (2): Method (a)**: HPPh_2_ (0.34 mL; 2 mmol) was added to a stirred solution of [iBu_3_AlHLi]_2_ (0.412 g; 1 mmol) in hexane (10 mL) and the reaction stirred 1 h. THF (0.5 mL; 6 mmol) was added and then the volatiles were removed. The residue was taken up in hexane (5 mL) and toluene (1 mL). Subsequent cooling to −30 °C yielded the desired product as pale‐yellow crystals. Crystalline yield 0.494 g; 0.82 mmol; 41 %. **Method** (**b)**: nBuLi (0.63 mL; 1.6 m/hexane; 1 mmol) was added dropwise to a stirred solution of HPPh_2_ (0.17 mL; 1 mmol) in hexane (5 mL) and the resulting bright yellow suspension stirred for 1 h. Addition of iBu_3_Al (1 mL; 1 m/hexane; 1 mmol) generated a clear pale‐yellow solution, which was stirred for 1 h. THF (0.3 mL; 3 mmol) was added and the pale‐yellow solution cooled at −30 °C overnight. Crystalline yield 0.150 g; 0.25 mmol; 24 %. ^1^H NMR (400.1 MHz, [D_8_]toluene, 300 K): *δ*=0.48 (d of d, *J*=6.93 Hz, 2.88 Hz, 6 H, iBu CH_2_); 1.31 (d, *J*=6.29 Hz, 18 H, iBu CH_3_); 1.41 (m, 12 H, THF CH_2_); 2.26 (m, 3 H, iBu CH); 3.44 (m, 12 H, THF CH_2_); 7.00 (m, 2H (overlapping solvent), Ph); 7.17 (m, 4H (overlapping solvent), Ph); 7.14 (m, 4 H, Ph) ppm. ^31^P NMR (104.2 MHz, [D_8_]toluene, 300 K): *δ*=−49.2 ppm. ^13^C{^1^H} NMR (151 MHz, [D_8_]toluene, 300 K): *δ*=25.4 (THF CH_2_); 25.5+25.6 (iBu CH_2_); 28.3 (iBu CH); 29.5 (iBu CH_3_); 68.6 (THF CH_2_); 124.9 (Ar C−H); 127.3 (d, *J*=6.17 Hz, Ar C−H); 134.0 (d, *J*=13.04 Hz, Ar C−H); 144.1 (d, *J*=13.08 Hz, *ipso* Ar) ppm. ^7^Li NMR (155.5 MHz, [D_8_]toluene, 300 K): *δ*=0.21 (s) ppm. ^27^Al NMR: no signal was observed.

**General catalytic reaction**: The desired catalyst loading was added to 0.5 mL of [D_8_]toluene solution (unless alternative solvent specified) containing the substrate precursor (0.6 mmol) and HPPh_2_ (0.5 mmol, 0.09 mL). The reaction mixture was transferred to a sealed J. Young's tap NMR tube and the reaction was regularly monitored by ^1^H and ^31^P NMR spectroscopy until the formation of the products was completed as determined by integration versus an internal capillary standard (hexamethylcyclotrisiloxane). For alkynes and alkenes, the hydrophosphination catalysis was performed at 110 °C with 10 mol % [Al] catalyst loading. For carbodiimides the hydrophosphination catalysis was performed at room temperature with 5 mol % [Al] catalyst loading. The yields reported are based on ^1^H NMR and ^31^P relative to the internal standard. In all cases, the bulk of the NMR solution can be attributed to either product compounds or starting materials. Yields of isolated product are provided for example substrates, isolated via either recrystallization methods or column chromatography, as reported in the Supporting Information.

## Conflict of interest

The authors declare no conflict of interest.

## Supporting information

As a service to our authors and readers, this journal provides supporting information supplied by the authors. Such materials are peer reviewed and may be re‐organized for online delivery, but are not copy‐edited or typeset. Technical support issues arising from supporting information (other than missing files) should be addressed to the authors.

Supplementary
