# Supplementary material for: Lithium‐Aluminate‐Catalyzed Hydrophosphination Applications
Source: Angew Chem Int Ed Engl. 2019 Jul 25;58(35):12291–6. doi: 10.1002/anie.201906807 (PMC6771573; doi:10.1002/anie.201906807)
Supplement: Supplementary file 1 — Supplementary [file ANIE-58-12291-s001.pdf]

Angewandte Chemie
Eine Zeitschrift der Gesellschaft Deutscher Chemiker

Supporting Information

# Lithium-Aluminate-Catalyzed Hydrophosphination Applications

*Victoria A. Pollard, Allan Young, Ross McLellan, Alan R. Kennedy, Tell Tuttle, and Robert E. Mulvey**

anie_201906807_sm_miscellaneous_information.pdf

# Contents

General Experimental: ..... 1
NMR spectroscopy ..... 1
X-ray crystallography ..... 1
GC-MS ..... 1
X-ray Crystallographic Geometric Information ..... 2
Table S2: Crystal structure data and refinement details for compound **2,** $iBu_3AlPPh_2Li$(12-crown-4) and $iBu_3AlHLi$(PMDETA). ..... 5
Synthesis of Compounds: ..... 6
Synthesis of $iBu_3AlPPh_2Li(THF)_3$, **2** ..... 6
Synthesis of $iBu_3AlPPh_2Li$(12-crown-4) ..... 9
Synthesis of $iBu_3AlHLi$(PMDETA) ..... 12
Stoichiometric Reactions ..... 15
Hydrophosphination Catalysis ..... 19
General Catalytic Reaction: ..... 19
Heating $HPPh_2$ in the presence of **2** ..... 19
Solvent Screen ..... 20
Hydrophosphination of alkynes ..... 22
Hydrophosphination of alkenes ..... 40
Hydrophosphination of carbodiimides ..... 50
Unreactive substrates tested ..... 54
Deuterium labelling experiment ..... 54
Donor Screening Experiments ..... 59
Kinetic Isotope Effect (KIE) experiment ..... 67
Kinetics ..... 69
DFT Calculations ..... 73
Control reactions ..... 74

## General Experimental:

All reactions and manipulations were performed under a protective argon atmosphere using either standard Schlenk techniques or glove box techniques. Hexane, THF, and toluene were dried by heating to reflux over sodium benzophenone ketyl and then distilled under nitrogen prior to use. $C_6D_6$, $d_8$-toluene, $d_8$-THF and $CD_2Cl_2$ were degassed by freeze-pump-thaw methods and stored over activated 4 Å molecular sieves. All reagents were purchased from commercial sources and used as received, unless stated otherwise. Compounds $[iBu_3AlHLi]_2$ (**1**), [1] and $[iBu_2AlPPh_2]_2$ (**6**), [2] were prepared according to literature methods. $DPPh_2$ was prepared according to literature methods, resulting in 90 % deuterium incorporation.[3]

## NMR spectroscopy

NMR spectra were recorded on a Bruker AVIII 400 or AV 400 MHz spectrometer operating at 400.13 MHz for $^{1}H$, 155.47 MHz for $^{7}Li$, 162.0 MHz for $^{31}P$, 104.2 MHz for $^{27}Al$, 128.3 MHz for $^{11}B$ and 100.62 MHz for $^{13}C$. All $^{13}C$ spectra were proton decoupled. $^{1}H$, $^{13}C\{^{1}H\}$, $^{7}Li$, $^{31}P$, $^{27}Al$ and $^{11}B$ chemical shifts are expressed in parts per million (δ, ppm) and referenced to residual solvent peaks. $^{1}H$ DOSY measurements were recorded on an AV 400 spectrometer operating at 400.13 MHz, using the pulse program ledbpgp2s. Tetraphenylnaphthalene has been used as reference standard in the DOSY measurements.[4] KIE measurements were recorded on a Bruker AVII 600 spectrometer operating at 243 MHz for $^{31}P$.

## X-ray crystallography

Data for **2** was collected on an Oxford Diffraction Xcalibur E instrument with graphite-monochromated Mo Kα (λ = 0.71073 Å) radiation. Data collection and processing used Rigaku and Bruker software.[5] All structures were solved and refined to convergence on $F^2$ for all independent reflections by the full-matrix least squares method using SHELXL-2014/7,[5a, 6] or by the Gauss Newton algorithm using $OLEX^2$.[7] All non-hydrogen atoms were refined using anisotropic thermal parameters. Selected crystallographic data are shown in Table S1 and full details in .cif format are available from CCDC (1906536 – 1906538).

## GC-MS

GC-MS data were acquired on an Agilent Technologies 7890A GC system fitted with a Restek Rxi-5Sil column (30 m, 0.25 mm ID, 0.25 µm) and coupled to an Agilent mass spectrometer using either chemical ionisation (methane) or electron impact ionisation. Helium was used as the carrier gas (1 mL $min^{-1}$). In all cases, an inlet temperature of 320 °C and the following oven temperature gradient were used: 4 min at 40 °C; ramp to 320 °C at 20 °C $min^{-1}$; hold for 10 min (total run time of 28 min).

## X-ray Crystallographic Geometric Information

### Molecular structure of $iBu_3AlPPh_2Li(THF)_3$, **2**

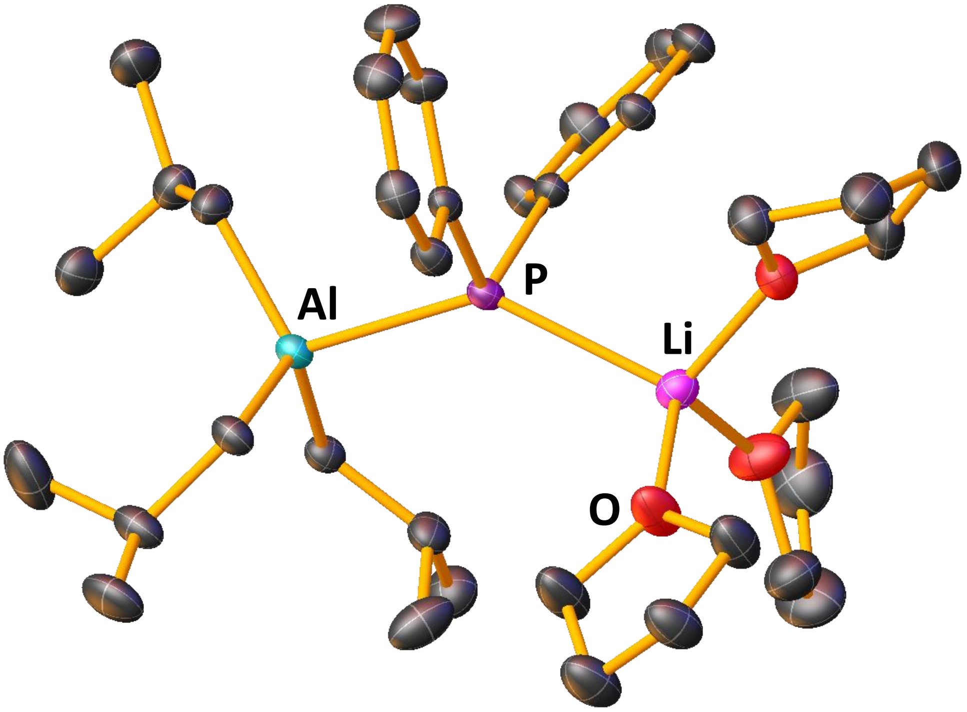


**Figure S1:** Molecular structure of $iBu_3AlPPh_2Li(THF)_3$, **2**. Thermal ellipsoids are drawn at 40% probability and hydrogen atoms and disorder in THF have been omitted for clarity.

**Table S1:** Selected bond lengths (Å) and bond angles (°) for $iBu_3AlPPh_2Li(THF)_3$:

| Bond lengths (Å) | | Bond angles (°) | |
|---|---|---|---|
| Al1 – P1 | 2.4698(13) | C1 – Al1 – P1 | 99.65(12) |
| Al1 – C1 | 1.999(4) | C1 – Al1 – C5 | 113.81(16) |
| Al1 – C5 | 2.005(4) | C1 – Al1 – C9 | 116.03(8) |
| Al1 – C9 | 2.007(4) | C5 – Al1 – P1 | 106.14(12) |
| Li1 – P1 | 2.596(6) | C5 – Al1 – C9 | 112.90(16) |
| Li1 – O1 | 1.937(7) | C9 – Al1 – O1 | 106.60(12) |
| Li1 – O2 | 1.927(7) | O1 – Li1 – P1 | 110.9(3) |
| Li1 – O3 | 1.934(7) | O2 – Li1 – P1 | 110.4(3) |
| | | O2 – Li1 – O1 | 108.3(3) |
| | | O2 – Li1 – O3 | 105.9(3) |
| | | O3 – Li – P1 | 113.8(3) |
| | | O3 – Li1 – O1 | 107.2(3) |
| | | Al1 – P1 – Li1 | 139.08(16) |
| | | C13 – P1 – Al1 | 101.65(11) |
| | | C13 – P1 – Li1 | 102.88(19) |
| | | C13 – P1 – C19 | 103.93(16) |

| | | | |
|---|---|---|---|
| | | C19 – P1 – Al1 | 108.44(12) |
| | | C19 – P1 – Li1 | 96.77(18) |

## Molecular structure of $iBu_3AlPPh_2Li$(12-crown-4)

Due to the quality of the X-ray crystallographic data obtained, no geometrical parameters further than atomic connectivity can be discussed at this time.

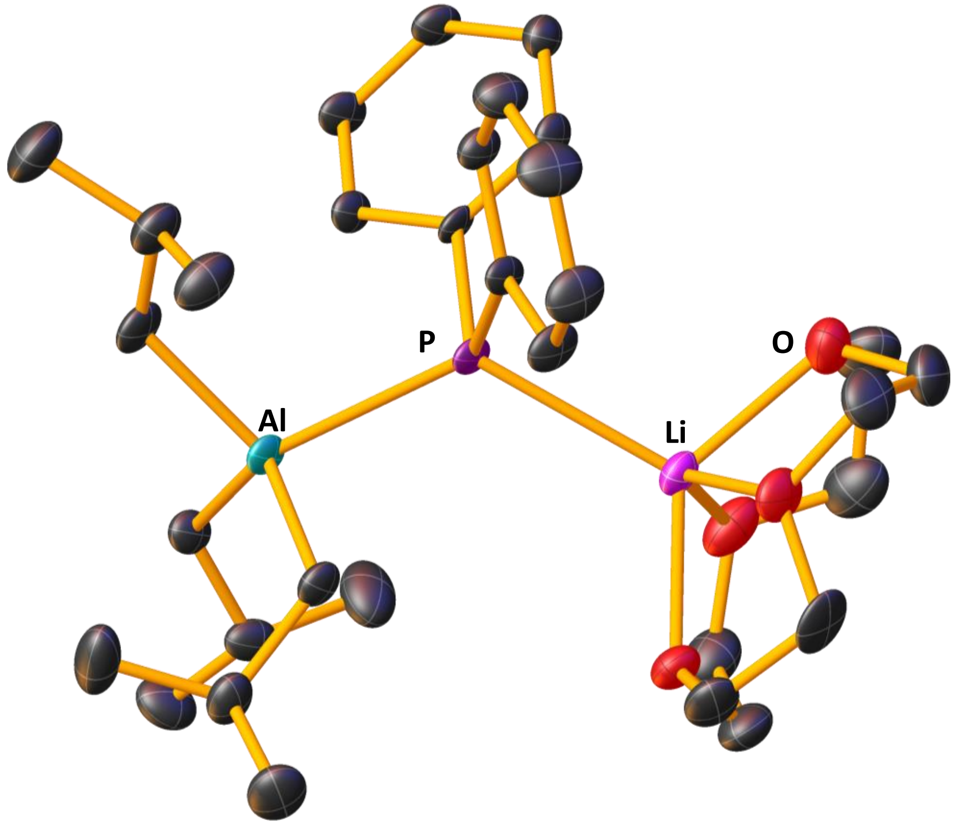


**Figure S2:** Molecular structure of $iBu_3AlPPh_2Li$(12-crown-4). Thermal ellipsoids are drawn at 40% probability and disorder and hydrogen atoms have been omitted.

## Molecular structure of $iBu_3AlHLi(PMDETA)$

Due to the quality of the X-ray crystallographic data obtained, no geometrical parameters further than atomic connectivity can be discussed at this time.

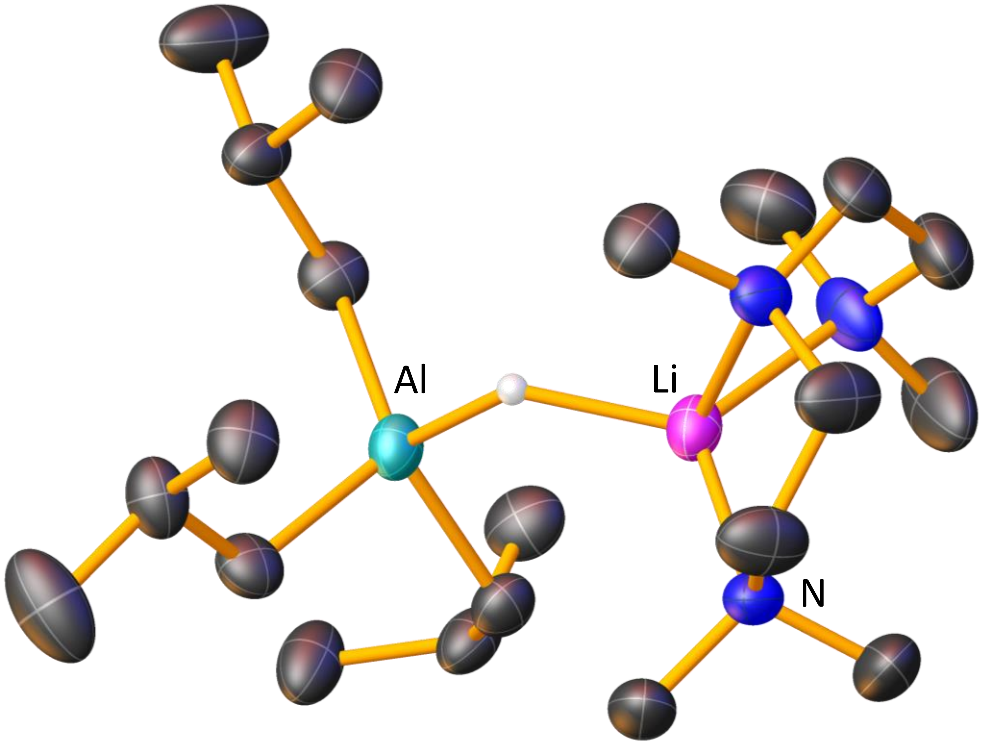


**Figure S3:** Molecular structure of $iBu_3AlHLi(PMDETA)$. Thermal ellipsoids are drawn at 40% probability and disorder and hydrogen atoms have been omitted.

Table S2: Crystal structure data and refinement details for compound **2,** $iBu_3AlPPh_2Li$(12-crown-4) and $iBu_3AlHLi$(PMDETA).

| **Compound** | $iBu_3AlPPh_2Li(THF)_3$, **2** | $iBu_3AlPPh_2Li$(12-crown-4) | $iBu_3AlHLi$(PMDETA) |
|---|---|---|---|
| **Empirical formula** | $AlLiPO_3C_{36}H_{61}$ | $AlLiPO_4C_{32}H_{53}$ | $C_{21}H_{52}AlLiN_3$ |
| **Molecular mass** | 606.73 | 566.63 | 379.56 |
| **Crystal system** | Monoclinic | Triclinic | Orthorhombic |
| **a/ Å** | 18.0340(9) | 9.1586(6) | 16.6792(5) |
| **b/ Å** | 11.3773(6) | 11.8525(7) | 15.6104(5) |
| **c/ Å** | 18.6888(10) | 16.5468(10) | 10.2931(3) |
| **α/ °** | 90 | 97.746(5) | 90 |
| **β/ °** | 98.341(5) | 104.970(6) | 90 |
| **γ/ °** | 90 | 101.162(5) | 90 |
| **V/ Å$^3$** | 3794.0(3) | 1669.74(18) | 2680.00(14) |
| ***Z*** | 4 | 2 | 4 |
| **λ/ Å** | MoKα (λ = 0.71073 Å) | CuKα (λ = 1.54184 Å) | Cu Kα (λ = 1.54184) |
| **Measured reflections** | 19956 | 12010 | 6327 |
| **Unique reflections** | 7944 | 6280 | 2381 |
| **$R_{int}$** | 0.0402 | 0.0931 | 0.0574 |
| **Observed rflns [I > 2σ(I)]** | 5775 | 4564 | 2773 |
| **GOOF** | 1.008 | 1.244 | 1.059 |
| **R [on *F*, obs rflns only]** | 0.0525 | 0.1154 | 0.0676 |
| **ωR [on $F^2$, all data]** | 0.1107 | 0.3509 | 0.1906 |
| **Largest diff. peak/hole e/Å$^{-3}$** | 0.31/-0.25 | 1.40/-1.01 | 0.53/-0.30 |

## Synthesis of Compounds:

### Synthesis of $iBu_3AlPPh_2Li(THF)_3$, **2**

*Method a)*

To a stirred solution of $[iBu_3AlHLi]_2$ (0.412 g; 1 mmol) in hexane (10 mL) was added $HPPh_2$ (0.34 mL; 2 mmol) and the reaction stirred 1 h. THF (0.5 mL; 6 mmol) was added then the volatiles were removed. The residue was taken up in hexane (5 mL) and toluene (1 mL). Subsequent cooling to – 30 °C yielded the desired product as pale-yellow crystals. Crystalline yield 0.494 g; 0.82 mmol; 41 %.

*Method b)*

To a stirred solution of $HPPh_2$ (0.17 mL; 1 mmol) in hexane (5 mL) was added dropwise nBuLi (0.63 mL; 1.6 M/hexane; 1 mmol) and the resulting bright yellow suspension stirred for 1 h. Addition of $iBu_3Al$ (1 mL; 1 M/hexane; 1 mmol) generated a clear pale-yellow solution, which was stirred for 1 h. THF (0.3 mL; 3 mmol) was added and the pale-yellow solution cooled at – 30 °C overnight. Crystalline yield 0.150 g; 0.25 mmol; 24%.

$^1H$ NMR (400.1 MHz, $d_8$-toluene, 300 K): δ 0.48 (d of d, J = 6.93 Hz, 2.88 Hz, 6H, iBu $CH_2$); 1.31 (d, J = 6.29 Hz, 18H, iBu $CH_3$); 1.41 (m, 12H, THF $CH_2$); 2.26 (m, 3H, iBu CH); 3.44 (m, 12H, THF $CH_2$); 7.00 (m, 2H [overlapping solvent], Ph); 7.17 (m, 4H [overlapping solvent], Ph); 7.14 (m, 4H, Ph) ppm.

$^{31}P$ NMR (104.2 MHz, $d_8$-toluene, 300 K): δ - 49.2 ppm

$^{13}C\{^1H\}$ NMR (151 MHz, $d_8$-toluene, 300 K): δ 25.4 (THF $CH_2$); 25.5 + 25.6 (iBu $CH_2$); 28.3 (iBu CH); 29.5 (iBu $CH_3$); 68.6 (THF $CH_2$); 124.9 (Ar C–H); 127.3 (d, J = 6.17 Hz, Ar C–H); 134.0 (d, J = 13.04 Hz, Ar C–H); 144.1 (d, J = 13.08 Hz, ipso Ar) ppm.

$^7Li$ NMR (155.5 MHz, $d_8$-toluene, 300K): δ 0.21 (s) ppm

$^{27}Al$ NMR: no signal was observed

Satisfactory elemental analysis was not obtained after multiple attempts due to the sensitivity of this compound.

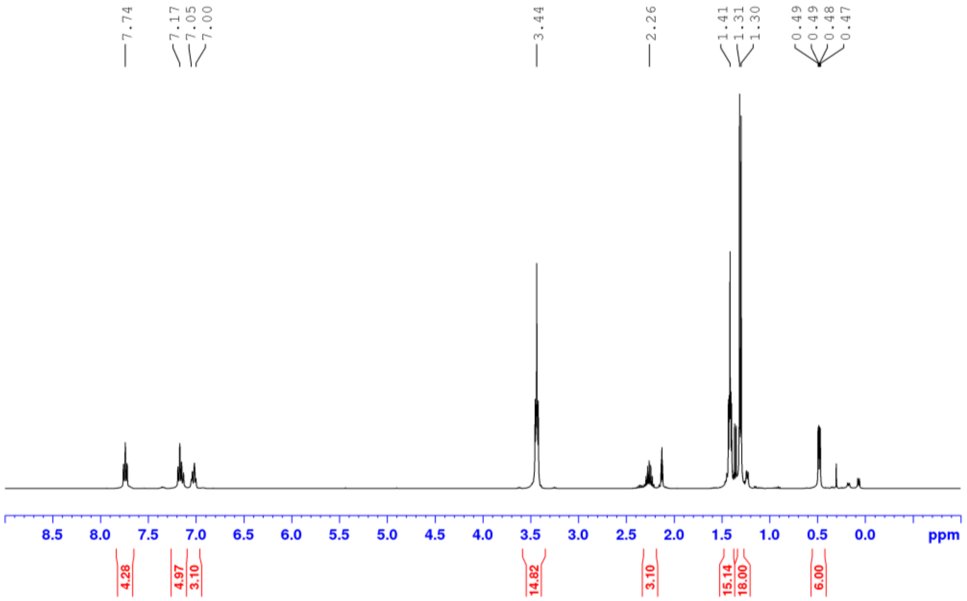


**Figure S4:** $^{1}$H NMR spectrum of $iBu_3AlPPh_2Li(THF)_3$, **2**, in $d_8$-toluene

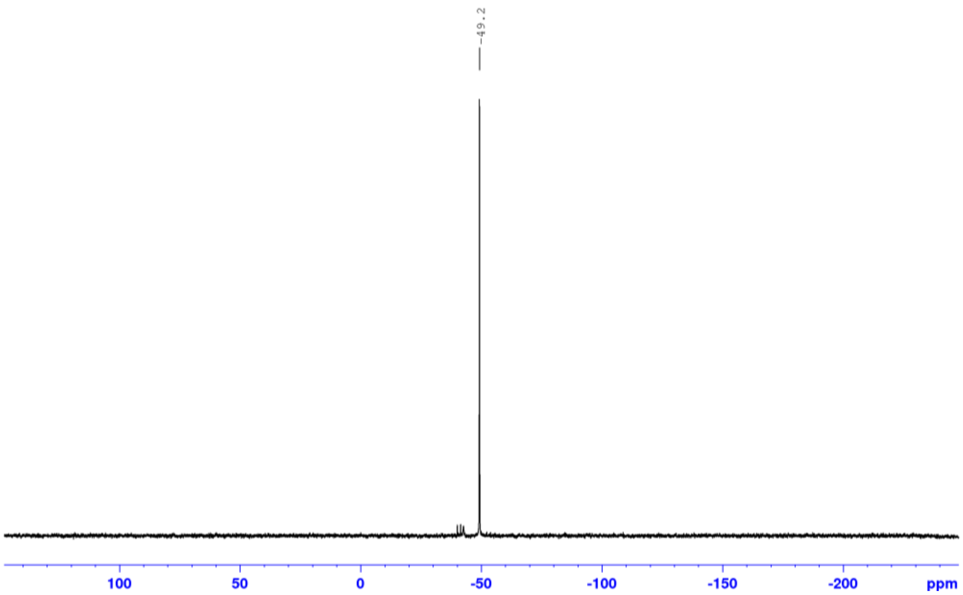


**Figure S5:** $^{31}$P NMR spectrum of $iBu_3AlPPh_2Li(THF)_3$, in $d_8$-toluene

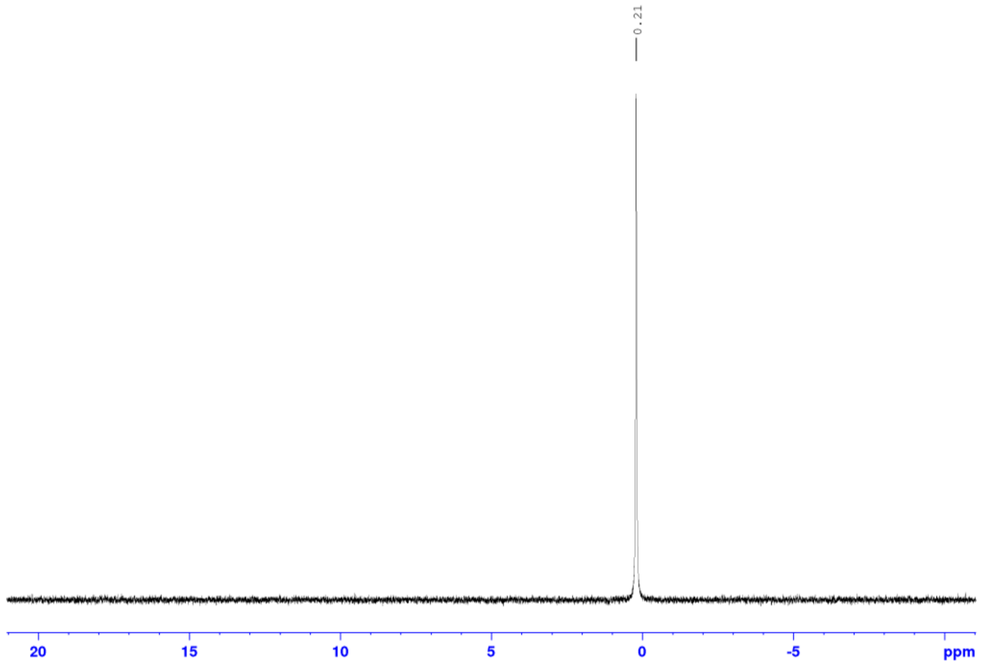


**Figure S6:** $^{7}Li$ NMR spectrum of $iBu_3AlPPh_2Li(THF)_3$, in $d_8$-toluene

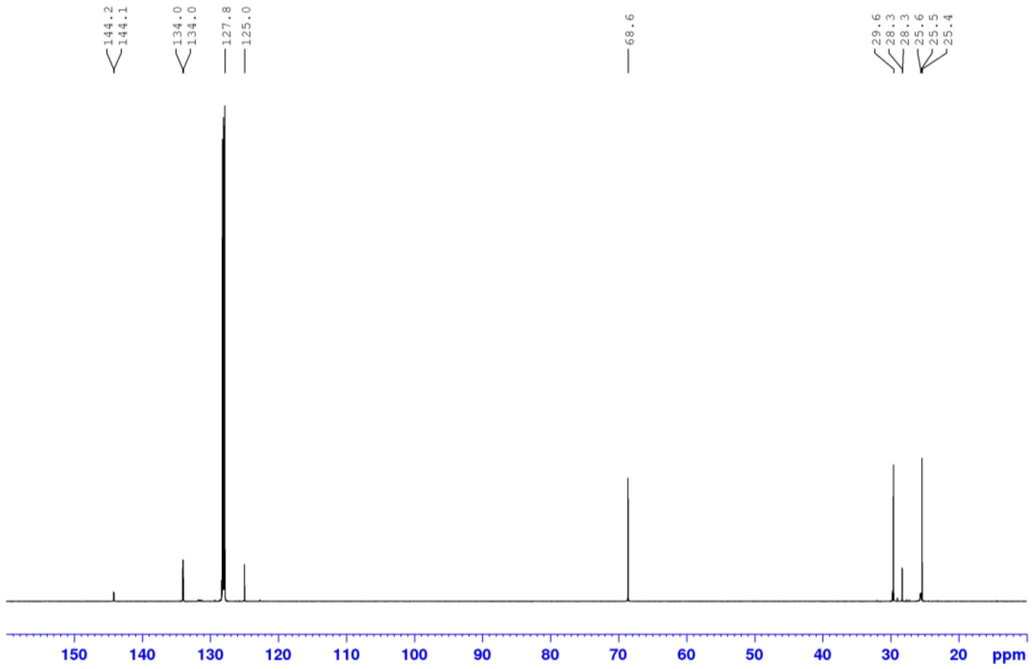


**Figure S7:** $^{13}C\{^{1}H\}$ NMR spectrum of $iBu_3AlPPh_2Li(THF)_3$, in $C_6D_6$

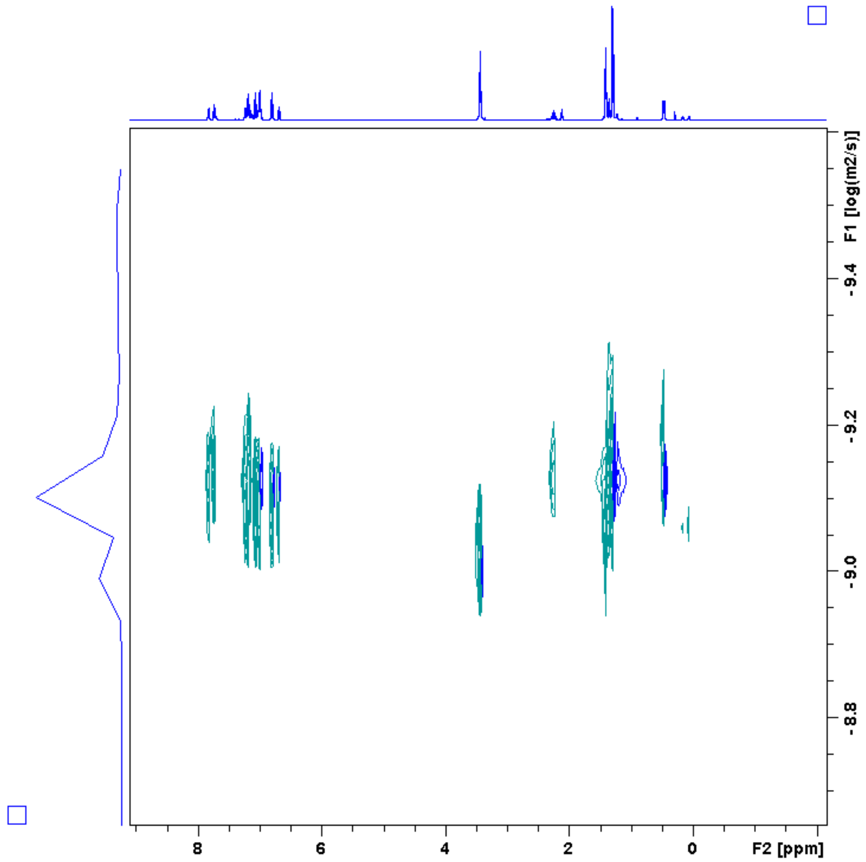


**Figure S8:** $^{1}H$ DOSY NMR spectrum for **2**, in $d_8$-toluene

**Table S3:** $^{1}H$ DOSY NMR data for $iBu_3AlPPh_2Li(THF)_3$, **2**

| Compound | D [$m^2s^{-1}$] | $MW_{calc}$ [g $mol^{-1}$] | $MW_{est}$ [g $mol^{-1}$] | Error [%] |
|---|---|---|---|---|
| $iBu_3AlPPh_2Li(THF)_3$ | $6.09x10^{-10}$ | 566 | 603 | 7 |
| Tetraphenylnaphthalene | $6.429x10^{-10}$ | 432 | 432 | 0 |

The diffusion coefficient of $iBu_3AlPPh_2Li(THF)_3$, **2** in $d_8$-toluene indicates a molecular weight of 603.75 g $mol^{-1}$. This result is consistent with the calculated molecular weight for monomeric structure $iBu_3AlPPh_2Li(THF)_3$, **2**. Note that the diffusion coefficients corresponding to the THF signals suggest some degree of fluxionality between free and bonded THF molecules.

## Synthesis of $iBu_3AlPPh_2Li$(12-crown-4)

To a solution of *n*BuLi (0.63 mL; 1.6M/hexane; 1 mmol) in hexane (5 mL) was added $HPPh_2$ (0.17 mL; 1 mmol) dropwise at room temperature and the resulting yellow suspension stirred for 1 hour. *i*$BuAl_3$ (1 mL; 1 M/hexane; 1 mmol) was then added dropwise forming a clear solution, which was stirred for 30 minutes.

12-crown-4 (0.16 mL; 1 mmol) was added which formed a pale yellow suspension. Solvents were removed *in vacuo* and toluene (4 mL) was added. Gentle heating generated a pale yellow solution. Upon slow cooling a crop of colourless crystals suitable for X-ray diffraction was obtained. (Yield 0.218g; 0.38 mmol; 38 %).

$^{1}H$ NMR (400.1 MHz, $C_6D_6$, 300 K): δ 0.59 (dd, J = 6.77 Hz + 2.88 Hz, 6H, *i*Bu $CH_2$); 1.39 (d, J = 6.46 Hz, 18H, *i*Bu $CH_3$); 2.40 (sept., J = 6.51 Hz, 3H, *i*Bu CH); 2.87 (br. s., 16H, 12-crown-4 $CH_2$); 7.01 – 7.08 (m, 2H, Ar CH); 7.24 (br. t, J = 7.49 Hz, 4H, Ar CH); 7.90 (br. t, J = 7.25 Hz, 4H, Ar CH) ppm.

$^{31}P$ NMR (104.2 MHz, $C_6D_6$, 300 K): δ - 48.4 (s) ppm.

$^{13}C\{^{1}H\}$ NMR (151 MHz, $C_6D_6$, 300 K): δ 28.4 (br. s, *i*Bu $CH_2$); 28.5 (s, *i*Bu CH); 29.8 (s, *i*Bu $CH_3$); 66.3 (s, 12-crown-4 $CH_2$); 124.7 (s, Ar CH); 127.7 (d, J = 6.69 Hz, Ar CH); 134.3 (d, J = 12.84 Hz, Ar CH); 144.7 (d, J = 12.57 Hz, ipso C) ppm.

$^{7}Li$ NMR (155.5 MHz, $C_6D_6$, 300 K): δ -0.10 (br. s) ppm.

$^{27}Al$ NMR: no signal was observed.

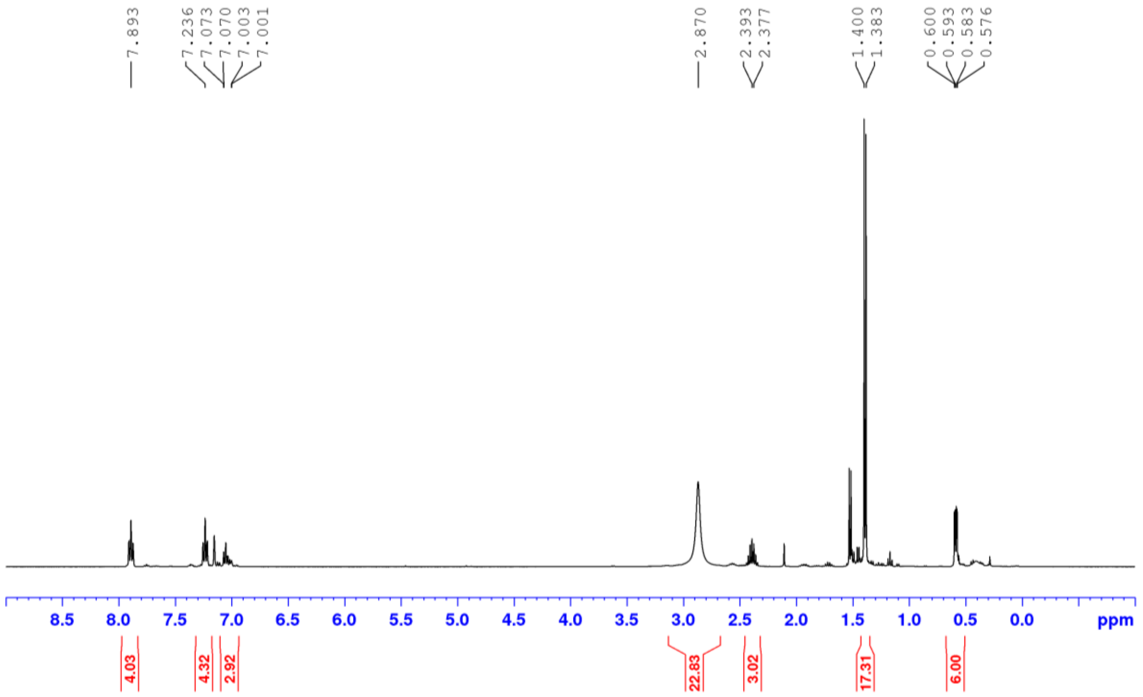


**Figure S9:** $^{1}H$ NMR spectrum of $iBu_3AlPPh_2Li$(12-crown-4), in $C_6D_6$

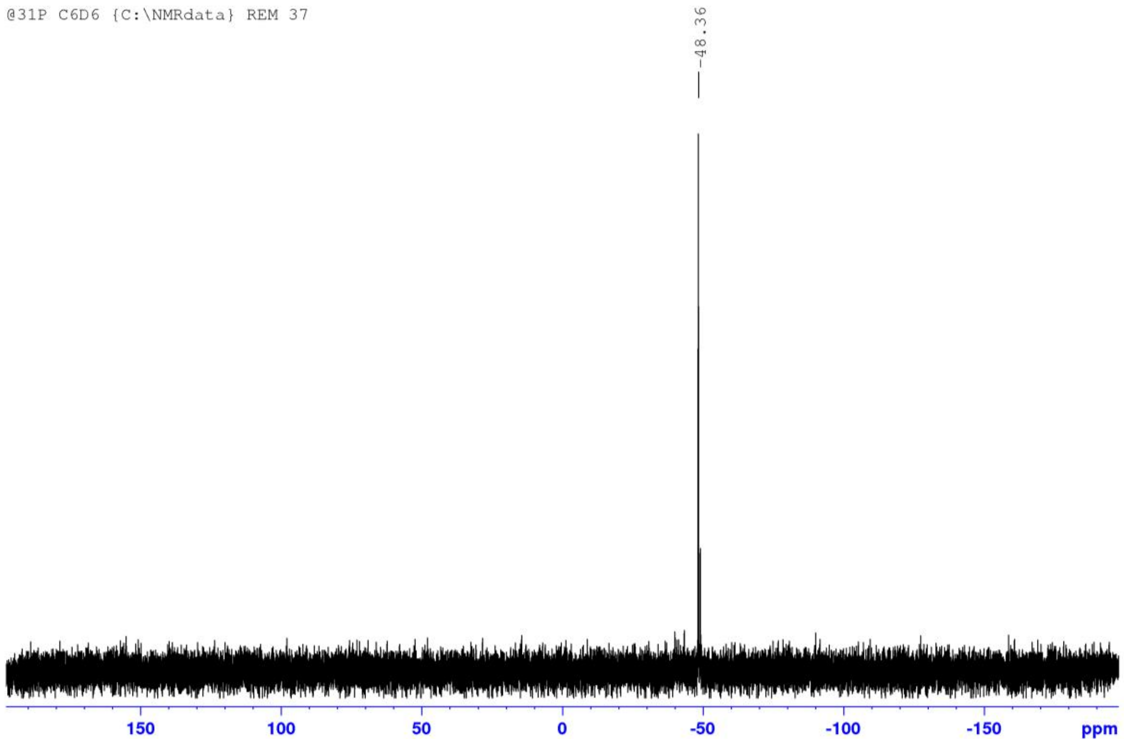


**Figure S10:** $^{31}P$ NMR spectrum of $iBu_3AlPPh_2Li$(12-crown-4), in $C_6D_6$

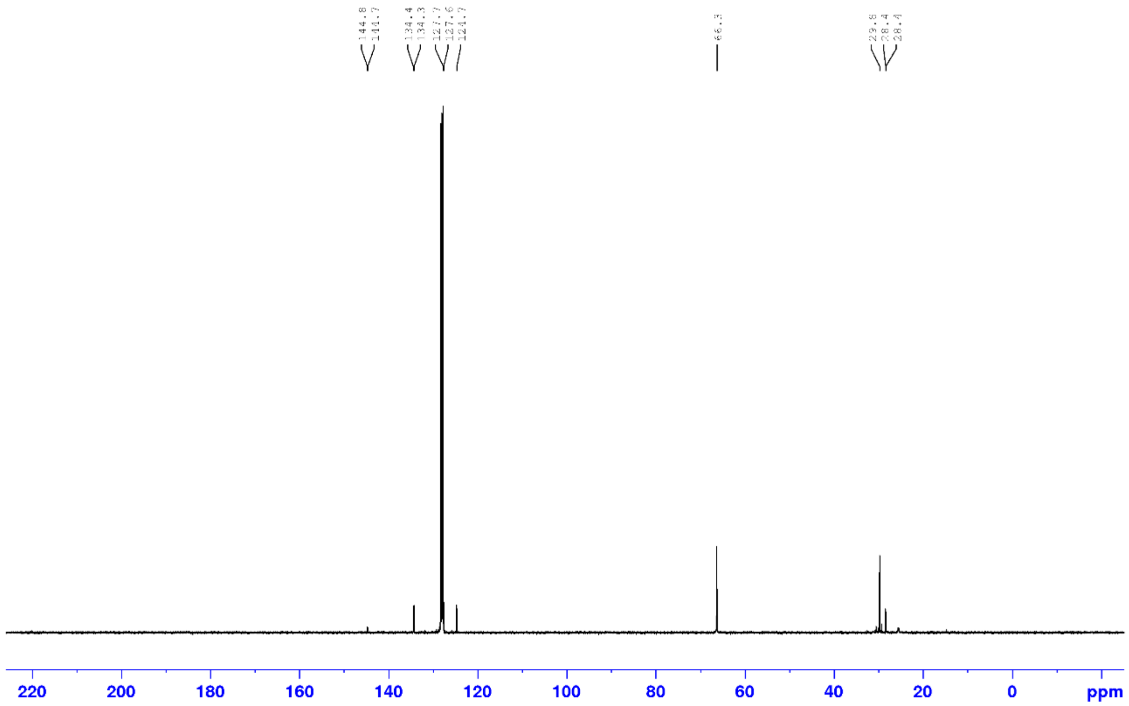


**Figure S11:** $^{13}C\{^1H\}$ NMR spectrum of $iBu_3AlPPh_2Li$(12-crown-4), in $C_6D_6$

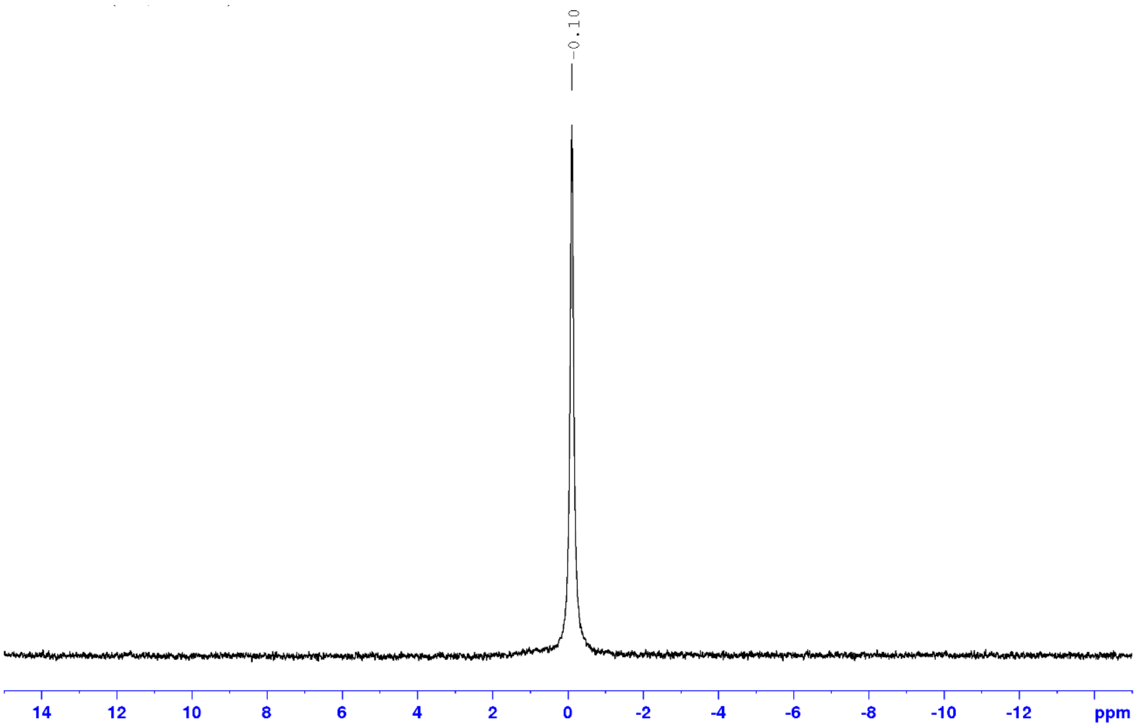


**Figure S12:** $^7$Li NMR spectrum of $iBu_3AlPPh_2Li$(12-crown-4), in $C_6D_6$

## Synthesis of $iBu_3AlHLi$(PMDETA)

To a solution of *i*$Bu_2AlH$ (2 mL; 1 M/hexane; 2 mmol) in hexane (5 mL) was added *i*BuLi (1.18 mL; 1.7 M/hexane; 2 mmol), dropwise. The resulting white suspension was stirred at room temperature for 1 hour. Addition of PMDETA (0.42 mL; 2 mmol) yielded a slightly cloudy solution which was placed in the fridge at 5 °C. Colourless crystals of the desired product were obtained, which were suitable for X-ray diffraction (0.54 g; 1.4 mmol; 71% yield).

$^1$H NMR (400.1 MHz, $C_6D_6$, 300 K): δ 0.39 (d; J = 6.87 Hz, 6H1, *i*Bu $CH_2$); 1.44 (d, J = 6.43Hz, 18H, *i*Bu $CH_3$); 1.52 – 1.61 (m, 11H, PMDETA 4x$CH_2$ and 1x$CH_3$); 1.92 (s; 6H, PMDETA $CH_3$); 2.37 (sept., J = 6.43 Hz, *i*Bu CH) ppm.

$^{13}$C{$^1$H} NMR (100.6 MHz, $C_6D_6$, 300 K): δ 29.0 (s, *i*Bu $CH_2$); 29.6 (s, *i*Bu CH); 44.4 (s, *i*Bu $CH_3$); 45.5 (br. s, PMDETA $CH_3$); 53.1 (s, PMDETA $CH_2$); 56.9 (s, PMDETA $CH_2$) ppm.

$^7$Li NMR (155.5 MHz, $C_6D_6$, 300 K): δ 0.56 (s) ppm.

No signal was observed in the $^{27}$Al NMR spectrum.

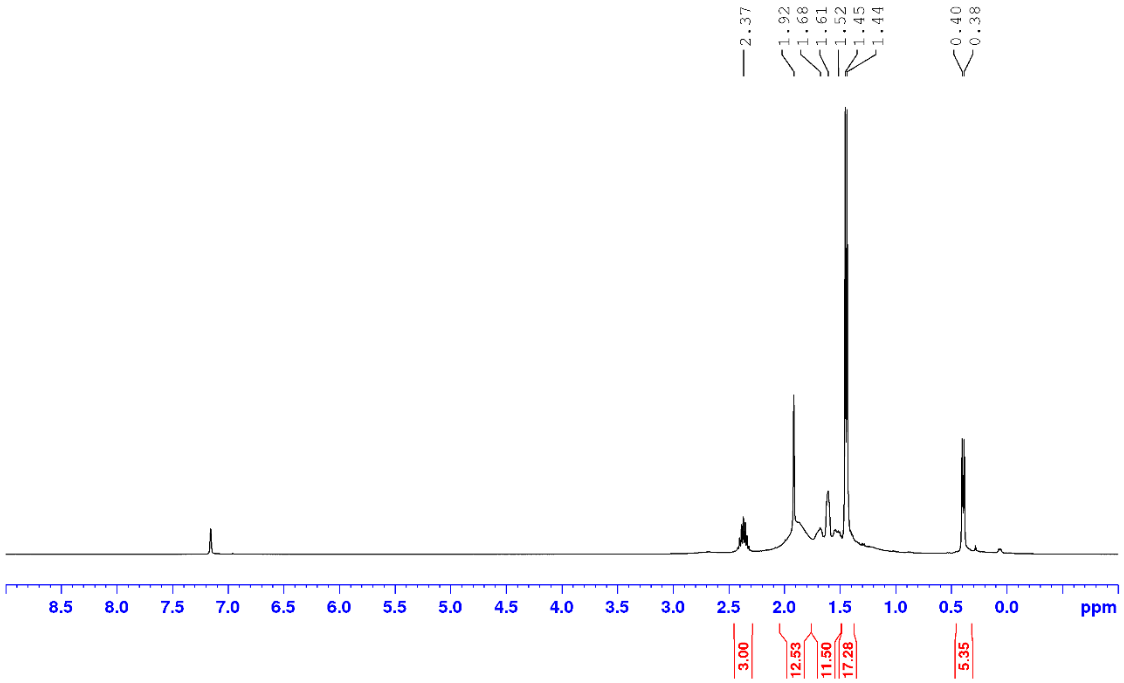


**Figure S13:** $^{1}$H NMR spectrum of $iBu_3AlHLi(PMDETA)$, in $C_6D_6$

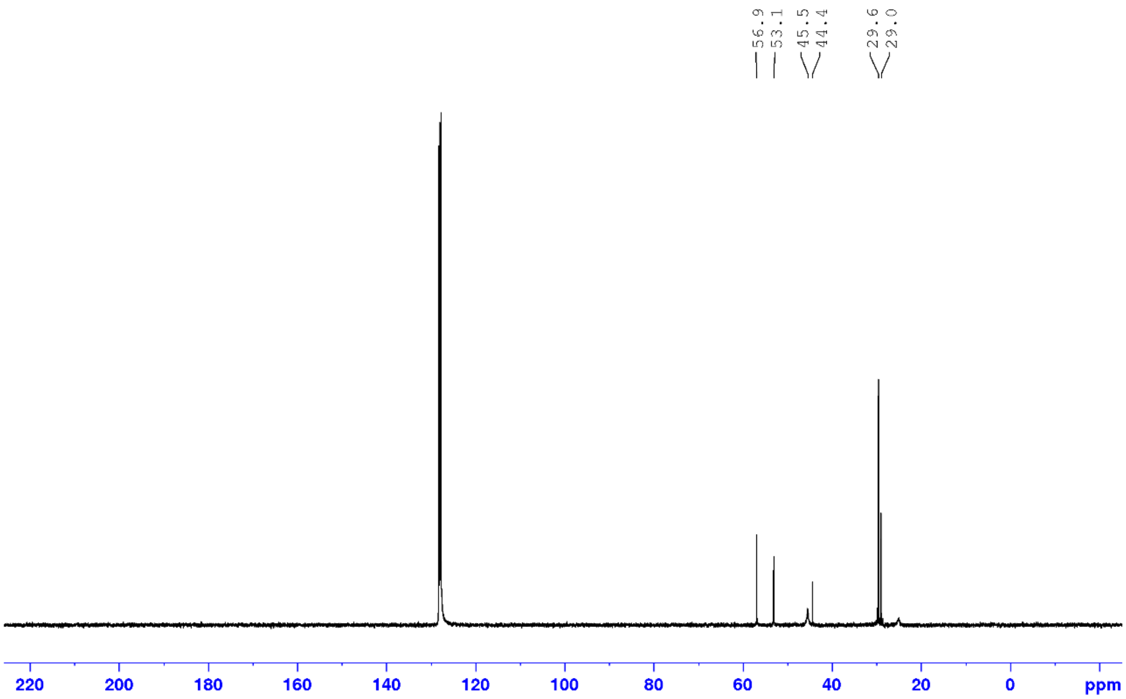


**Figure S14:** $^{13}C\{^1H\}$ NMR spectrum of $iBu_3AlHLi(PMDETA)$, in $C_6D_6$

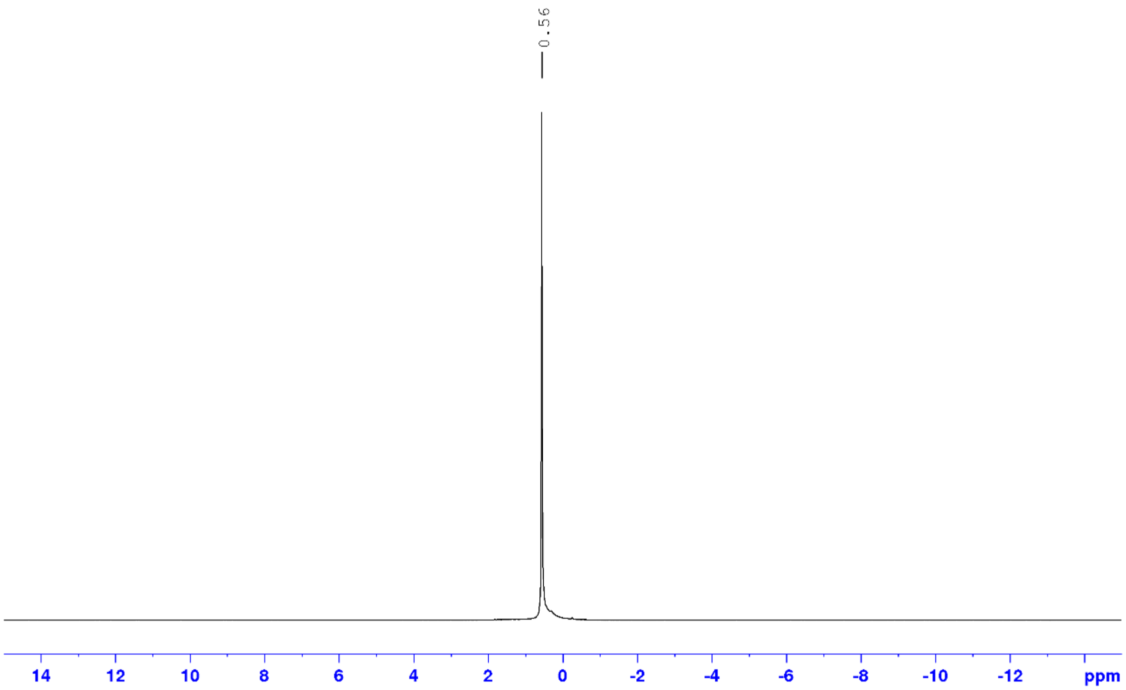


**Figure S15:** $^7Li$ NMR spectrum of $iBu_3AlHLi(PMDETA)$, in $C_6D_6$

## Stoichiometric Reactions

### Reaction of [iBu$_3$AlHLi]$_2$ + HPPh$_2$

In a J. Young's NMR tube [iBu$_3$AlHLi]$_2$ (0.0516 g; 0.25 mmol) was added to d$_8$-toluene (0.5 mL) and then HPPh$_2$ (0.05 mL; 0.25 mmol) and THF (60 µL; 0.75 mmol) were added. The progress of the reaction was monitored by $^1$H and $^{31}$P NMR spectroscopy.

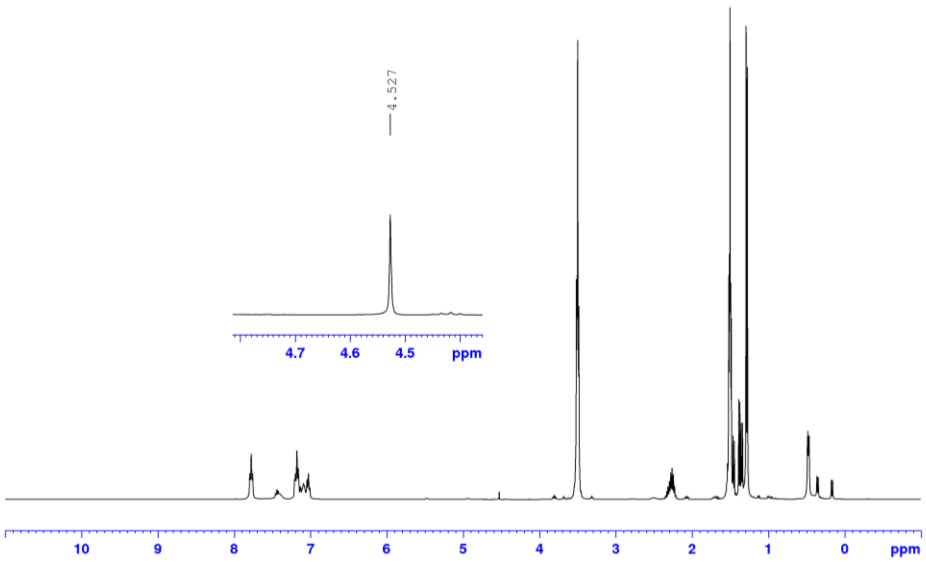


**Figure S16:** $^1$H NMR spectrum for the reaction of compound **1** + HPPh$_2$, after standing at room temperature overnight, showing formation of compound **2**, and $H_2$ in $C_6D_6$.

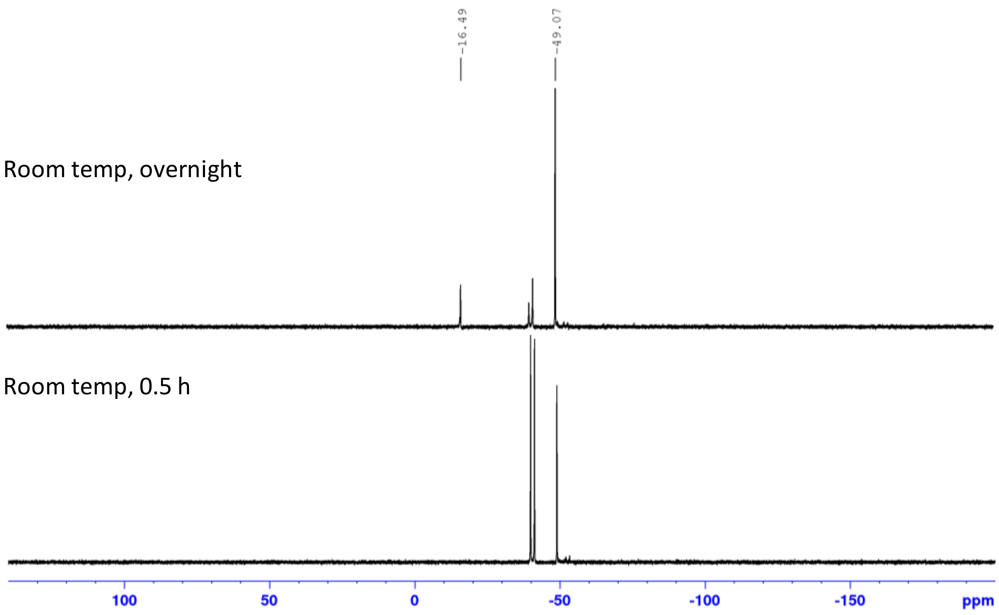


**Figure S17:** $^{31}$P NMR spectrum for the reaction of compound **1** + HPPh$_2$, showing formation of compound **2**, in $C_6D_6$. Signal at -16.5 is as a result of Ph$_2$P-PPh$_2$ dehydrocoupling product.

### Reaction of $[iBu_3AlHLi]_2$ + $DPPh_2$

In a J. Young's NMR tube $[iBu_3AlHLi]_2$ (0.0516 g; 0.25 mmol) was added to $d_8$-toluene (0.5 mL) and then $DPPh_2$ (0.05 mL; 0.25 mmol) and THF (60 µL; 0.25 mmol) were added. The progress of the reaction was monitored by $^1H$ and $^{31}P$ NMR spectroscopy.

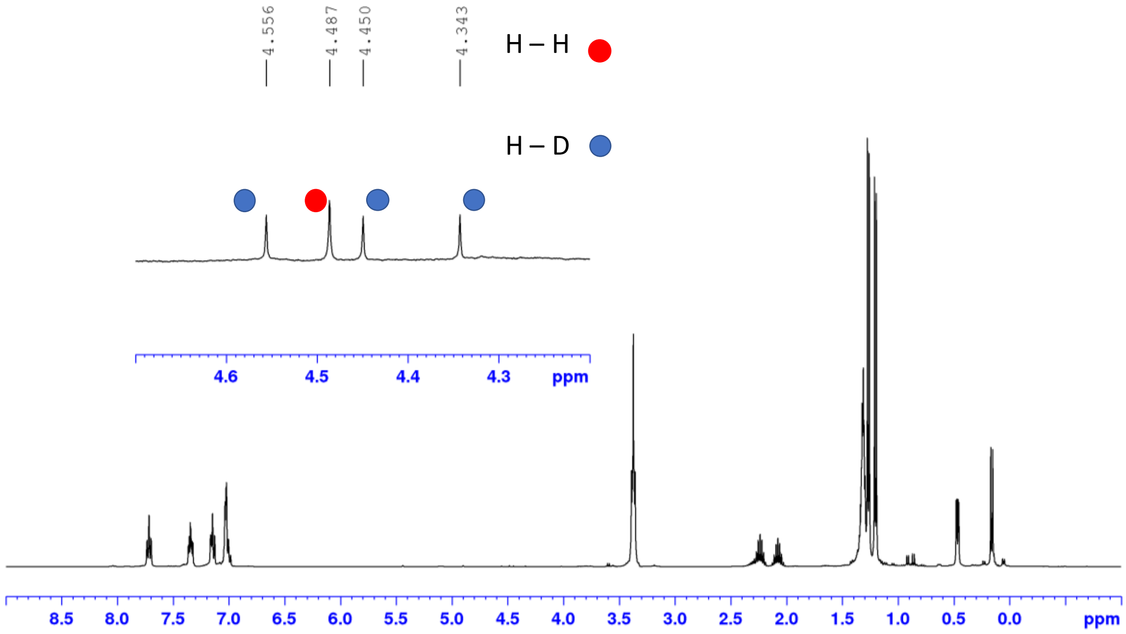


**Figure S18:** $^1H$ NMR spectrum for the reaction of compound **1** + $DPPh_2$, after standing at room temperature overnight, showing formation of compound **2**, $H_2$ and HD, in $C_6D_6$. Presence of $H_2$ is due to 90% deuterium incorporation of $DPPh_2$.

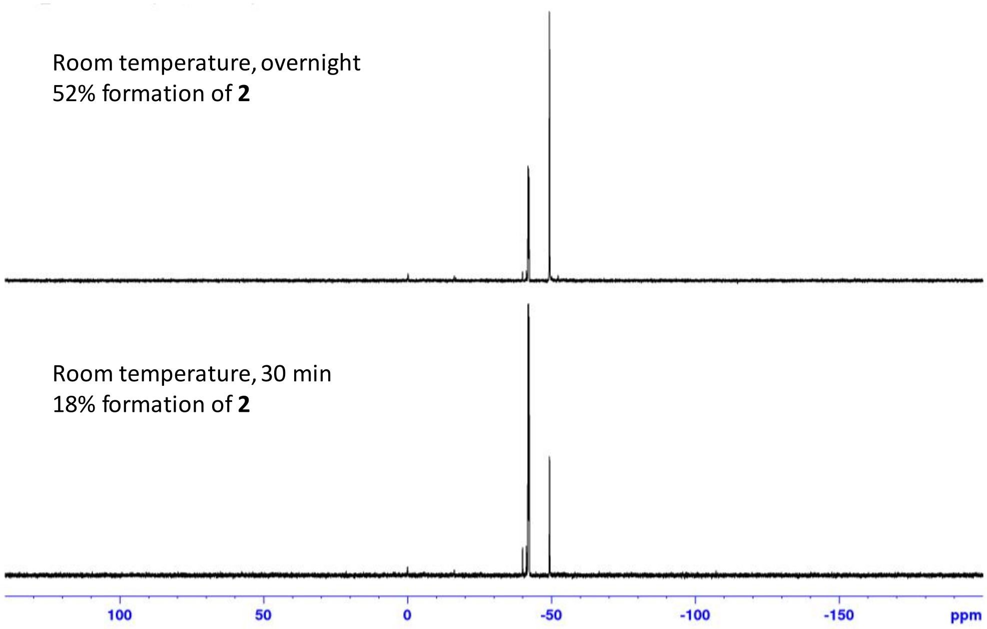


**Figure S19:** $^{31}$P NMR spectrum for the reaction of compound **1** + $DPPh_2$, showing formation of compound **2**, in $C_6D_6$

## Reaction of [$iBu_3AlPPh_2Li(THF)_3$ + diphenyl acetylene] + $HPPh_2$

In a J. Young's NMR tube $iBu_3AlPPh_2Li(THF)_3$ (0.0302 g; 0.05 mmol) was added to $d_8$-toluene (0.5 mL) and then diphenyl acetylene (0.0089 g; 0.05 mmol) was added. The solution was heated to 110 °C for 2 h, and then the $^1H$ and $^{31}P$ NMR spectra recorded. Excess $HPPh_2$ was then added and the reaction heated to 110 °C and monitored by $^1H$ and $^{31}P$ NMR spectroscopy.

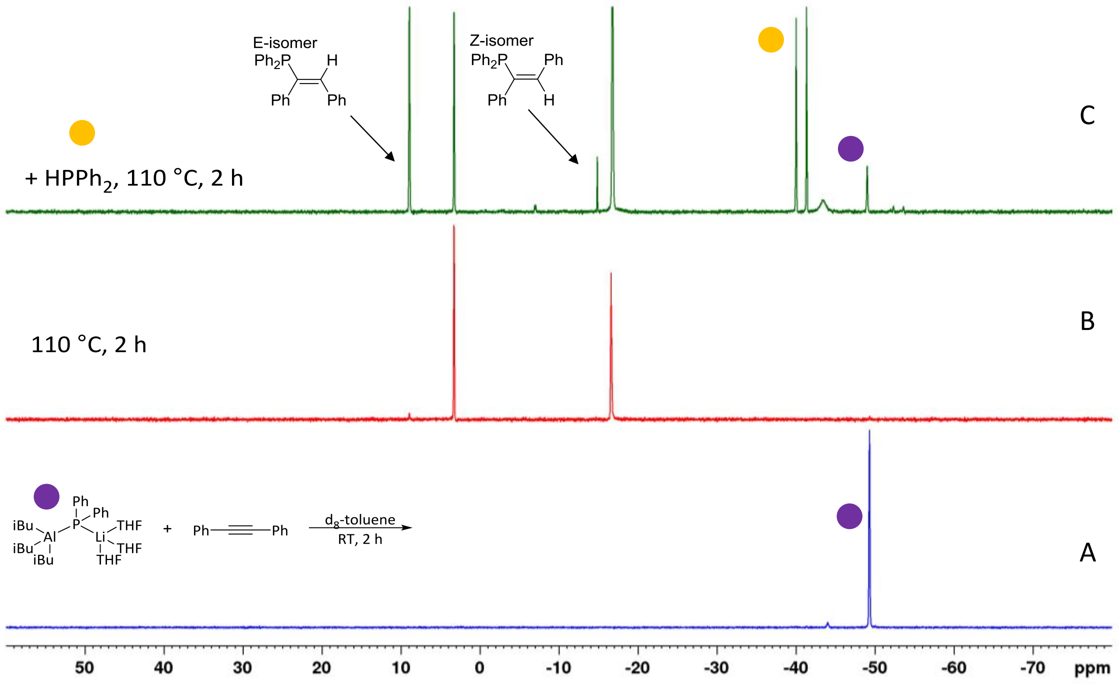


**Figure S20:** $^{31}P$ NMR spectra A) addition of diphenyl phosphine to **2** at room temperature; B) after heating for 2 h at 110 °C showing formation of two new signals; C) addition of excess $HPPh_2$ and subsequent heating at 110 °C generates some of vinyl phosphine products and regenerates **2**.

## Hydrophosphination Catalysis

### General Catalytic Reaction:

The desired catalyst loading was added to 0.5 mL of $d_8$-toluene solution (unless alternative solvent specified) containing the substrate precursor (0.6 mmol) and $HPPh_2$ (0.5 mmol, 0.09 mL). The reaction mixture was transferred to a sealed J. Young's tap NMR tube and the reaction was regularly monitored by $^1H$ and $^{31}P$ until the formation of the products was completed as determined by integration versus an internal capillary standard (hexamethylcyclotrisiloxane). The yields reported are based on $^1H$ NMR and $^{31}P$ relative to the internal standard. In all cases, the bulk of the NMR solution can be attributed to either product compounds or starting materials.

Isolated yields are provided for example substrates, isolated *via* either recrystallization methods or column chromatography, as reported.

For **alkynes** and **alkenes,** the hydrophosphination catalysis was performed at 110 °C with 10 mol% [Al] catalyst loading.

For **carbodiimides** the hydrophosphination catalysis was performed at room temperature with 5 mol% [Al] catalyst loading.

## Heating $HPPh_2$ in the presence of 2

In a J. Young's tube, $iBu_3AlPPh_2Li(THF)_3$ (0.0302 g; 0.05 mmol; 10 mol%) was added to a solution of $HPPh_2$ (0.09 mL; 0.5 mmol) in $d_8$-toluene. The NMR tube was subsequently heated for 20 h at 110 °C before being monitored for $Ph_2P$-$PPh_2$ formation by $^1H$ and $^{31}P$ NMR spectroscopy.

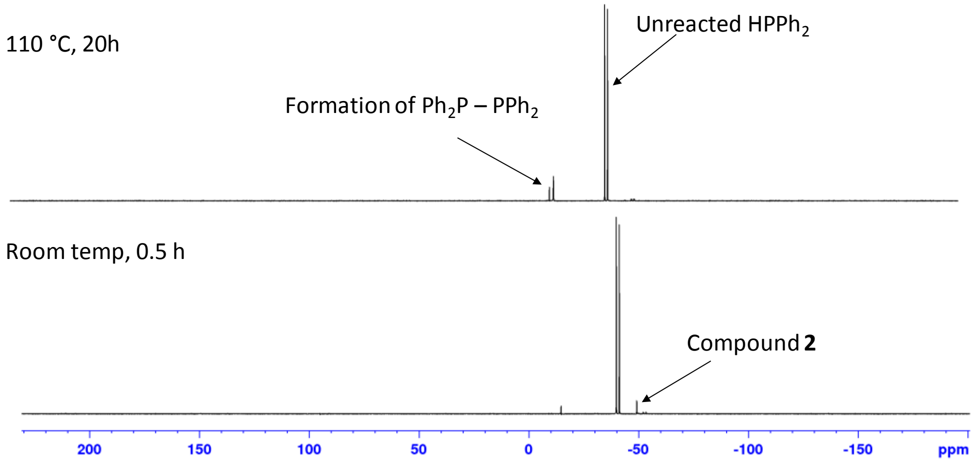


**Figure S21:** $^{31}P$ NMR spectrum for the heating of $HPPh_2$ at 110 °C in the presence of 10 mol% **2**, in $d_8$-toluene, showing only a trace amount of conversion after 20 h.

## Solvent Screen

### Phenylacetylene catalysed by **1**, in $d_8$-toluene

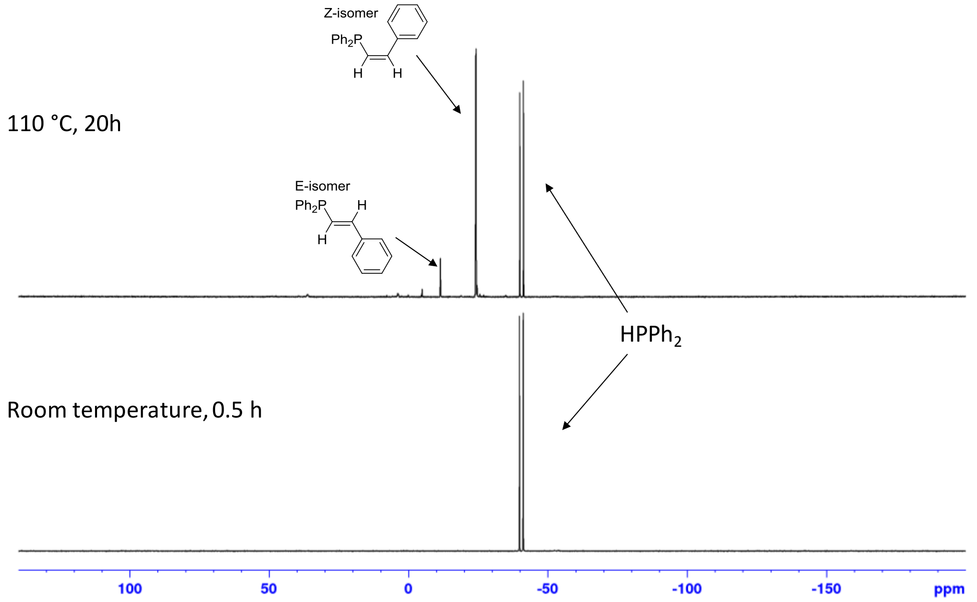


**Figure S22:** $^{31}P$ NMR spectrum for the hydrophosphination of phenylacetylene catalysed by **1** (5 mol% of dimer), in $d_8$-toluene at 110 °C

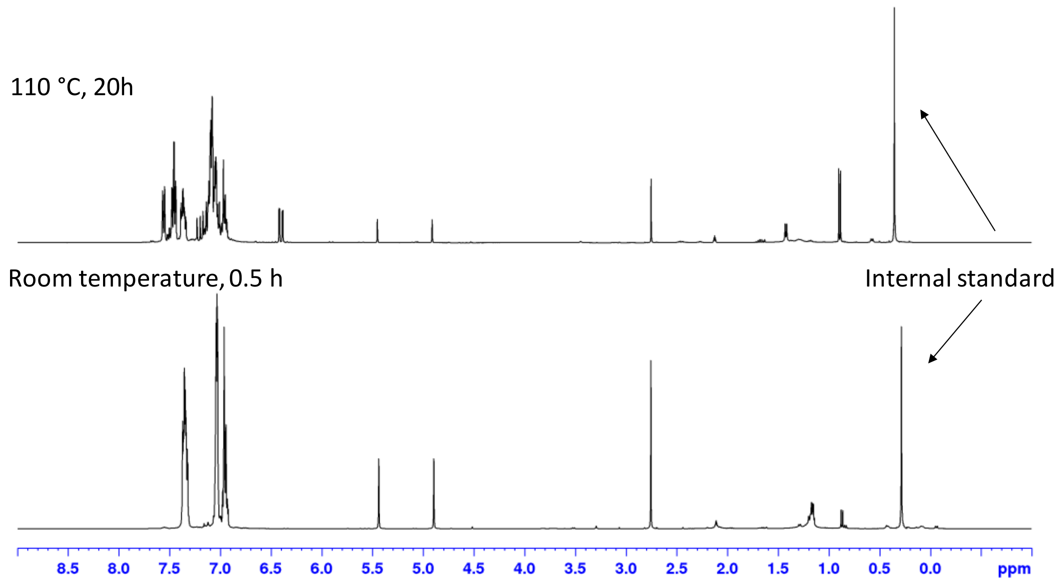


**Figure S23:** $^{1}H$ NMR spectrum for the hydrophosphination of phenylacetylene catalysed by **1** (5 mol% of dimer), in $d_8$-toluene at 110 °C.

## Phenylacetylene catalysed by **1**, in d8-THF

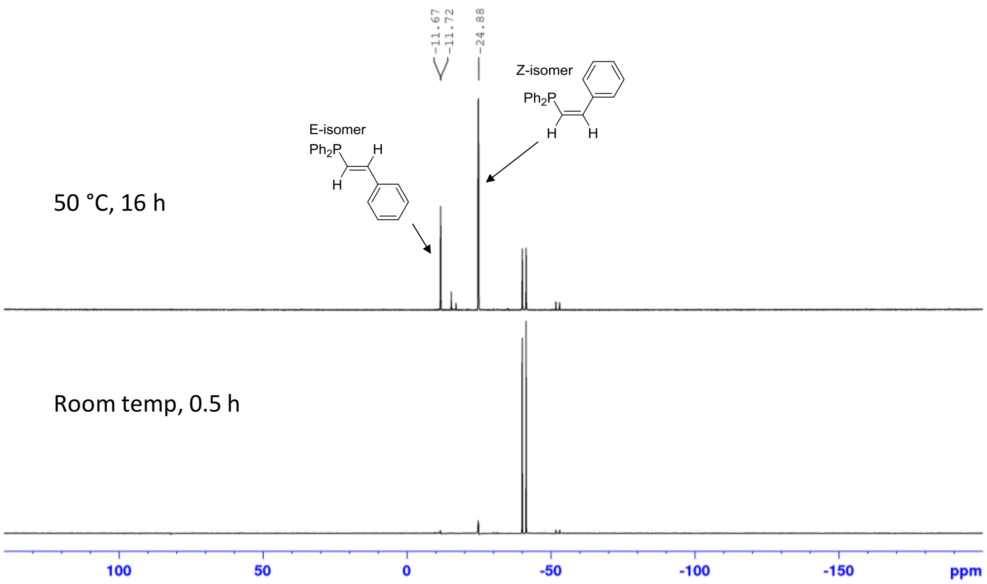


**Figure S24:** $^{31}$P NMR spectrum for the hydrophosphination of phenylacetylene catalysed by **1** (5 mol% of dimer), in d8-THF at 50 °C.

## Phenylacetylene catalysed by **1**, in $CD_2Cl_2$

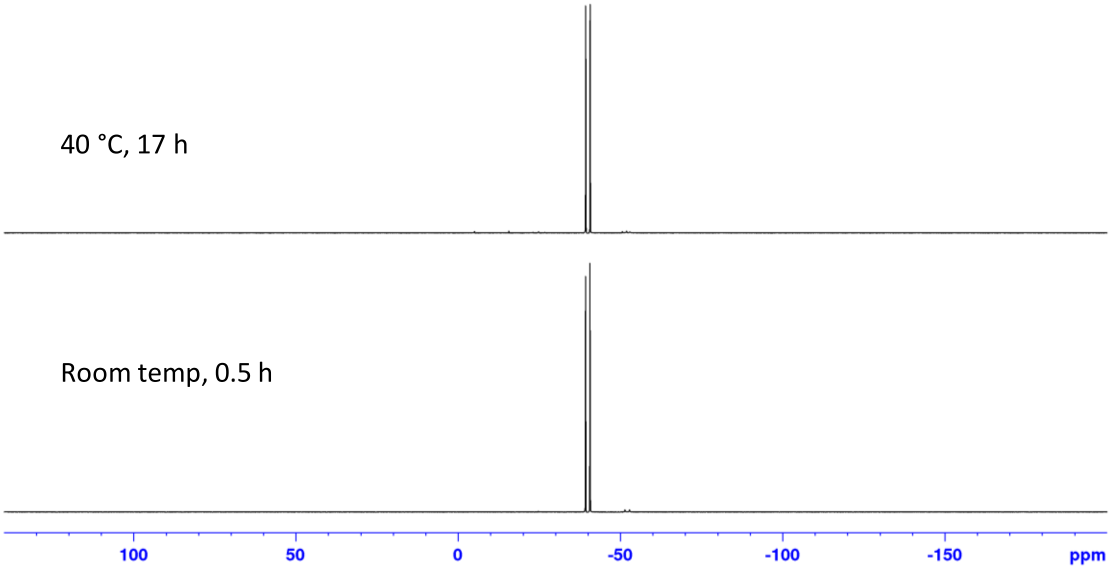


**Figure S25:** $^{31}$P NMR spectrum for the hydrophosphination of phenylacetylene catalysed by **1** (5 mol% of dimer), in $CD_2Cl_2$ at 40 °C.

## Phenylacetylene catalysed by **1**, in $C_6D_6$

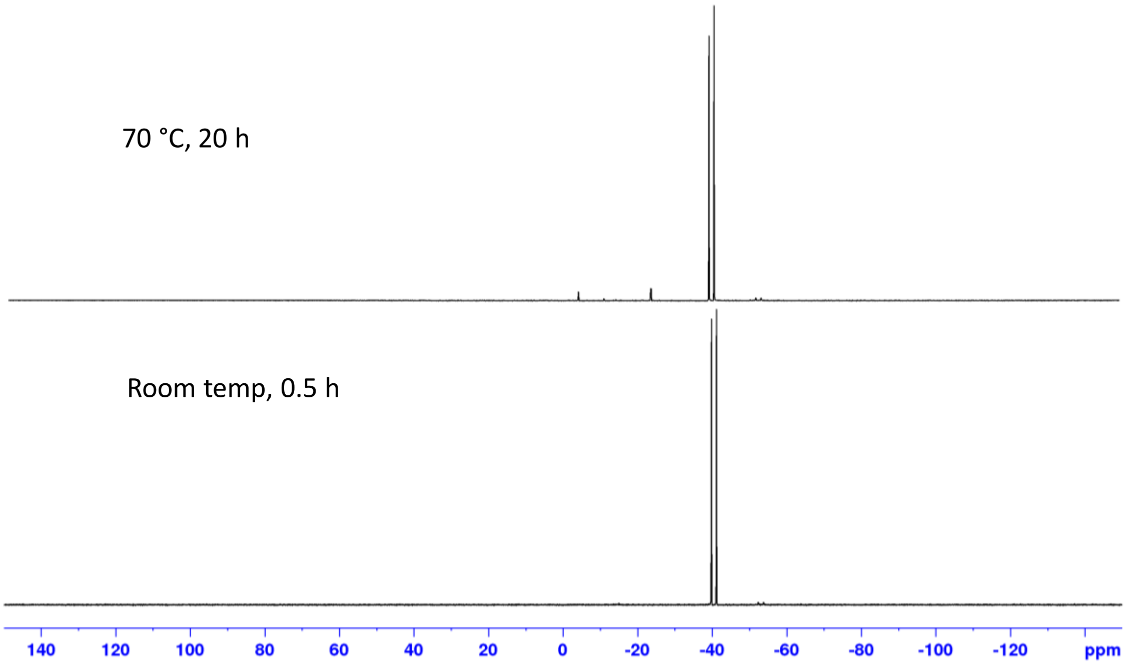


**Figure S26:** $^{31}P$ NMR spectrum for the hydrophosphination of phenylacetylene catalysed by **1** (5 mol% of dimer), in $C_6D_6$ at 70 °C.

# Hydrophosphination of alkynes

## Phenylacetylene catalysed by **2**

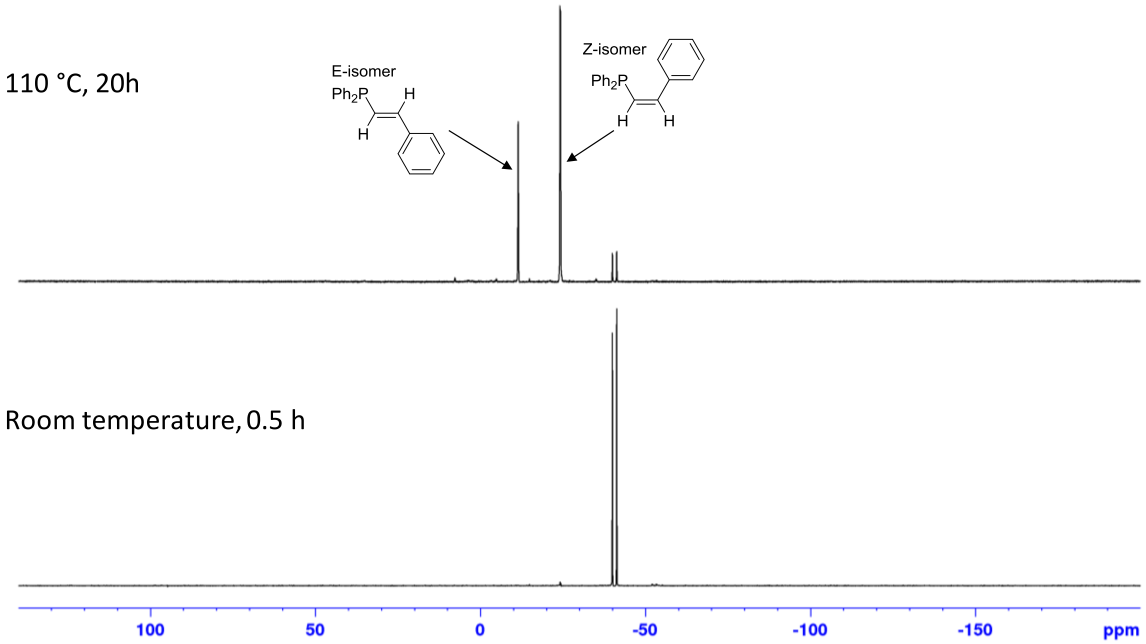


**Figure S27:** $^{31}P$ NMR spectrum for the hydrophosphination of phenylacetylene catalysed by **2** (10 mol%), in $d_8$-toluene

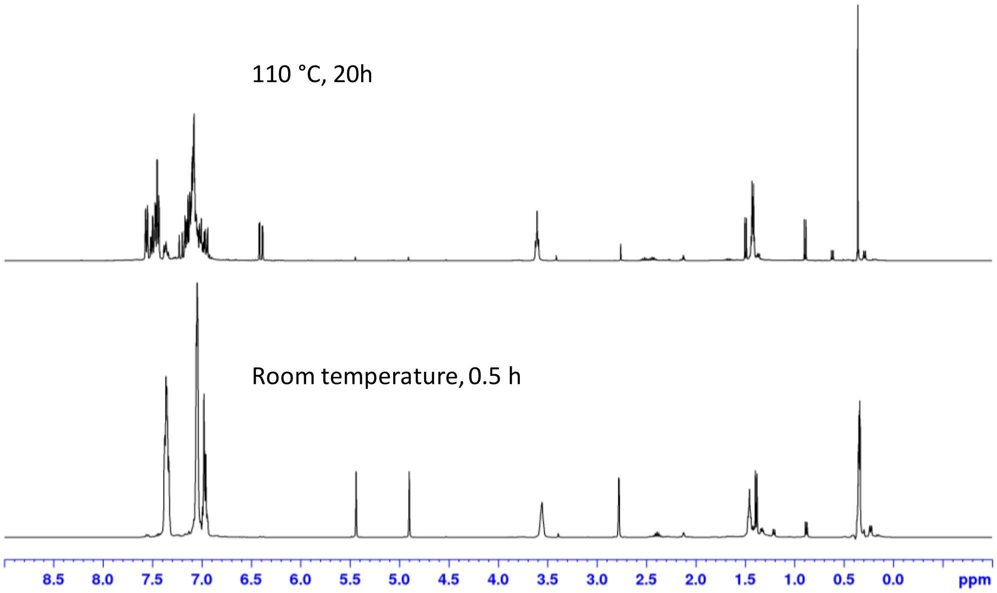


**Figure S28:** $^1$H NMR spectrum for the hydrophosphination of phenylacetylene catalysed by **2** (10 mol%), in $d_8$-toluene

The diphenyl(styryl)phosphine product was isolated by column chromatography (silica gel 1:19 EtOAc:hexane). Isolated yield 0.112 g; 0.39 mmol; 78 %.

The product was obtained as an intractable mixture of *E*- and *Z*-isomers (0.6:1 *E*/*Z*). Trace amounts of $HPPh_2$ and phosphine oxides were detected by NMR spectroscopy.

*E*-isomer: $^1$H NMR (600 MHz, $CDCl_3$, 300K): δ 6.95 (d, J= 10.80 Hz, 1H, *E*-isomer C(**H**)–$PPh_2$); 7.22 – 7.40 (m, 10H, Ar C–H); 7.4 – 7.58 (m, 6H, Ar C–H) ppm.

*Z*-isomer: $^1$H NMR (600 MHz, $CDCl_3$, 300K): δ 6.46 (dd, J = 2.83 Hz, 12.73 Hz, 1H, *Z*-isomer C(**H**)–$PPh_2$); 7.22 – 7.40 (m, 10H, Ar C–H); 7.4 – 7.58 (m, 6H, Ar C–H) ppm.

$^{31}$P NMR (243 MHz, $CDCl_3$, 300K): δ – 11.5 (*E*-isomer); – 24.7 (*Z*-isomer) ppm.

$^{13}$C NMR (151 MHz, $CDCl_3$, 300K): δ 119.3 (d, J = 103.7 Hz, Ar C–H); 126.9 (s, Ar C–H); 128.1 (s, Ar C–H); 128.5 (s, Ar C–H); 128.6 (d, J = 5.40 Hz, Ar C–H); 129.5 (d, J = 13.54 Hz *E*-isomer CH=**C**(H)P); 129.6 (d, J = 8.3 Hz, *Z*-isomer CH=**C**(H)P); 132.7 (d, J = 18.79 Hz, Ar C–H); 133.1 (d, J = 19.34 Hz, Ar C–H); 134.0 (d, J = 17.68 Hz, Ar C–H); 137.0 (d, J = 2.33 Hz, quat. C); 138.2 (d, J = 9.61 Hz, quat. C); 139.3 (d, J = 9.61 Hz, quat. C); 143.8 (d, J = 31.26 Hz, *E*-isomer **C**(H)=C(H)P); 144.1 (d, J = 18.90 Hz, *Z*-isomer **C**(H)=C(H)P) ppm.

m/z (GCMS EI): 287.1 $[M - H]^+$.

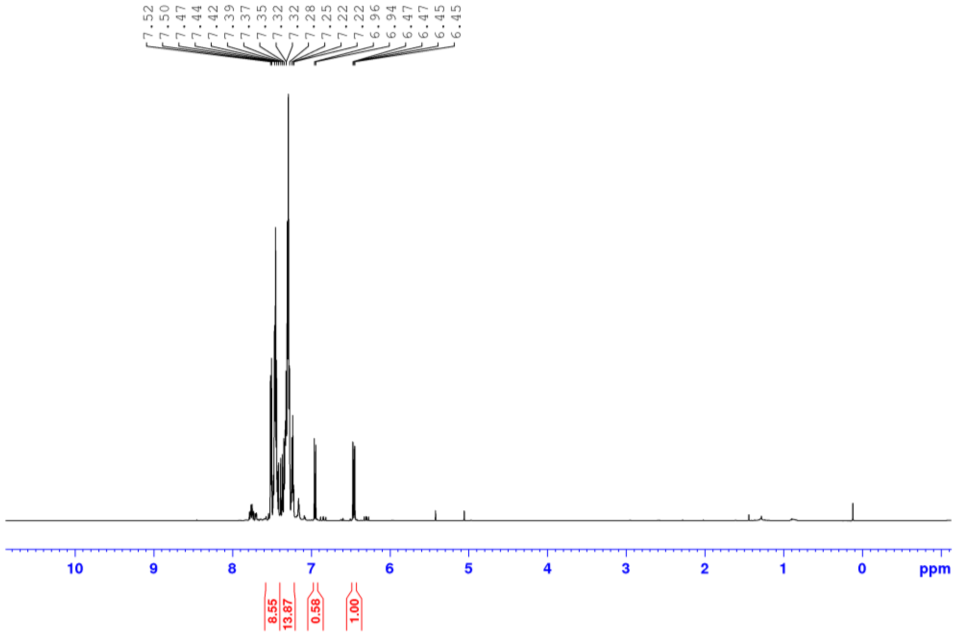


**Figure S29:** $^{1}$H NMR spectrum for isolated Ph(H)C=C(H)PPh$_2$, in $CDCl_3$

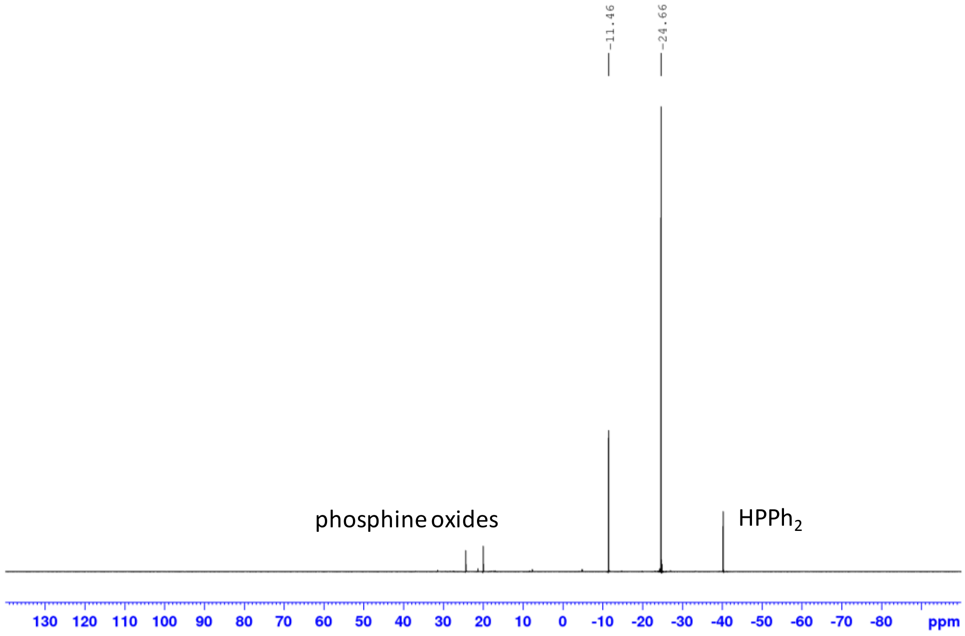


**Figure S30:** $^{31}$P NMR spectrum for isolated Ph(H)C=C(H)PPh$_2$, in $CDCl_3$

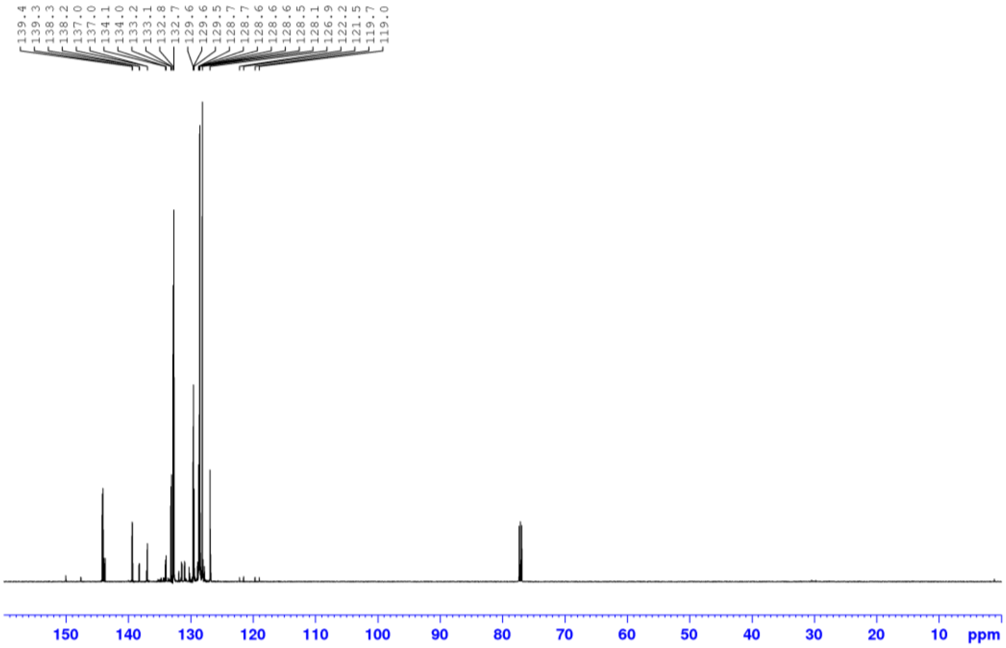

**Figure S31:** $^{13}C$ NMR spectrum for isolated Ph(H)C=C(H)PPh$_2$, in $CDCl_3$

## Phenylacetylene catalysed by 3

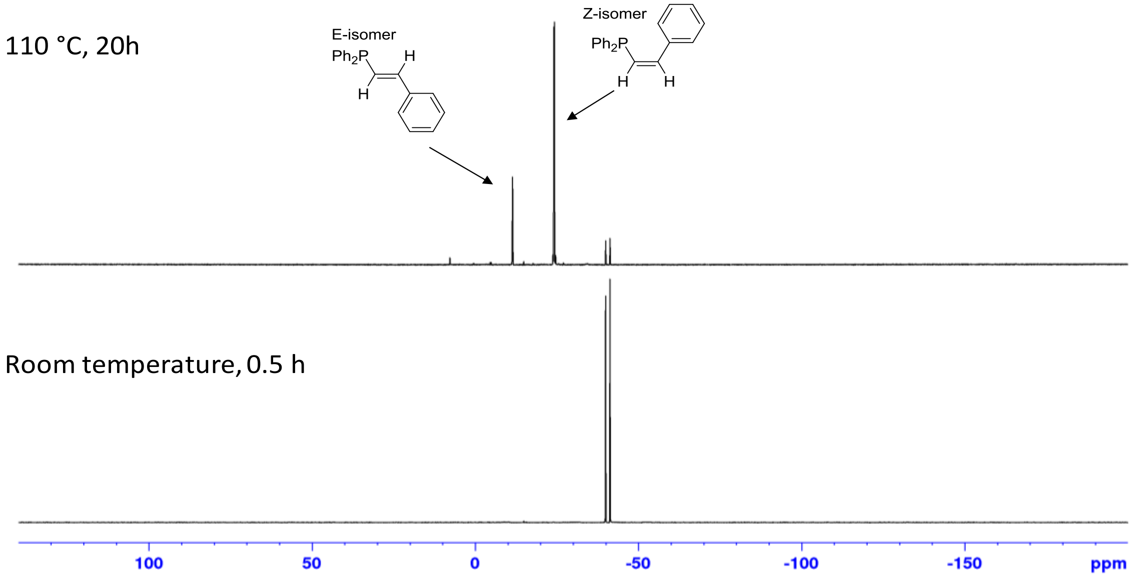


**Figure S32:** $^{31}P$ NMR spectrum for the hydrophosphination of phenylacetylene catalysed by **3** (10 mol%), in $d_8$-toluene

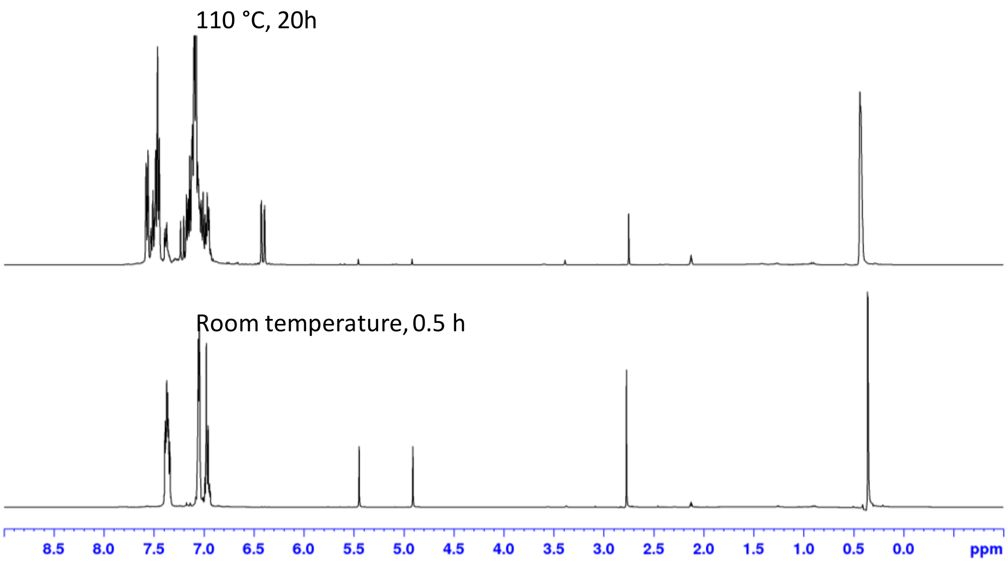


**Figure S33:** $^{1}$H NMR spectrum for the hydrophosphination of phenylacetylene catalysed by **3** (10 mol%), in $d_8$-toluene

## Phenylacetylene catalysed by 4

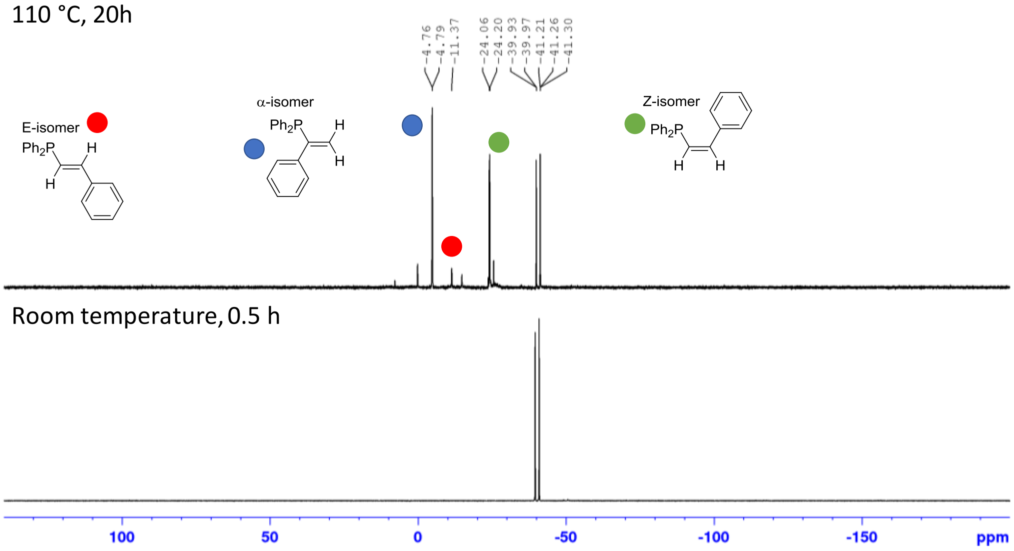


**Figure S34:** $^{31}$P NMR spectrum for the hydrophosphination of phenylacetylene catalysed by **4** (10 mol%), in $d_8$-toluene

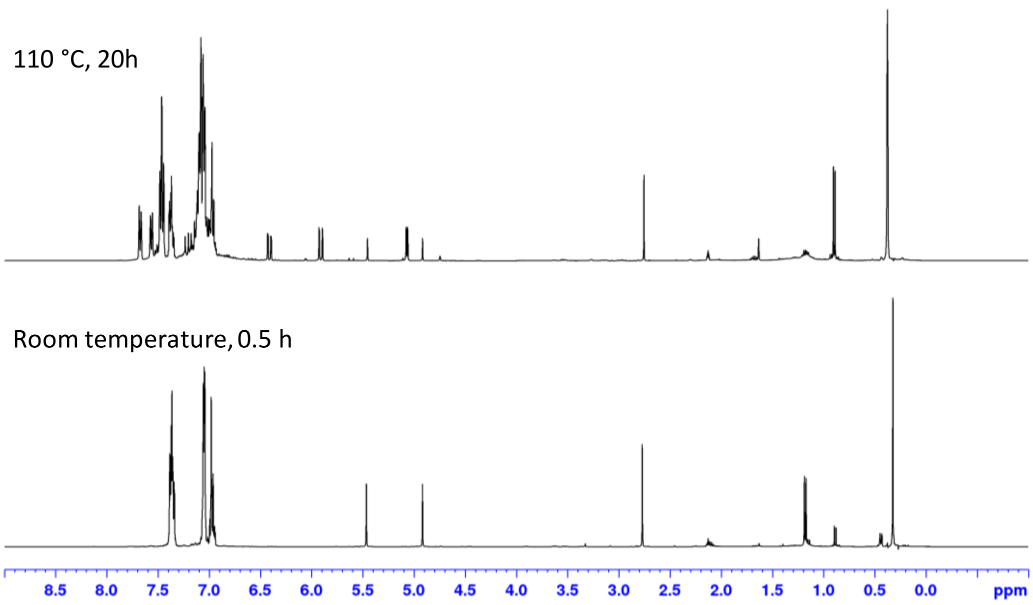


**Figure S35:** $^{1}H$ NMR spectrum for the hydrophosphination of phenylacetylene catalysed by **4** (10 mol%), in $d_8$-toluene

## Phenylacetylene catalysed by 5

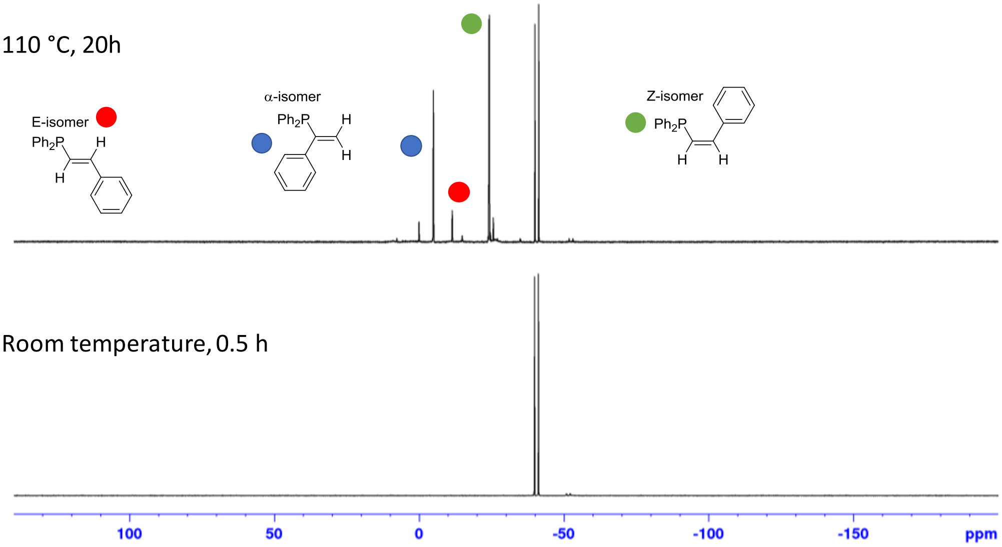


**Figure S36:** $^{31}P$ NMR spectrum for the hydrophosphination of phenylacetylene catalysed by **5** (10 mol%), in $d_8$-toluene

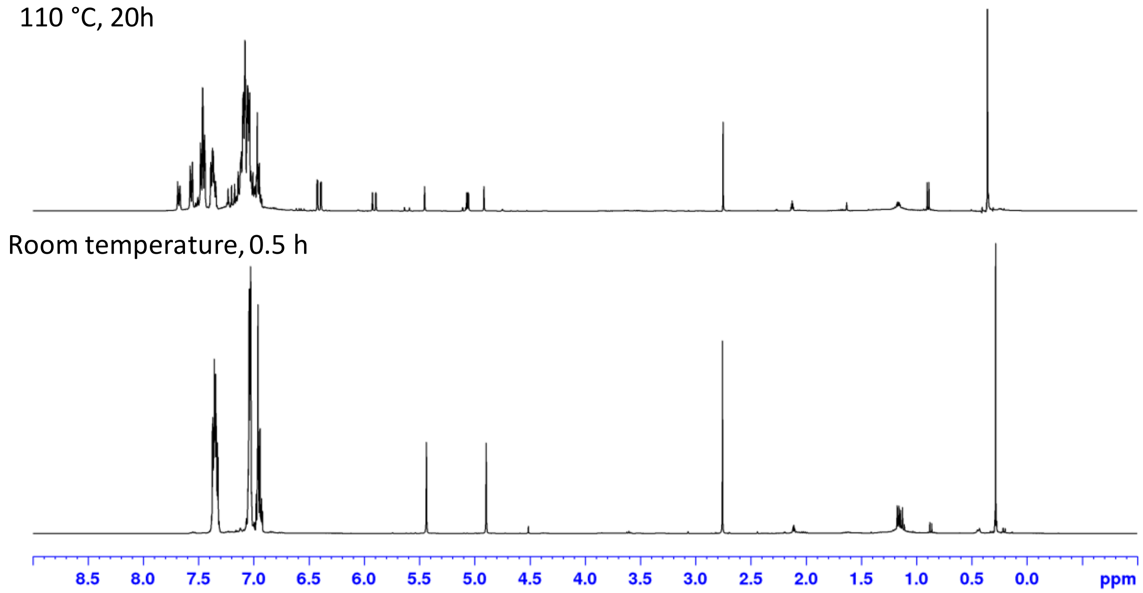


**Figure S37:** $^{1}$H NMR spectrum for the hydrophosphination of phenylacetylene catalysed by **5** (10 mol%), in $d_8$-toluene

## Phenylacetylene catalysed by **6**

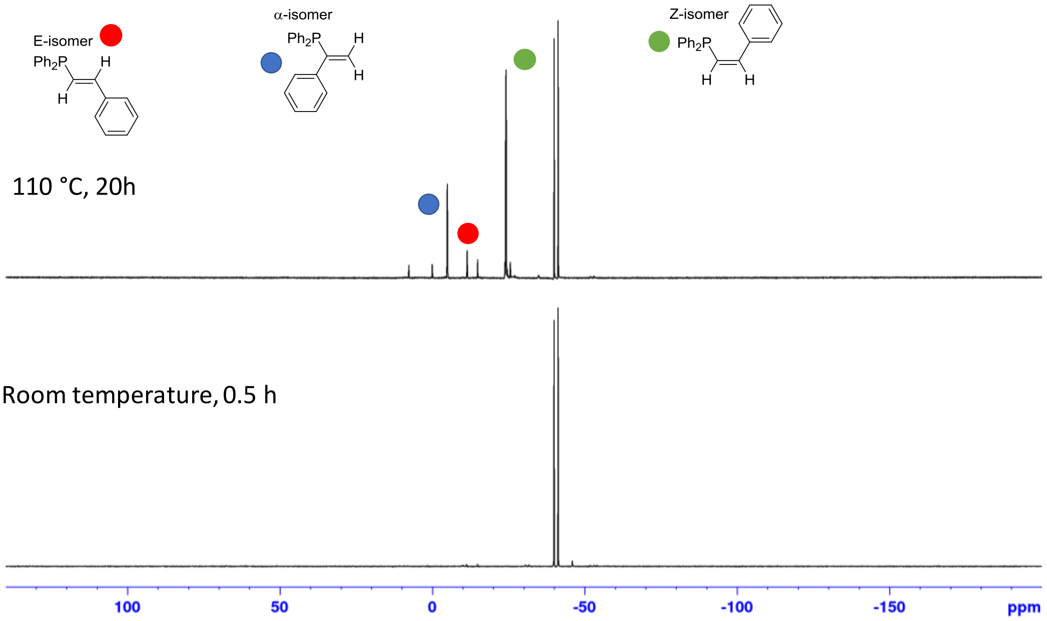


**Figure S38:** $^{31}$P NMR spectrum for the hydrophosphination of phenylacetylene catalysed by **6** (10 mol%), in $d_8$-toluene

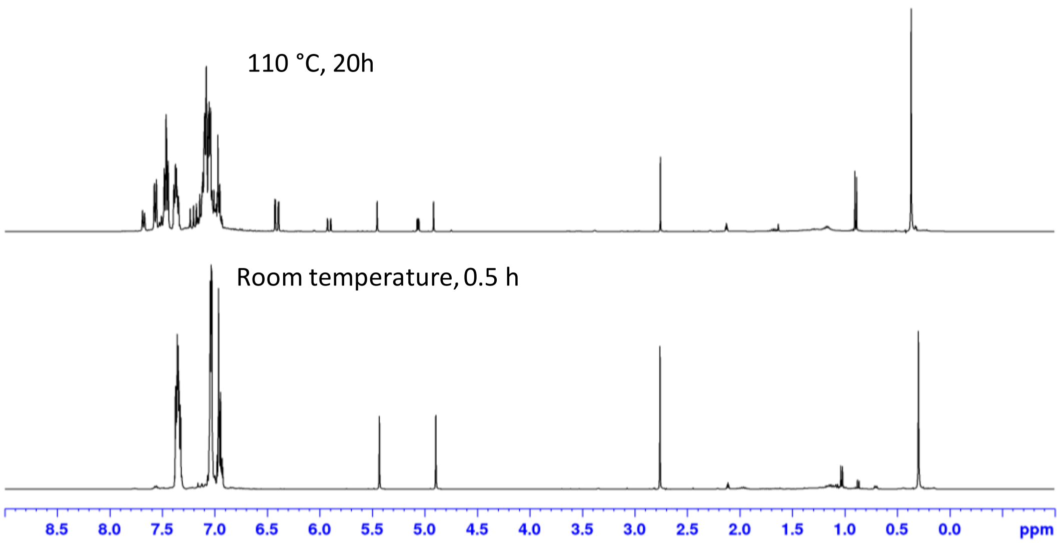


**Figure S39:** $^{1}$H NMR spectrum for the hydrophosphination of phenylacetylene catalysed by **6** (10 mol%), in $d_8$-toluene

## Diphenylacetylene catalysed by 1

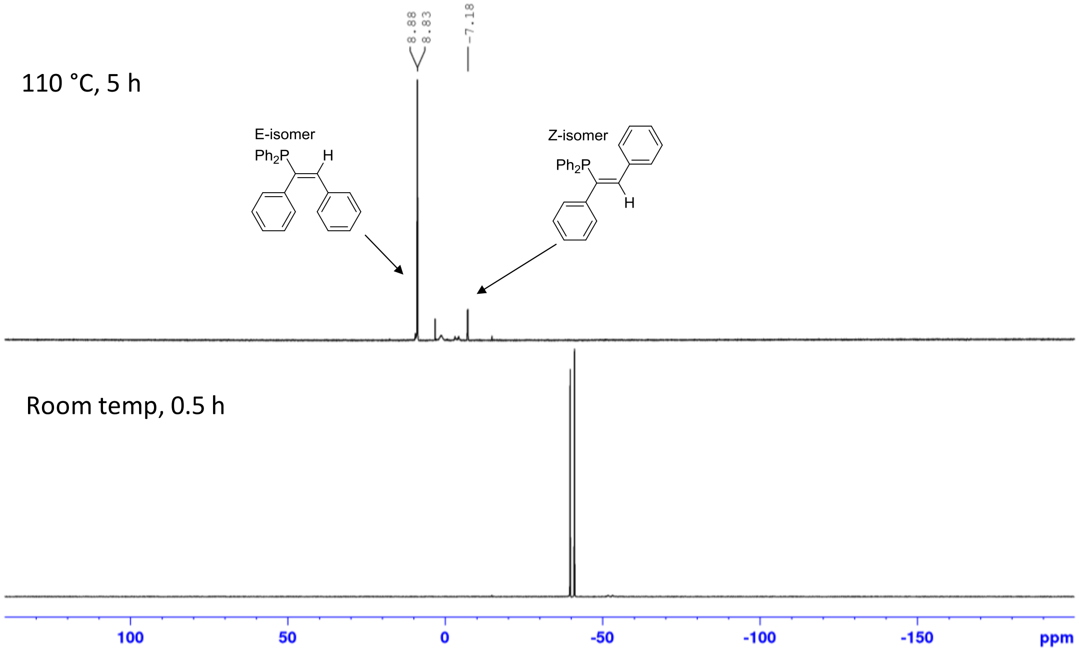


**Figure S40:** $^{31}$P NMR spectrum for the hydrophosphination of diphenylacetylene catalysed by **1** (5 mol% of dimer), in $d_8$-toluene

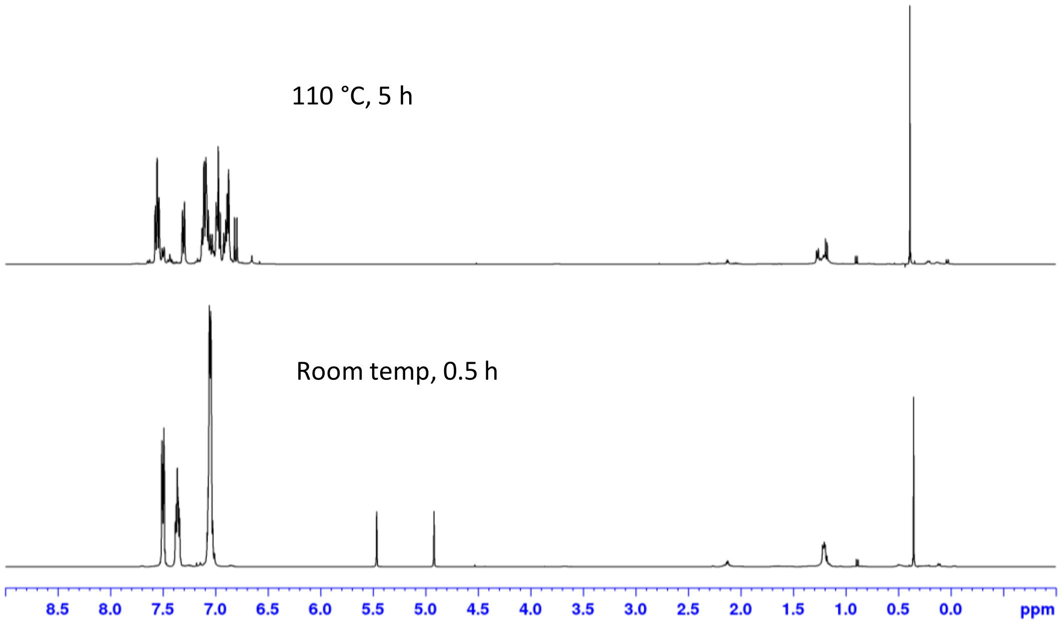


**Figure S41:** $^{1}H$ NMR spectrum for the hydrophosphination of diphenylacetylene catalysed by **1** (5 mol% of dimer), in $d_8$-toluene

## Diphenylacetylene catalysed by **1 + 30 mol% THF**

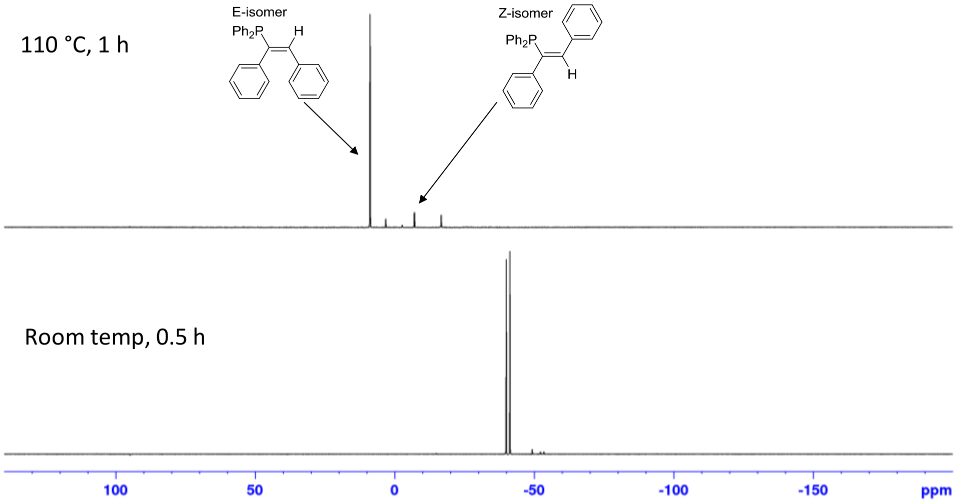


**Figure S42:** $^{31}P$ NMR spectrum for the hydrophosphination of diphenylacetylene catalysed by **1** (5 mol% of dimer), in the presence of **30 mol% THF**, in $d_8$-toluene

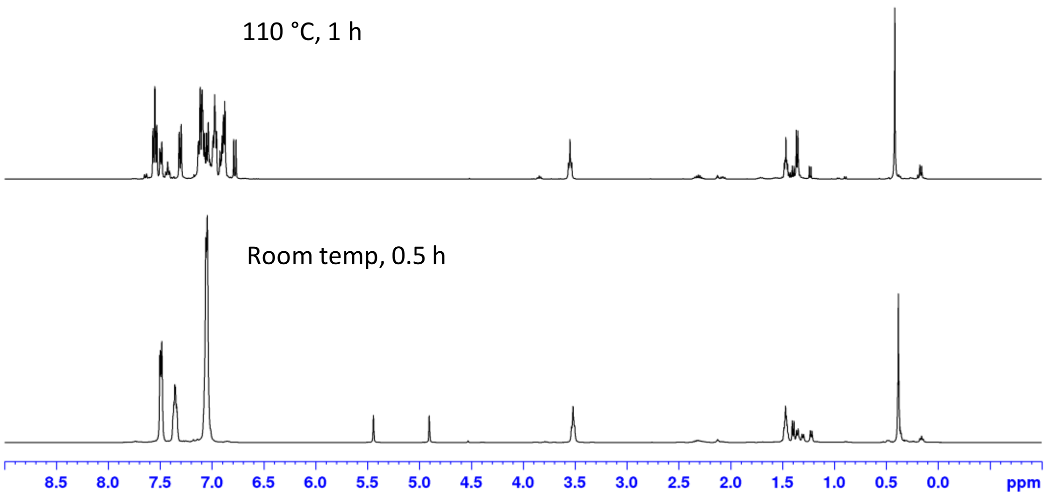


**Figure S43:** $^{1}H$ NMR spectrum for the hydrophosphination of diphenylacetylene catalysed by **1** (5 mol% of dimer), in the presence of **30 mol% THF**, in $d_8$-toluene

## Diphenylacetylene catalysed by **2**

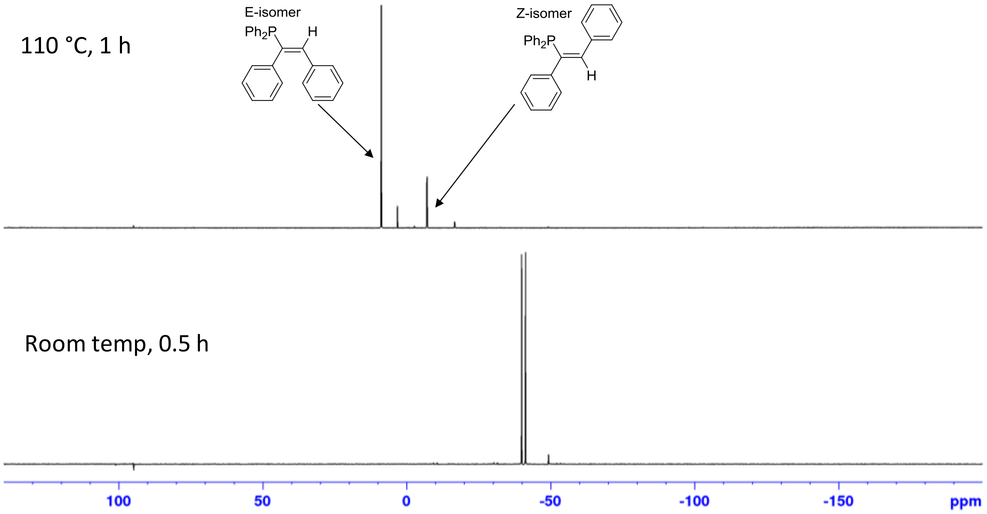


**Figure S44:** $^{31}P$ NMR spectrum for the hydrophosphination of diphenylacetylene catalysed by **2** (10 mol%), in $d_8$-toluene

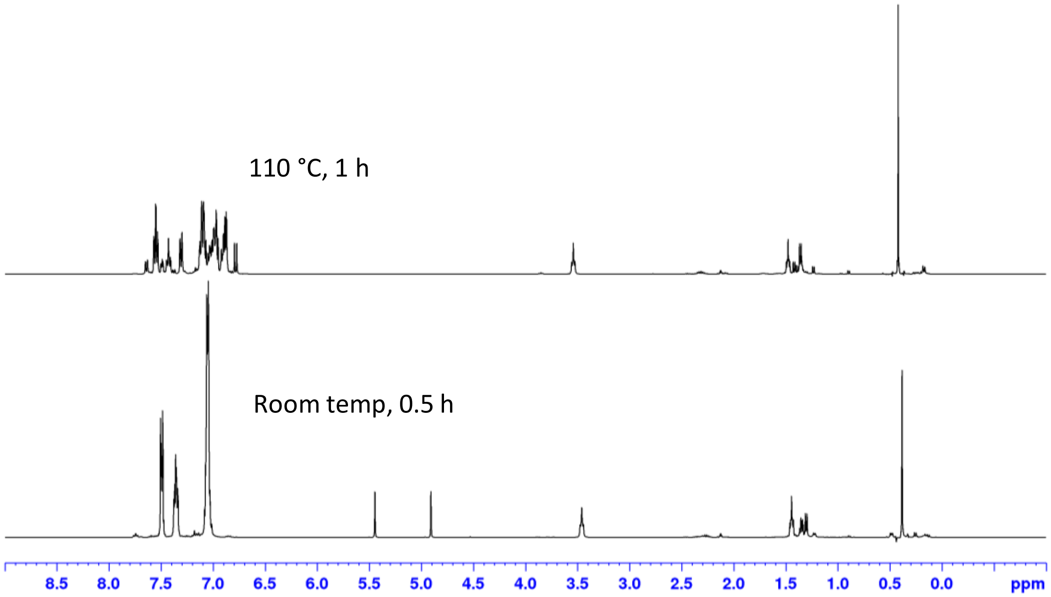


**Figure S45:** $^1$H NMR spectrum for the hydrophosphination of diphenylacetylene catalysed by **2** (10 mol%), in $d_8$-toluene

The (1,2-diphenylvinyl)diphenylphosphine product was isolated by filtering over a plug of silica and recrystalising from pentane. Isolated crystalline yield 0.146 g; 0.4 mmol; 80 %.

$^1$H NMR (400 MHz, $CDCl_3$, 300K): δ 6.54 (d, J= 9.29 Hz, 1H, C=C(H)); 6.93 – 6.95 (m, 2H, Ar); 7.09 – 7.10 (m, 3H, Ar); 7.16 – 7.19 (m, 5H, Ar); 7.33 – 7.36 (m, 6H, Ar); 7.46 – 7.50 (m, 4H, Ar) ppm.

$^{31}$P NMR (162.0 MHz, $CDCl_3$, 300K): δ 8.46 (s) ppm.

$^{13}$C NMR (100 MHz, $CDCl_3$, 300K): δ 127.1 (s, Ar C–H); 127.4 (s, Ar C–H); 128.1 (s, Ar C–H); 128.5 (s, Ar C–H); 128.6 (s, Ar C–H); 129.0 (s, Ar C–H); 129.2 (d, J = 6.17 Hz, Ar C–H); 129.4 (s, Ar C–H); 134.3 (s, J = 20.13 Hz, Ar C–H ); 135.5 (d, J = 12.57 Hz, quat. C); 137.0 (d, J = 6.77 Hz, quat. C); 138.1 (d, J = 18.86 Hz, **C**(H)=C); 140.1 (d, J = 17.02 Hz, quat. C); 141.5 (d, J = 18.91 Hz, quat. C) ppm.

m/z (GCMS EI): 363.3 $[M - H]^+$

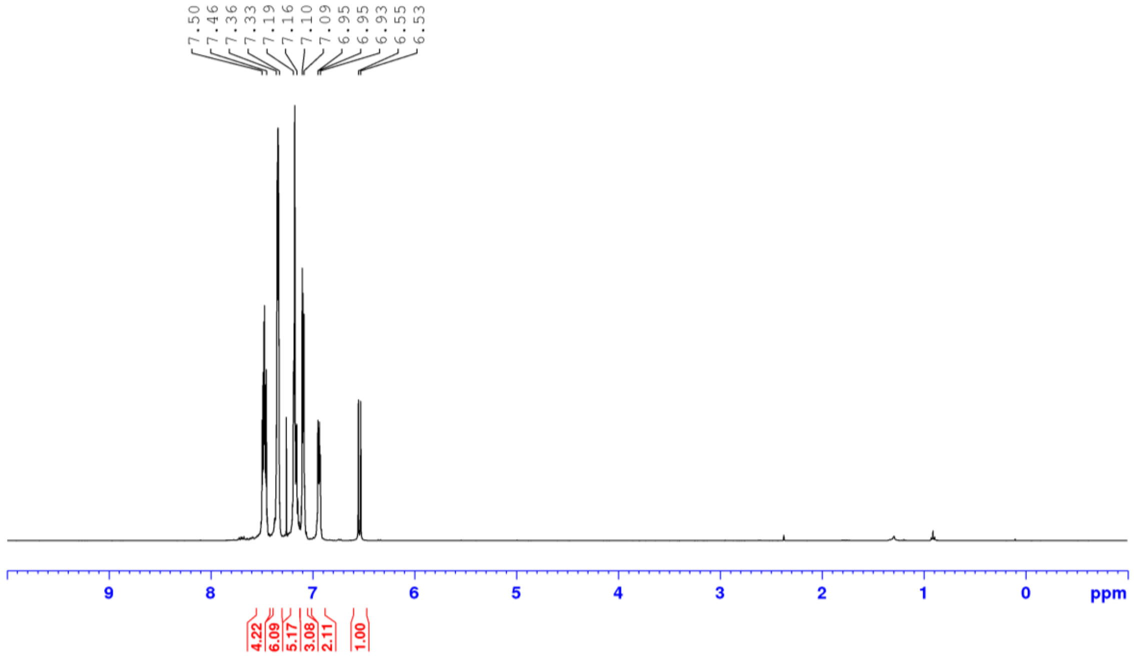


**Figure S46:** $^{1}H$ NMR spectrum for isolated Ph($PPh_2$)C=C(H)Ph, in $CDCl_3$

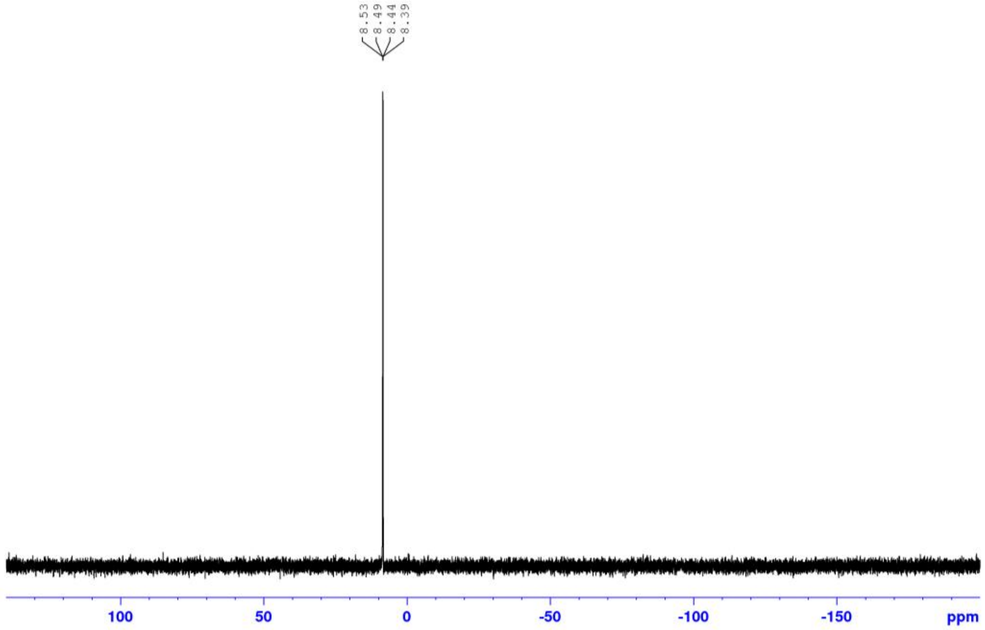


**Figure S47:** $^{31}P$ NMR spectrum for isolated Ph($PPh_2$)C=C(H)Ph, in $CDCl_3$

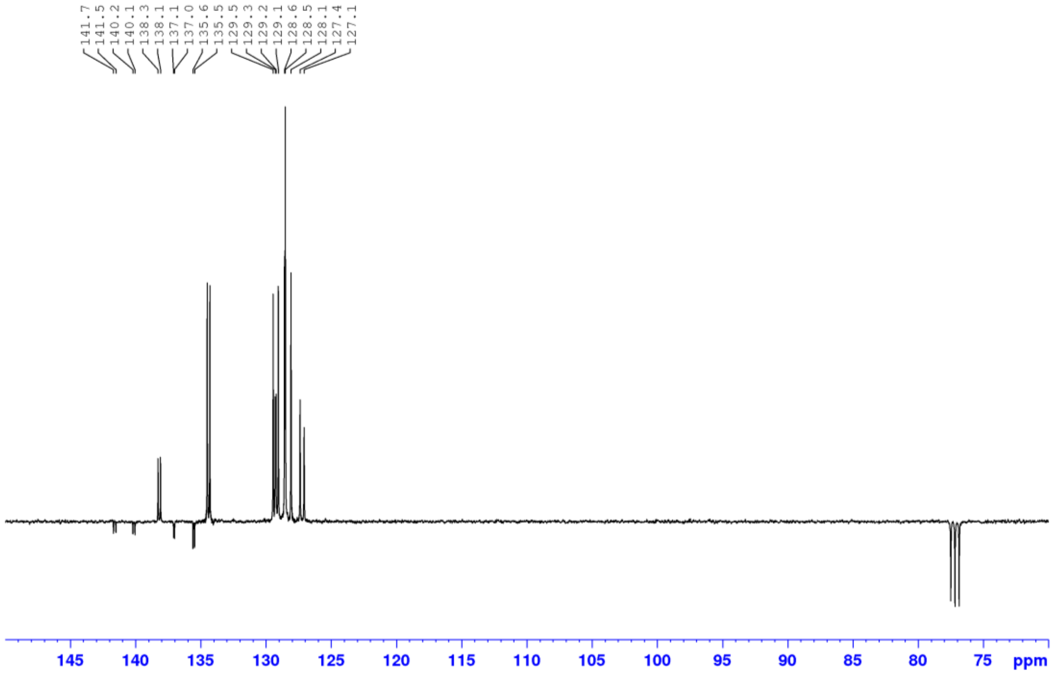


**Figure S48:** $^{13}C$ JMOD NMR spectrum for isolated $Ph(PPh_2)C{=}C(H)Ph$, in $CDCl_3$

## Diphenylacetylene catalysed by **3**

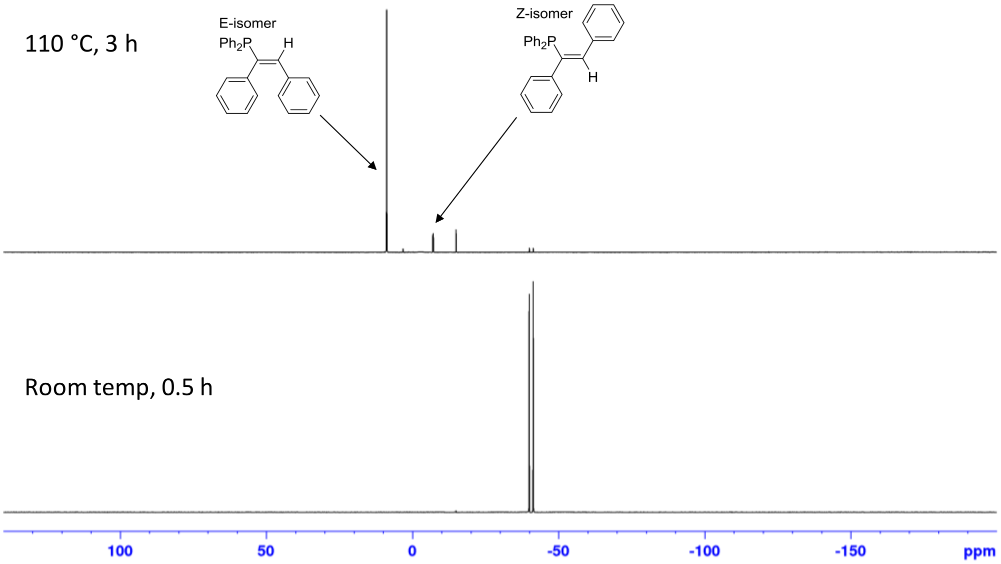


**Figure S49:** $^{31}P$ NMR spectrum for the hydrophosphination of diphenylacetylene catalysed by **3** (10 mol%), in $d_8$-toluene

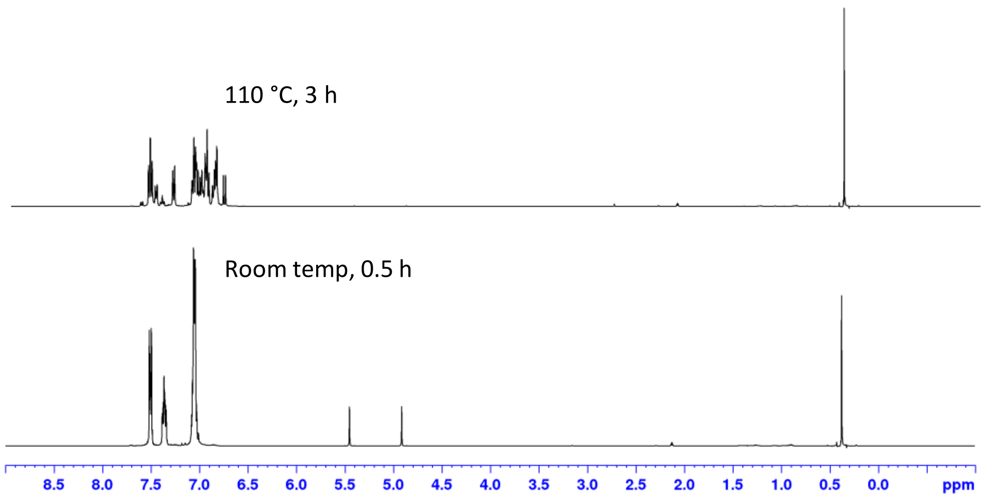


**Figure S50:** $^{1}H$ NMR spectrum for the hydrophosphination of diphenylacetylene catalysed by **3** (10 mol%), in $d_8$-toluene

## 1-phenyl-1-propyne catalysed by **1**

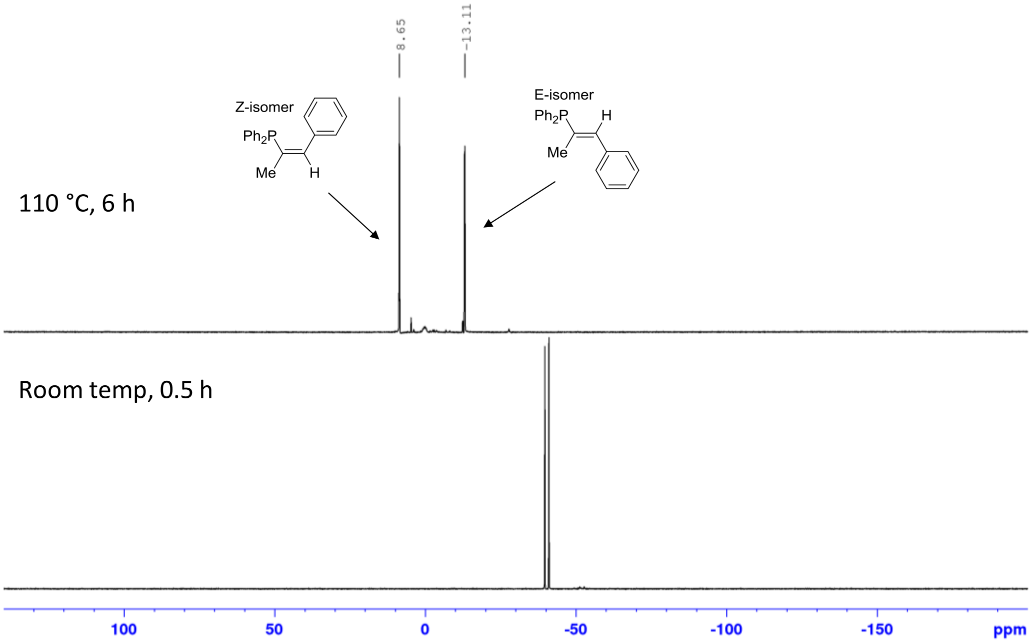


**Figure S51:** $^{31}P$ NMR spectrum for the hydrophosphination of 1-phenyl-1-propyne catalysed by **1** (5 mol% of dimer), in $d_8$-toluene

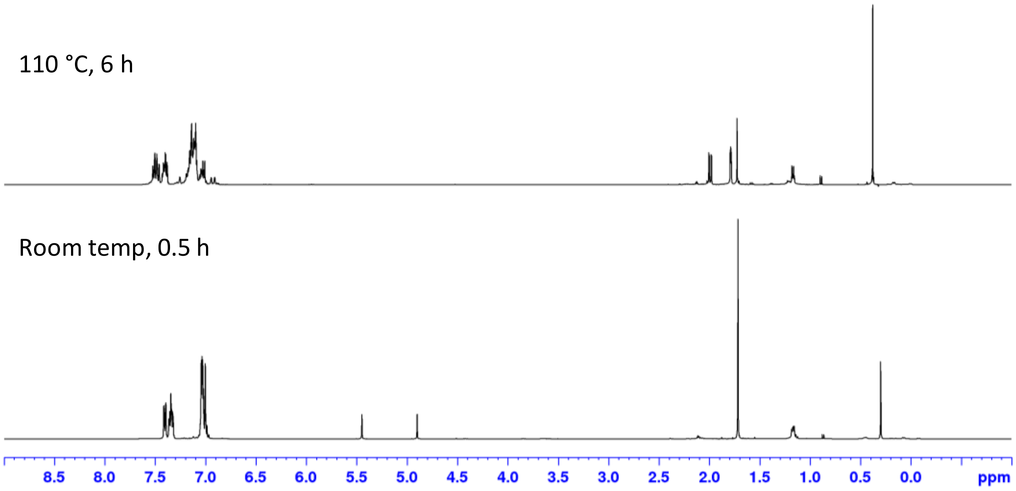


**Figure S52:** $^{1}H$ NMR spectrum for the hydrophosphination of 1-phenyl-1-propyne catalysed by **1** (5 mol% of dimer), in $d_8$-toluene

## 1-phenyl-1-propyne catalysed by **2**

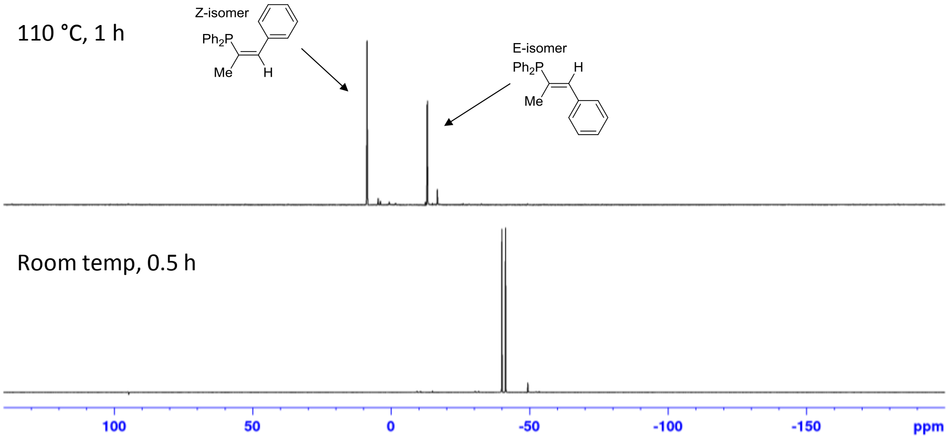


**Figure S53:** $^{31}P$ NMR spectrum for the hydrophosphination of 1-phenyl-1-propyne catalysed by **2** (10 mol%), in $d_8$-toluene

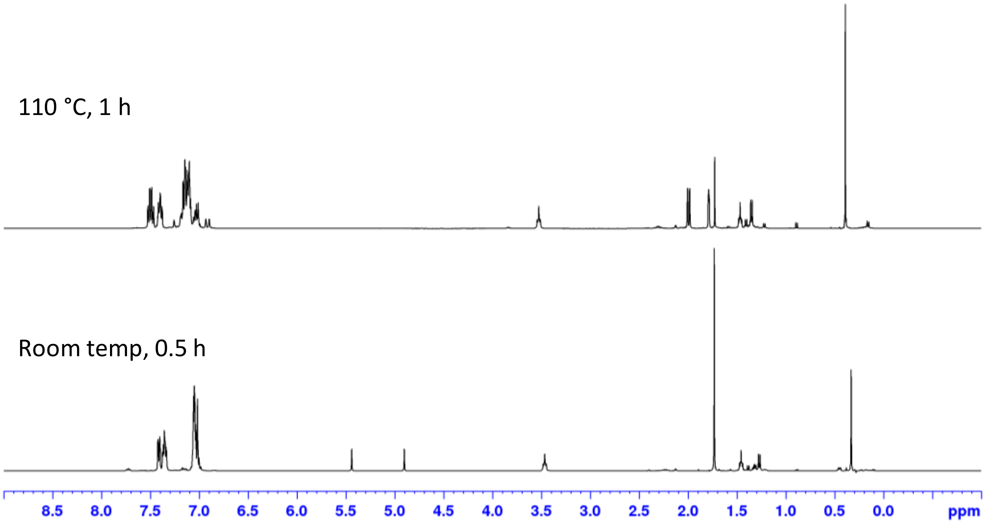


**Figure S54:** $^{1}$H NMR spectrum for the hydrophosphination of 1-phenyl-1-propyne catalysed by **2** (10 mol%), in $d_8$-toluene

The (1-methyl-2-phenylethenyl)diphenylphosphine product was isolated by column chromatography (silica gel 1:19 EtOAc:hexane). Isolated yield 0.119 g; 0.39 mmol; 79 %.

$^{1}$H NMR (600 MHz, $CDCl_3$, 300K): δ 1.69 (dd, J = 1.46 Hz, 2.82 Hz, 3H, $CH_3$ *E*-isomer); 1.90 (dd, J = 1.32 Hz, 9.01 Hz, 3H, $CH_3$ *Z*-isomer); 6.60 (d, J = 13.36 Hz, 1H C(**H**)=C *Z*-isomer); 7.10 – 7.12 (td, J = 1.87 Hz, 9.01 Hz, 2H, Ar H); 7.16 – 7.26 (m, 19H, Ar H); 7.28 – 7.31 (m, 4H, Ar H); 7.34 – 7.36 (td, J = 1.75 Hz, 7.73 Hz, 4H, Ar H) ppm. $^{1}$H COSY correlation assigns C(**H**)=C *E*-isomer resonance at 7.26 ppm.

$^{31}$P NMR (243 MHz, $CDCl_3$, 300K): δ 8.4 (s, *Z*-isomer); -13.2 (s, *E*-isomer) ppm.

$^{13}$C NMR (151 MHz, $CDCl_3$, 300K): δ 18.0 (d, J = 17.15 Hz, $CH_3$ *Z*-isomer); 24.5 (d, J = 3.71 Hz, $CH_3$ *E*-isomer); 127.1 (s, Ar CH); 127.4 (s, Ar C–H); 127.9 (s, Ar C–H); 128.3 (d, J = 14.27 Hz, Ar C–H); 128.5 (d, J = 6.39 Hz, Ar C–H); 128.6 (d, J = 6.09 Hz, Ar C–H); 128.9 (s, Ar C–H); 129.1 (s, Ar C–H); 129.5 (d, J = 7.30 Hz, Ar C–H); 133.3 (d, J = 18.86 Hz, Ar C–H); 133.9 (d, J = 18.86 Hz, Ar C–H); 136.1 (d, J = 14.20 Hz, quat. C); 136.3 (d, J = 12.07 Hz; quat. C); 137.0 (d, J = 12.36 Hz, quat. C); 137.5 (d, J = 6.54 Hz, quat. C); 137.8 (d, J = 12.18 Hz, quat. C); 139.1 (d, J = 28.39 Hz, **C**(H)=C *Z*-isomer); 143.4 (d, J = 29.07 Hz, **C**(H)=C *E*-isomer) ppm.

m/z (GCMS EI): 301.1 $[M - H]^+$

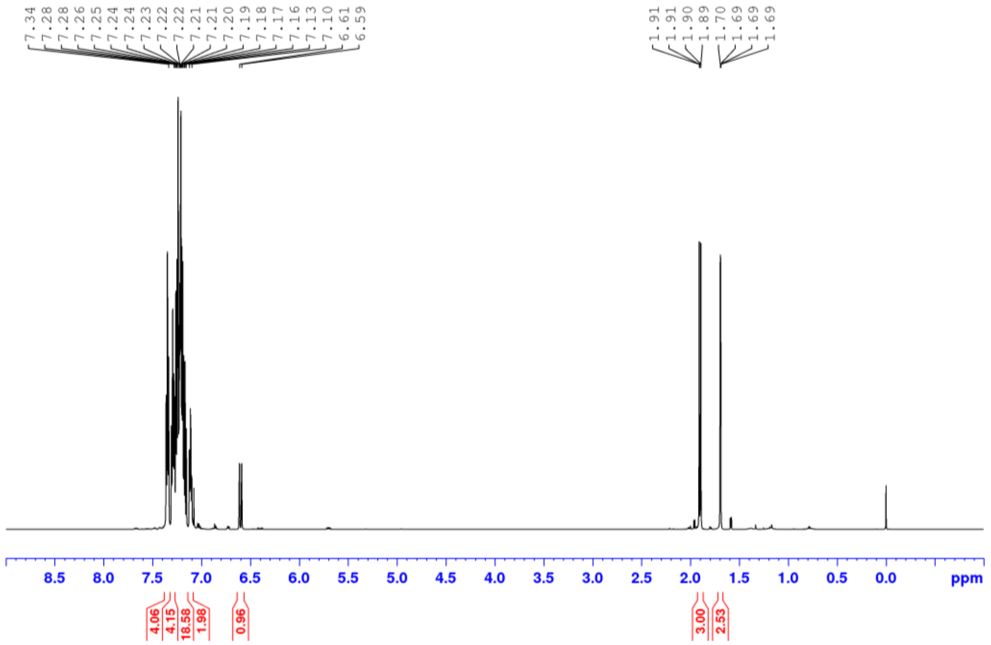


**Figure S55:** $^{1}H$ NMR spectrum for isolated $Me(PPh_2)C{=}C(H)Ph$, in $CDCl_3$

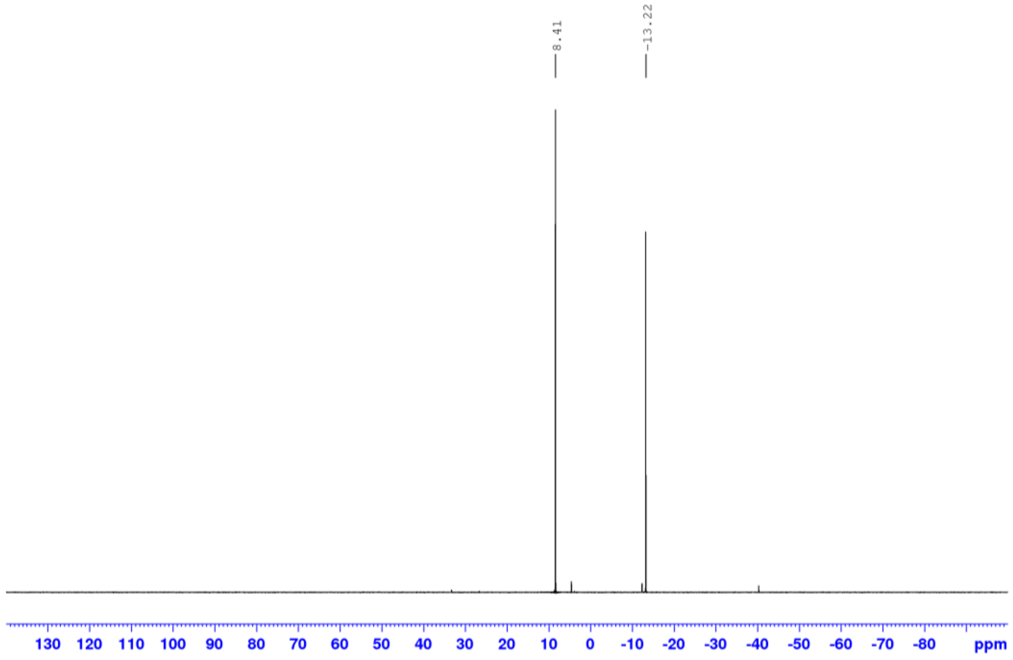


**Figure S56:** $^{31}P$ NMR spectrum for isolated $Me(PPh_2)C{=}C(H)Ph$, in $CDCl_3$

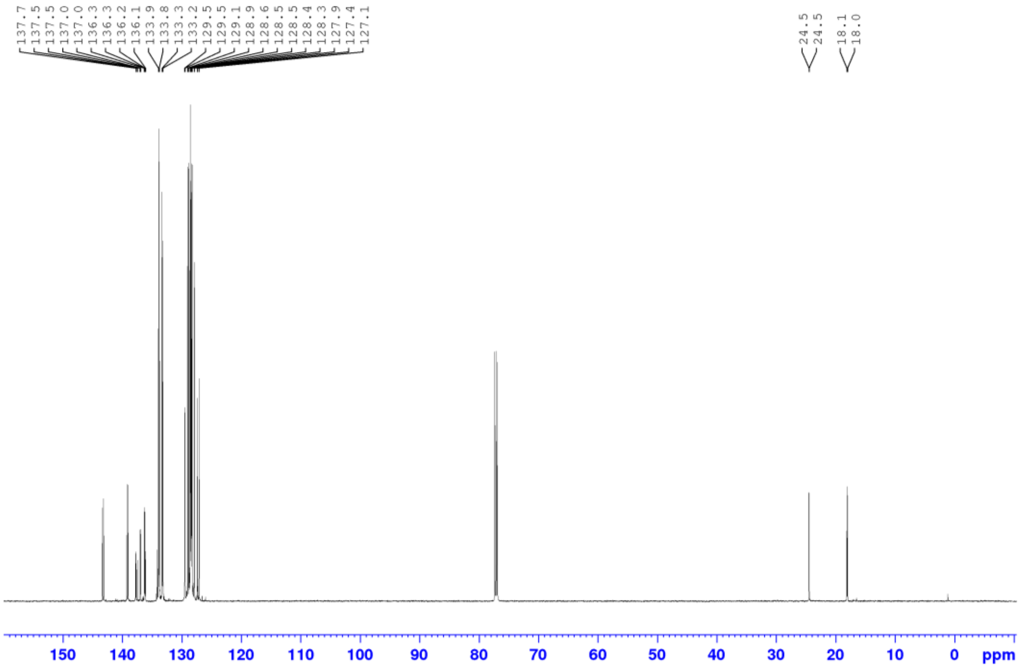


**Figure S57:** $^{13}$C NMR spectrum for isolated $Me(PPh_2)C{=}C(H)Ph$, in $CDCl_3$

## 1-phenyl-1-propyne catalysed by **3**

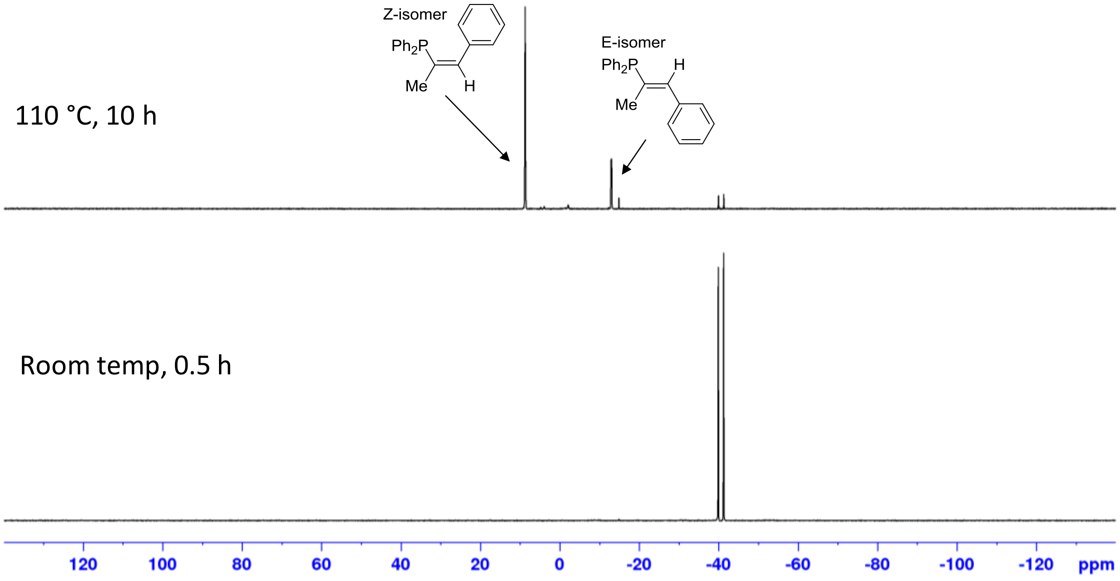


**Figure S58:** $^{31}$P NMR spectrum for the hydrophosphination of 1-phenyl-1-propyne catalysed by **3** (10 mol%), in $d_8$-toluene

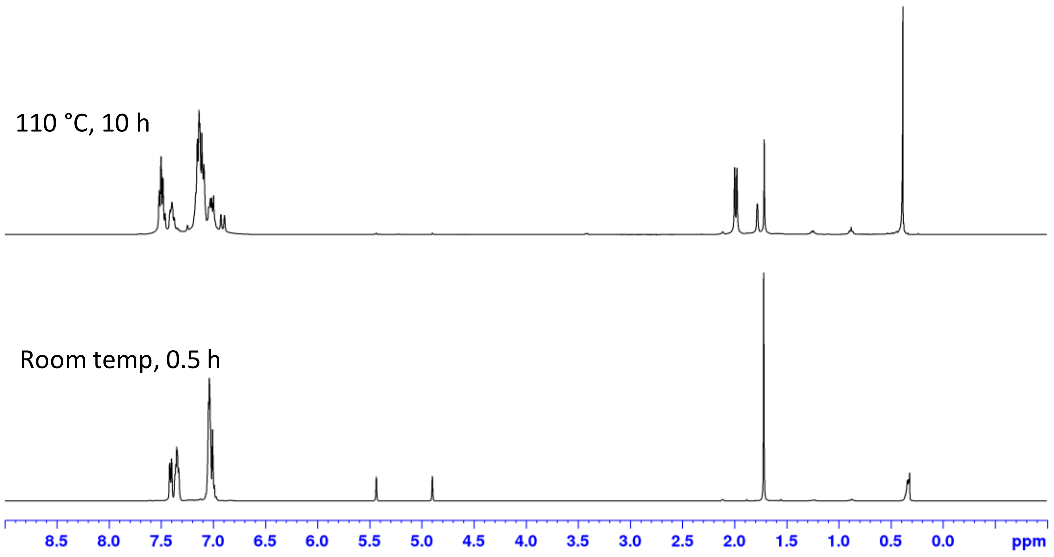


**Figure S59:** $^{1}H$ NMR spectrum for the hydrophosphination of 1-phenyl-1-propyne catalysed by **3** (10 mol%), in $d_8$-toluene

## Hydrophosphination of alkenes

### Styrene catalysed by **1**

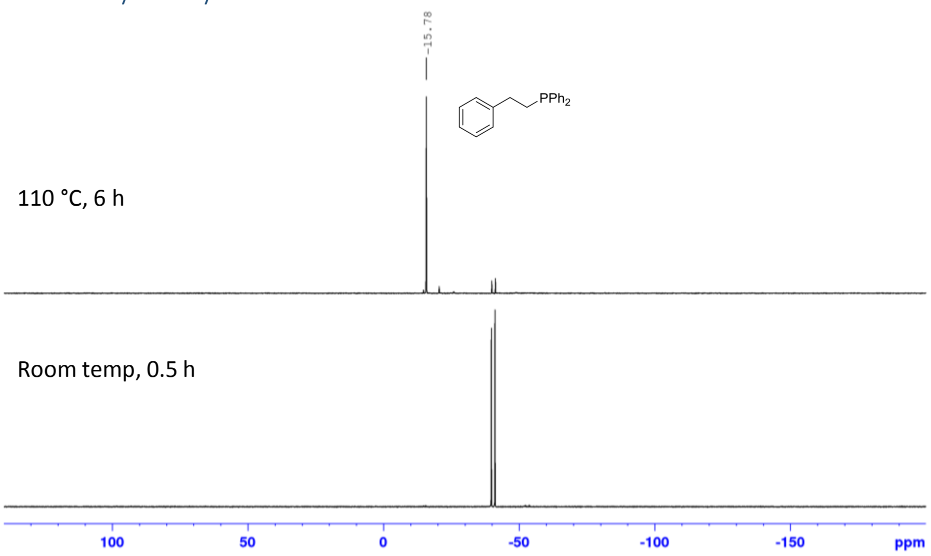


**Figure S60:** $^{31}P$ NMR spectrum for the hydrophosphination of styrene catalysed by **1** (5 mol% of dimer), in $d_8$-toluene

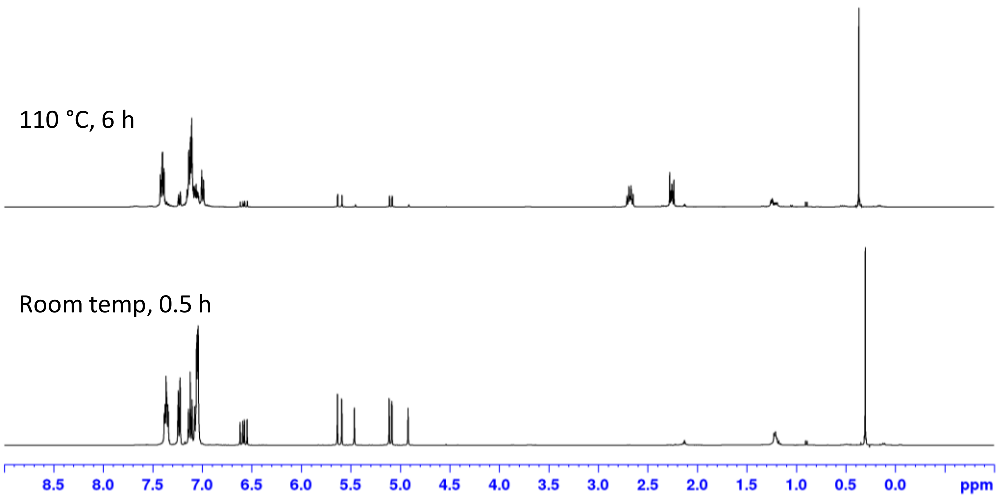


**Figure S61:** $^{1}H$ NMR spectrum for the hydrophosphination of styrene catalysed by **1** (5 mol% of dimer), in $d_8$-toluene

## Styrene catalysed by **2**

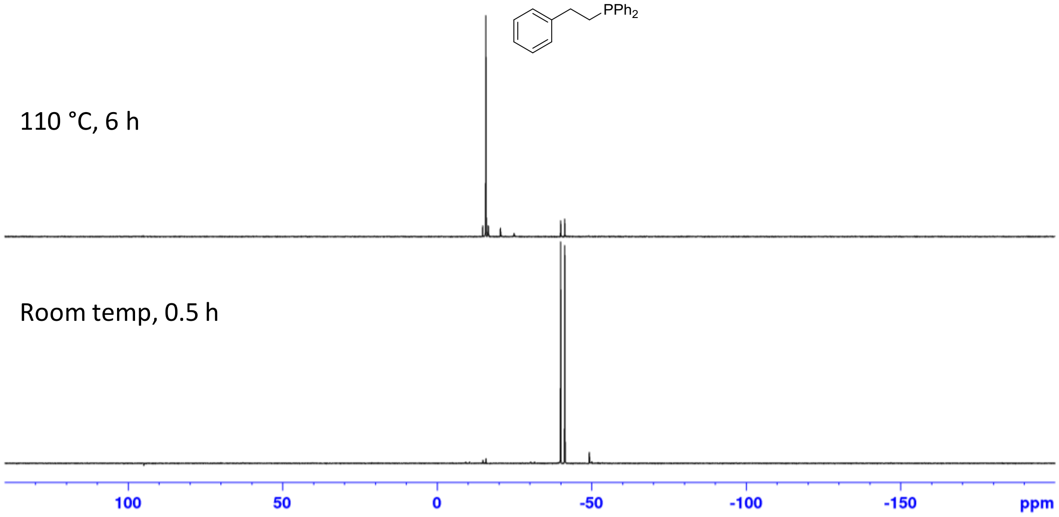


**Figure S62:** $^{31}P$ NMR spectrum for the hydrophosphination of styrene catalysed by **2** (10 mol%), in $d_8$-toluene

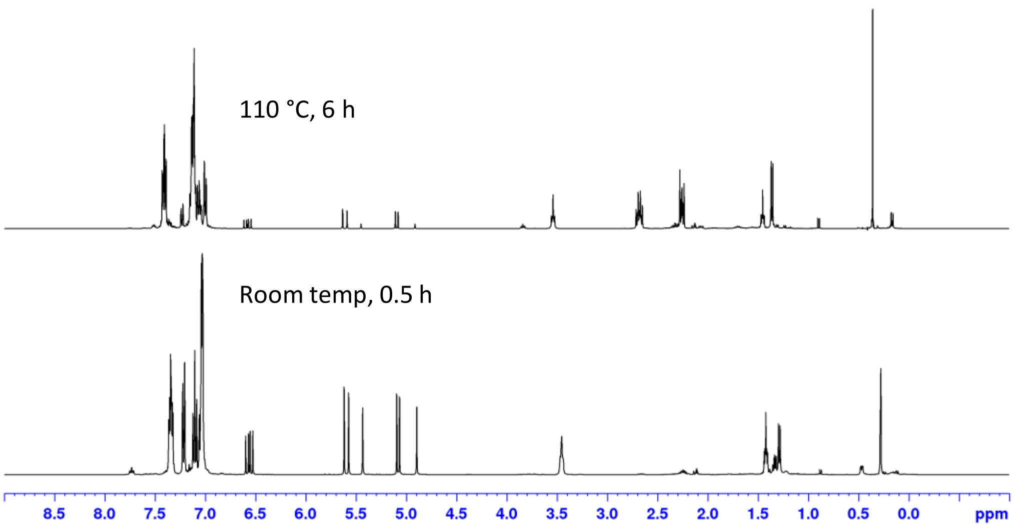


**Figure S63:** $^{1}$H NMR spectrum for the hydrophosphination of styrene catalysed by **2** (10 mol%), in $d_8$-toluene

The diphenyl(2-phenylethyl)phosphine product was isolated column chromatography (silica gel 1:19 EtOA:hexane). Isolated yield 0.082 g; 0.3 mmol; 60 %

$^{1}$H NMR (600 MHz, $CDCl_3$, 300K): δ 2.32 – 2.35 (m, 2H, C**H**$_2$–PPh$_2$); 2.68 – 2.72 (m, 2H, Ph–C**H**$_2$); 7.12 – 7.15 (m, 3H, Ar C–H); 7.21 – 7.24 (m, 2H, Ar C–H); 7.27 – 7.30 (m, 6H, Ar C–H); 7.41 – 7.44 (m, 4H Ar C–H) ppm.

$^{31}$P NMR (243 MHz, $CDCl_3$, 300K): δ -15.7 ppm.

$^{13}$C NMR (151 MHz, $CDCl_3$, 300K): δ 30.3 (d; J = 13.08 Hz, **C**H$_2$–PPh$_2$); 32.2 (d; J = 17.31 Hz, Ph–**C**H$_2$); 126.1 (s, Ar C–H); 128.2 (s, Ar C–H); 128.5 (s, Ar C–H); 128.6 (s, Ar C–H); 128.7 (s, Ar C–H); 132.8 (d, J = 17.51 Hz, Ar C–H); 138.6 (d, J = 13.37 Hz, ipso P–C(Ar)); 142.6 (d, J = 13.54 Hz, ipso Ph) ppm.

m/z (GCMS EI): 289.1 $[M - H]^+$

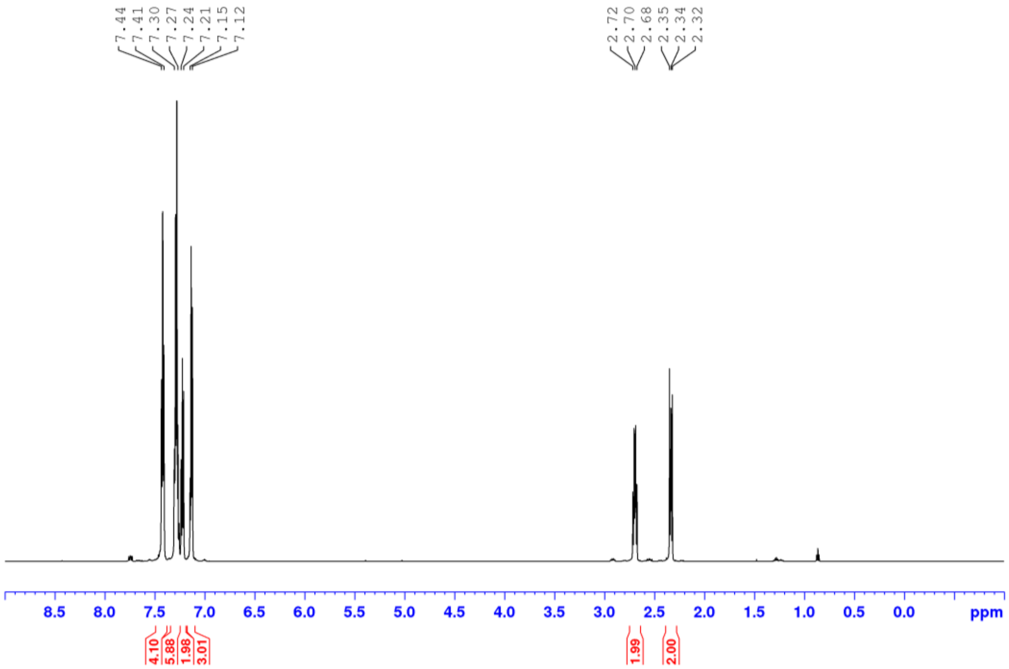


**Figure S64:** $^{1}H$ NMR spectrum for isolated $PhCH_2CH_2PPh_2$, in $CDCl_3$

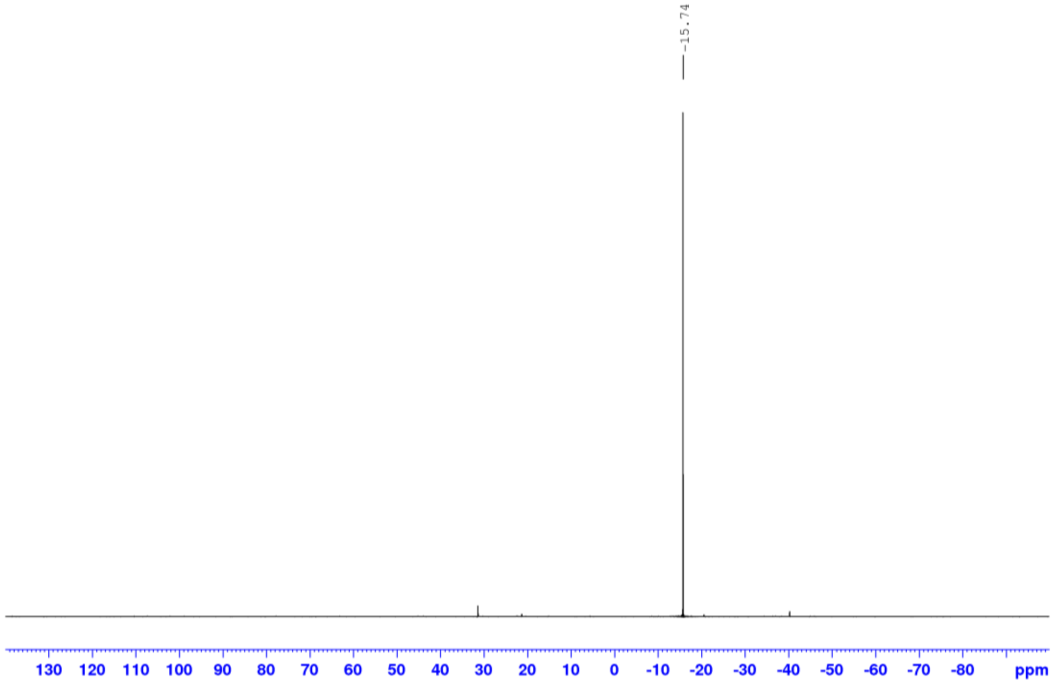


**Figure S65:** $^{31}P$ NMR spectrum for isolated $PhCH_2CH_2PPh_2$, in $CDCl_3$

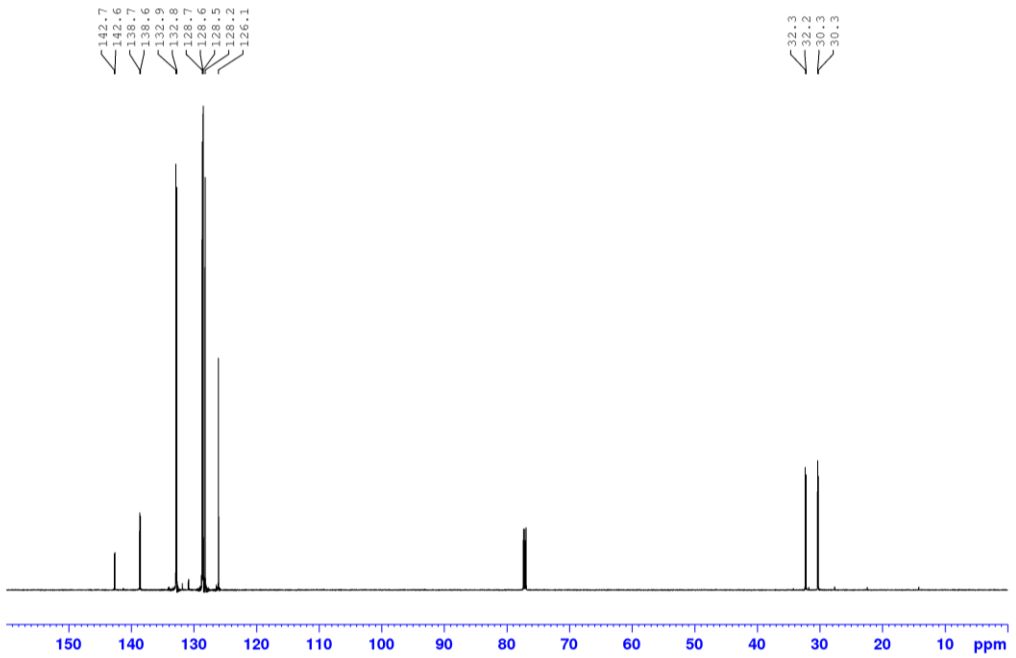


**Figure S66:** $^{13}C$ NMR spectrum for isolated $PhCH_2CH_2PPh_2$, in $CDCl_3$

## Styrene catalysed by **3**

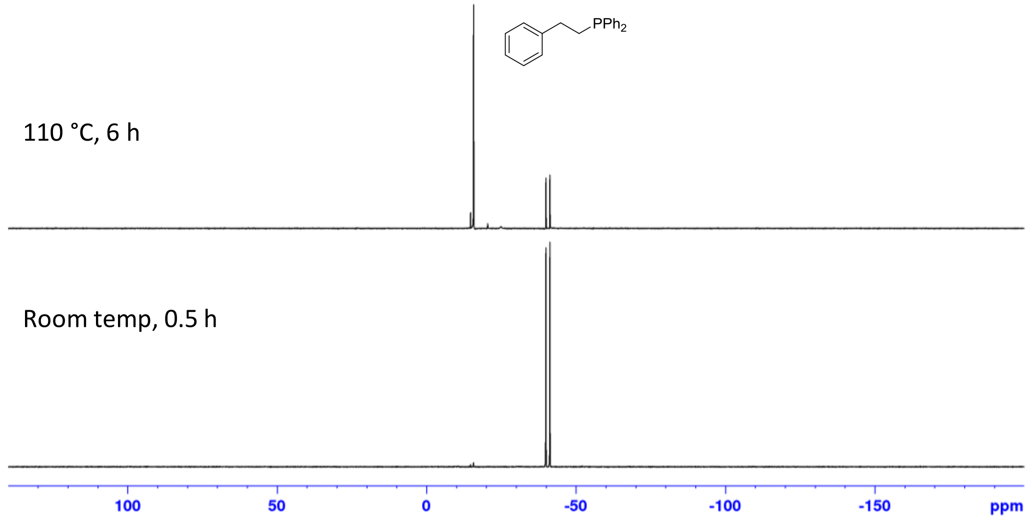


**Figure S67:** $^{31}P$ NMR spectrum for the hydrophosphination of styrene catalysed by **3** (10 mol%), in $d_8$-toluene

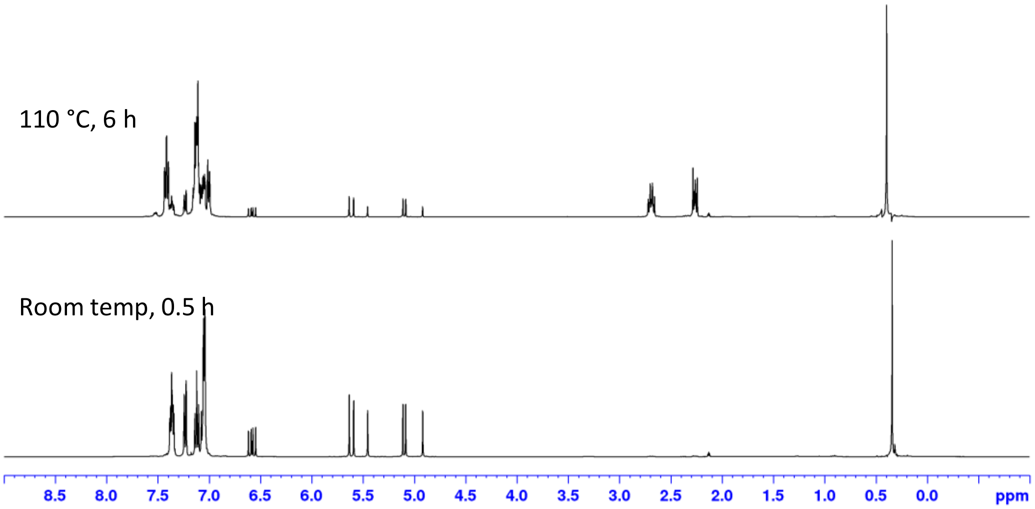


**Figure S68:** $^{1}H$ NMR spectrum for the hydrophosphination of styrene catalysed by **3** (10 mol%), in $d_8$-toluene

## 4-fluoro styrene catalysed by 2

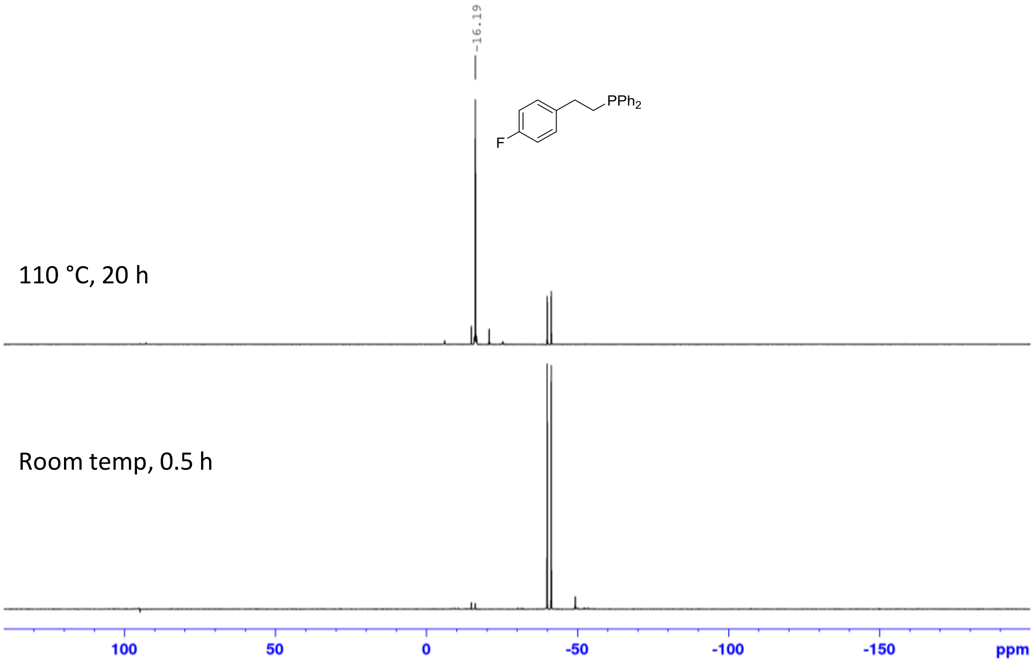


**Figure S69:** $^{31}P$ NMR spectrum for the hydrophosphination of 4-fluoro styrene catalysed by **2** (10 mol%), in $d_8$-toluene

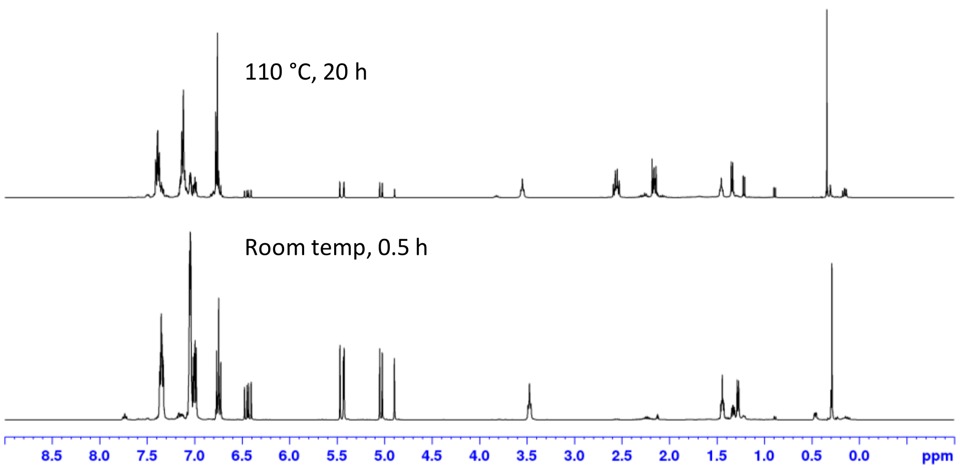


**Figure S70:** $^{1}H$ NMR spectrum for the hydrophosphination of 4-fluoro styrene catalysed by **2** (10 mol%), in $d_8$-toluene

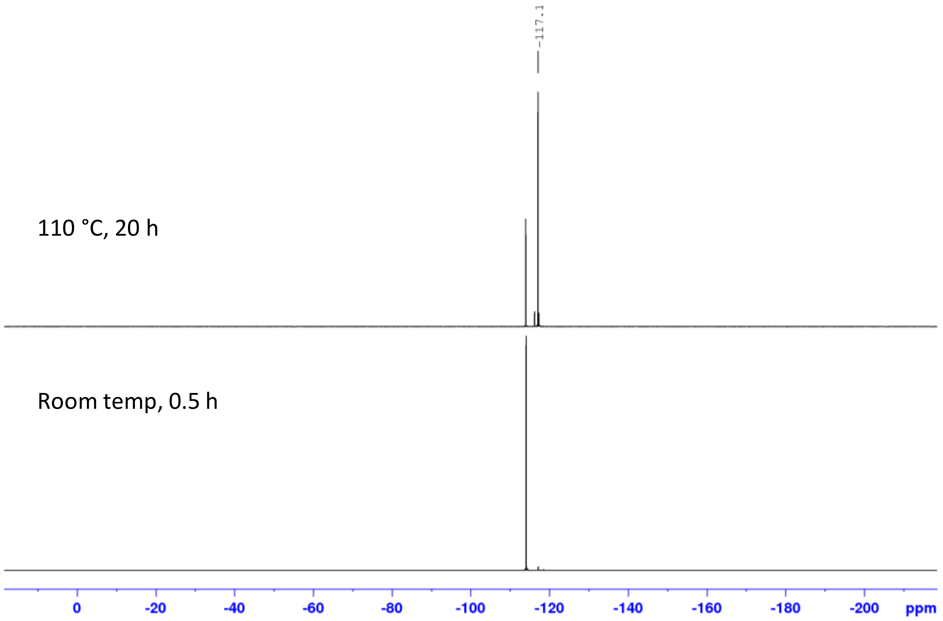


**Figure S71:** $^{19}F$ NMR spectrum for the hydrophosphination of 4-fluoro styrene catalysed by **2** (10 mol%), in $d_8$-toluene

## 4-chloro styrene catalysed by **2**

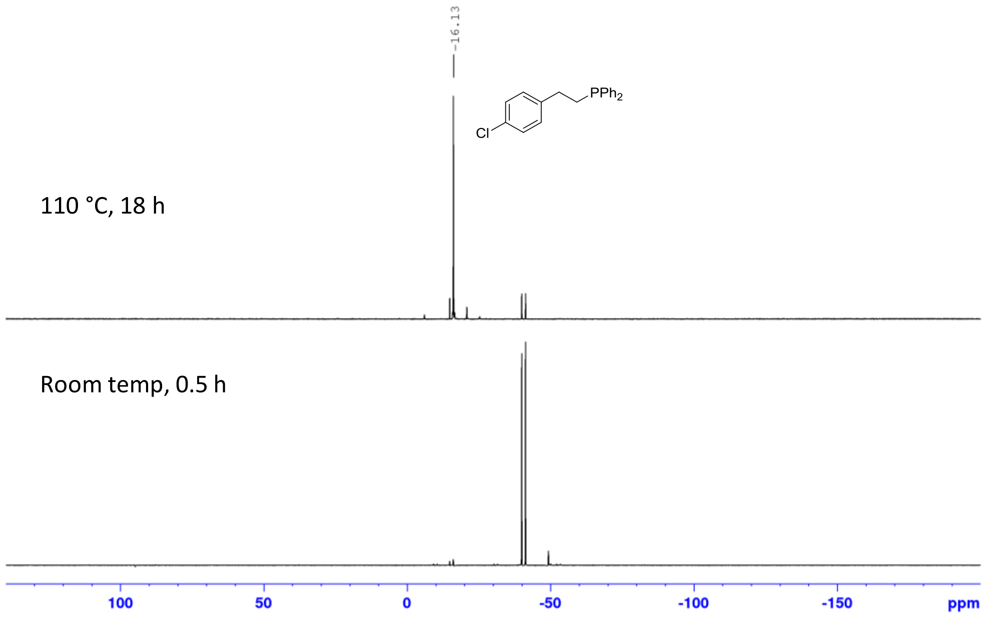


**Figure S72:** $^{31}P$ NMR spectrum for the hydrophosphination of 4-chloro styrene catalysed by **2** (10 mol%), in $d_8$-toluene

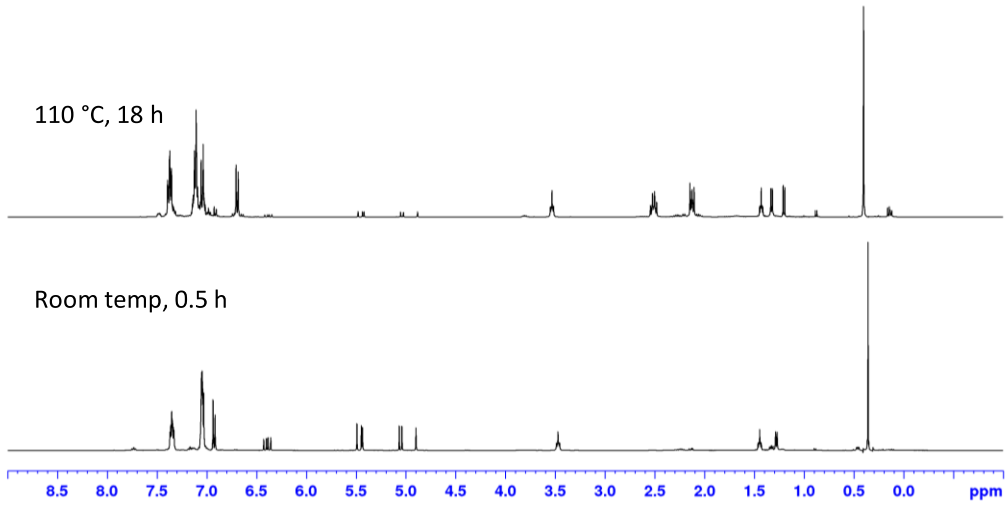


**Figure S73:** $^{1}H$ NMR spectrum for the hydrophosphination of 4-chloro styrene catalysed by **2** (10 mol%), in $d_8$-toluene

## 4-vinyl anisole catalysed by **2**

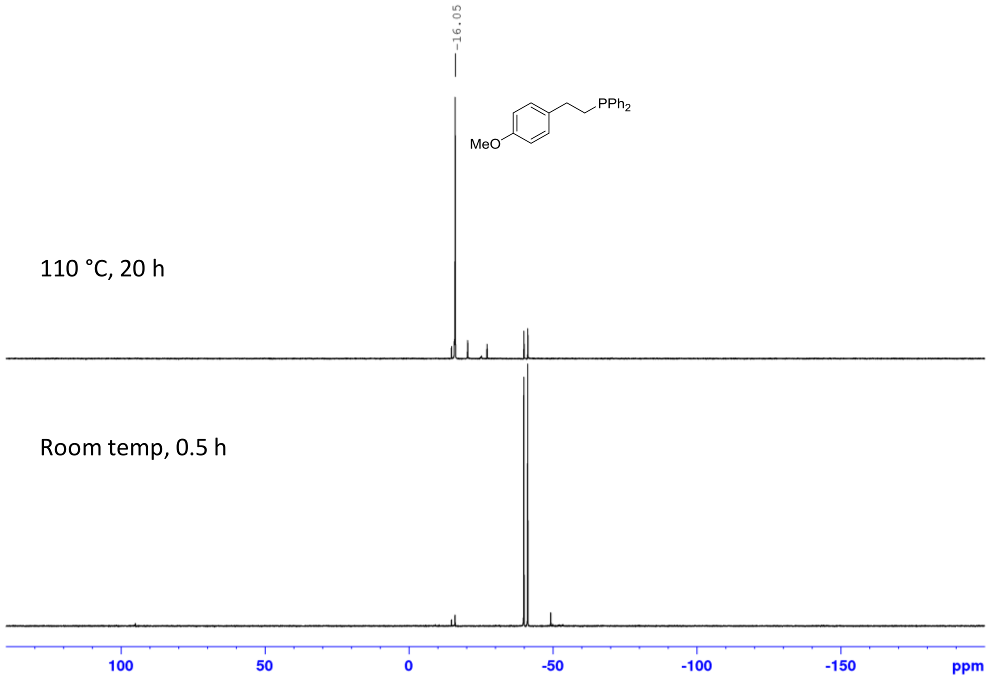


**Figure S74:** $^{31}P$ NMR spectrum for the hydrophosphination of 4-vinyl anisole catalysed by **2** (10 mol%), in $d_8$-toluene

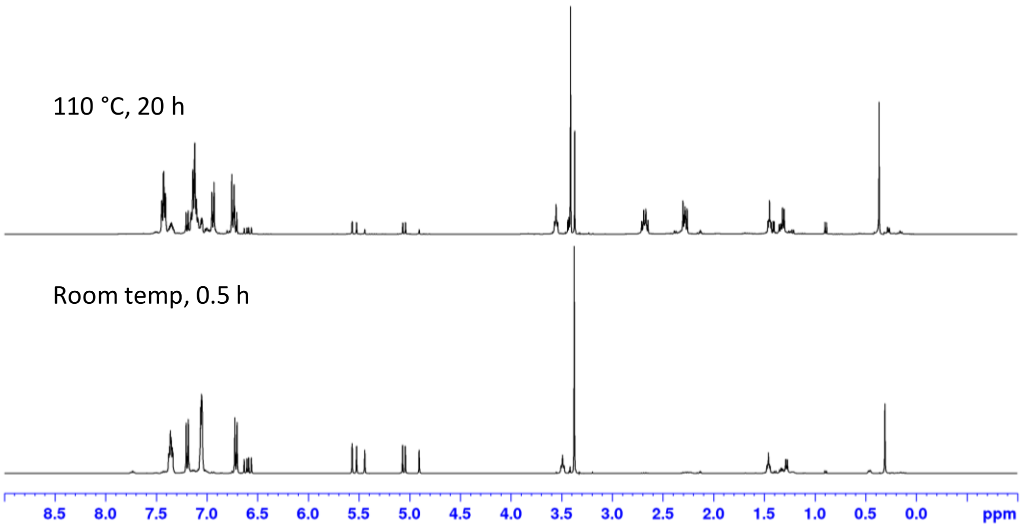


**Figure S75:** $^{1}H$ NMR spectrum for the hydrophosphination of 4-vinyl anisole catalysed by **2** (10 mol%), in $d_8$-toluene

## Vinyl boronic acid pinacol ester catalysed by **2**

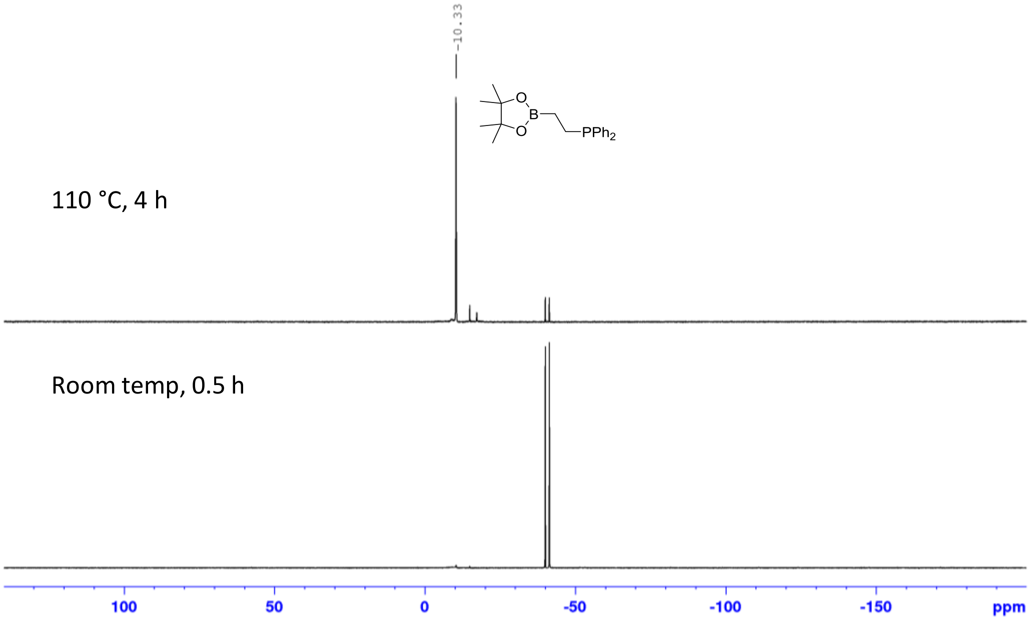


**Figure S76:** $^{31}$P NMR spectrum for the hydrophosphination of vinyl boronic acid pinacol ester catalysed by **2** (10 mol%), in $d_8$-toluene

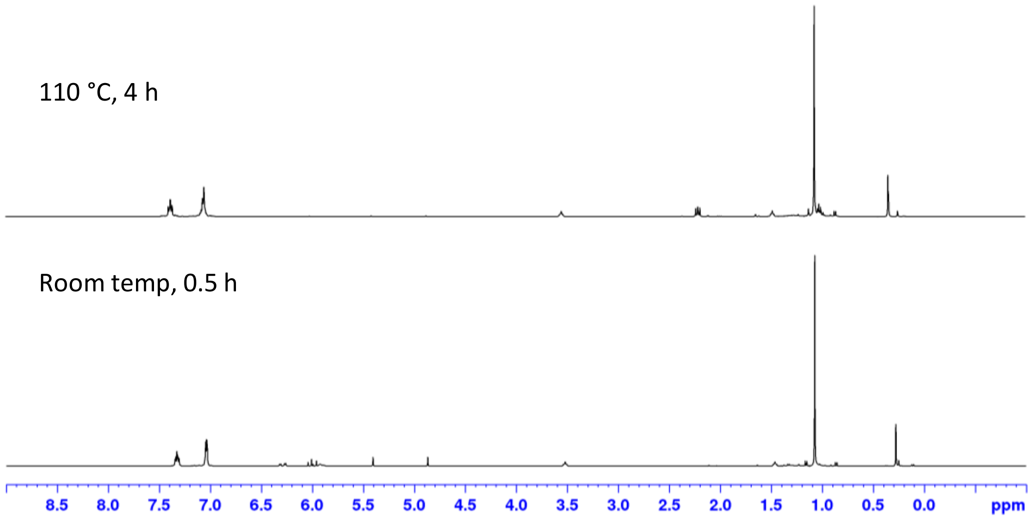


**Figure S77:** $^{1}$H NMR spectrum for the hydrophosphination of vinyl boronic acid pinacol ester catalysed by **2** (10 mol%), in $d_8$-toluene

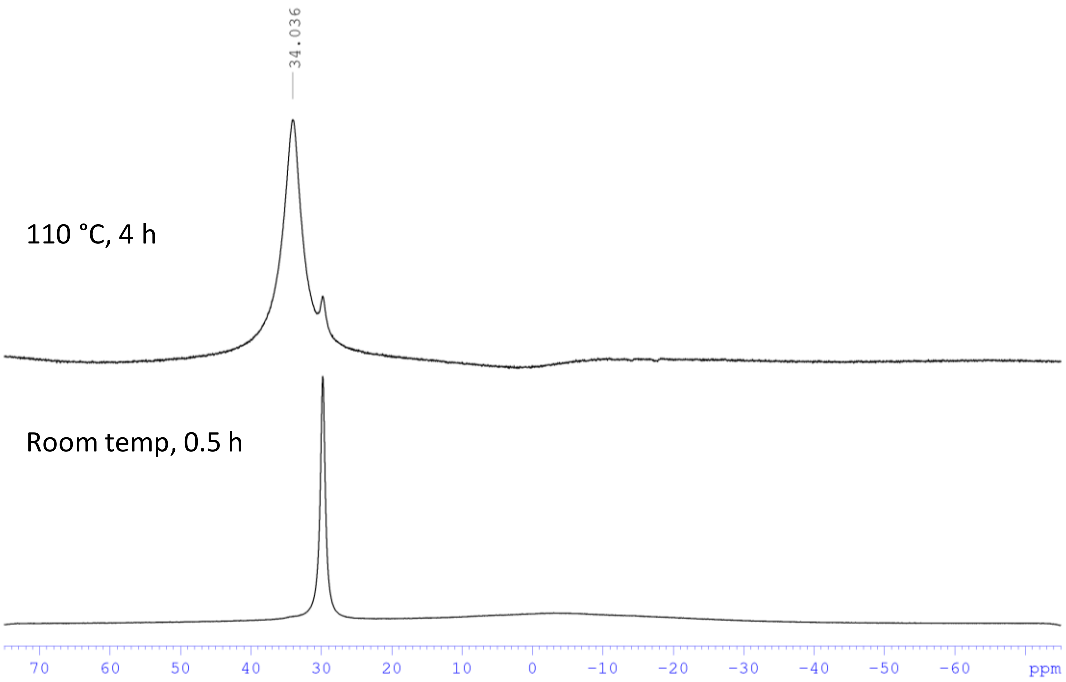


**Figure S78:** $^{11}B$ NMR spectrum for the hydrophosphination of vinyl boronic acid pinacol ester catalysed by **2** (10 mol%), in $d_8$-toluene

## Hydrophosphination of carbodiimides

### N,N-diisopropyl carbodiimide catalysed by 2

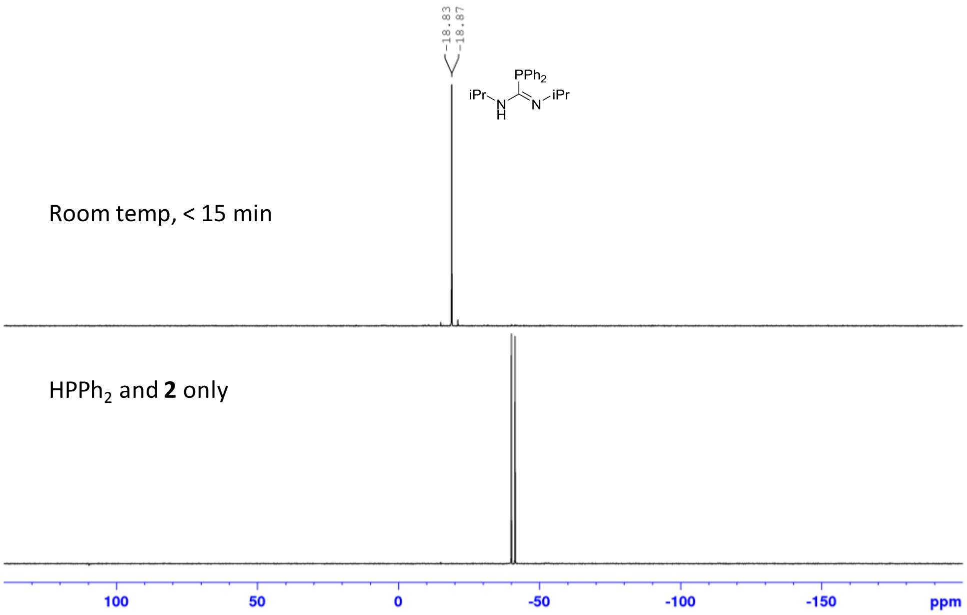


**Figure S79:** $^{31}P$ NMR spectrum for the hydrophosphination of diisopropyl carbodiimide catalysed by **2** (5 mol%), in $d_8$-toluene

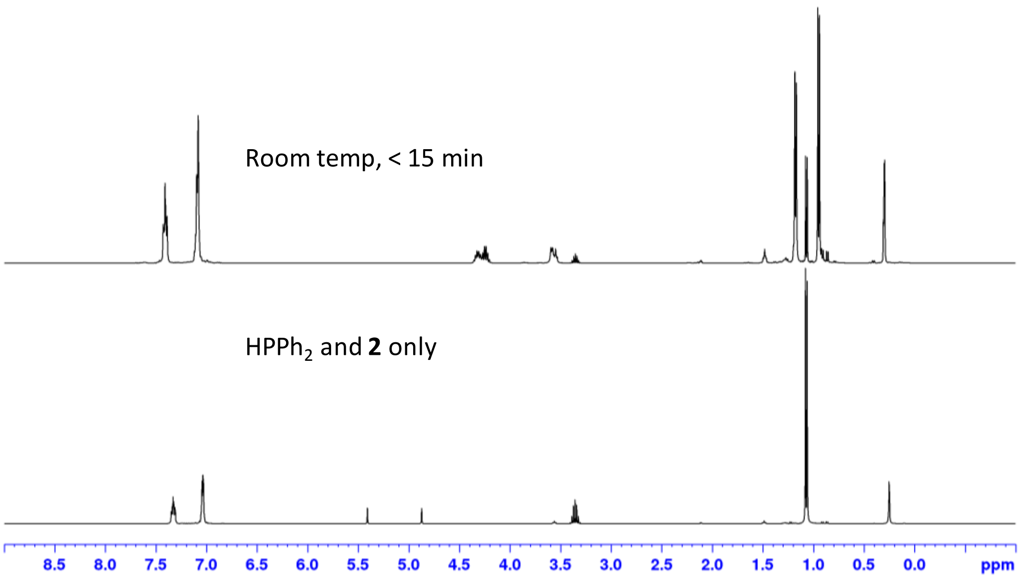


**Figure S80:** $^{1}$H NMR spectrum for the hydrophosphination of diisopropyl carbodiimide catalysed by **2** (5 mol%), in $d_8$-toluene

The *N,N*-diisopropyl-1,1-diphenylphopshinecarboximidamide product was isolated via recrystalising from hexane solution. Isolated crystalline yield 0.123 g; 0.4 mmol; 80 %

$^{1}$H NMR (400 MHz, $C_6D_6$, 300K): δ 0.96 (d, J = 6.51 Hz, 6H, CH(C**H₃**)₂); 1.27 (d, J = 6.10 Hz, 6H, CH(C**H₃**)₂); 3.66 (br d, J = 6.75 Hz, 1H, N–H); 4.32 (hept., J = 6.44, 1H, C**H**(CH₃)₂); 4.42 (hept., J = 6.17 Hz, C**H**(CH₃)₂); 7.00 – 7.07 (m, 6H, Ar C–H); 7.47 (td, J = 1.65 Hz, 7.67 Hz, 4H, Ar C–H) ppm.

$^{31}$P NMR (162.0 MHz, $C_6D_6$, 300K): δ – 18.7 ppm.

$^{13}$C NMR (100 MHz, $C_6D_6$, 300K): δ 22.5 (s, CH(**C**H₃)₂); 25.4 (s, CH(**C**H₃)₂); 43.0 (s, **C**H(CH₃)₂); 52.2 (d, J = 35.23 Hz, **C**H(CH₃)₂); 129.1 (d, J = 7.40 Hz, Ar C–H); 129.4 (s, Ar C–H); 134.4 (d, J = 19.52 Hz, Ar C–H); 135.6 (d, J = 13.94 Hz, ipso C); 152.4 (d, J = 32.20 Hz, N=**C**(PPh₂)–N) ppm.

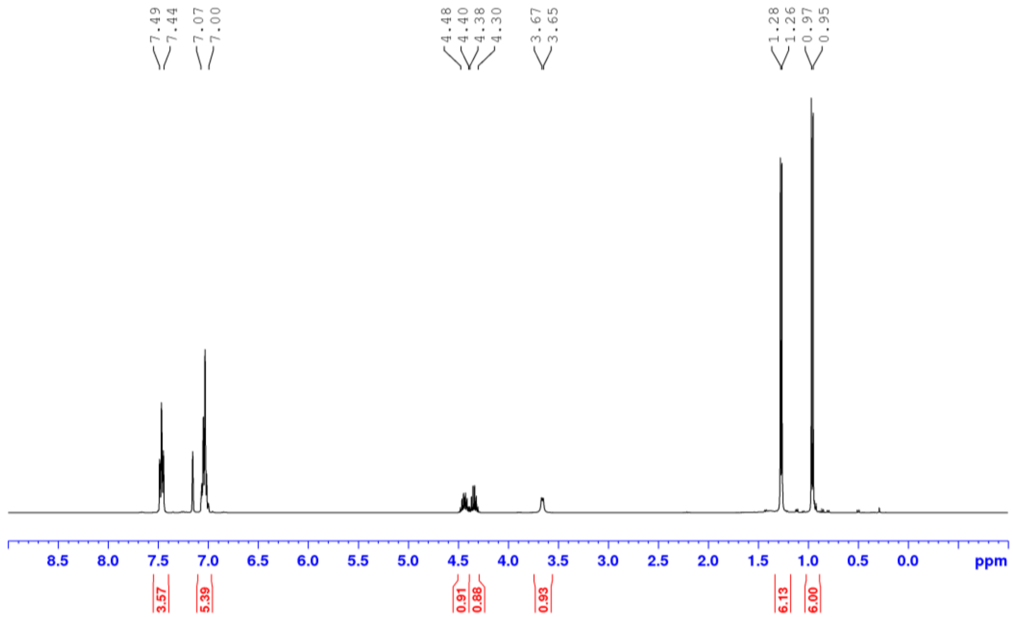


**Figure S81:** $^{1}H$ NMR spectrum for isolated iPr(H)NC(PPh$_2$)NiPr, in $C_6D_6$

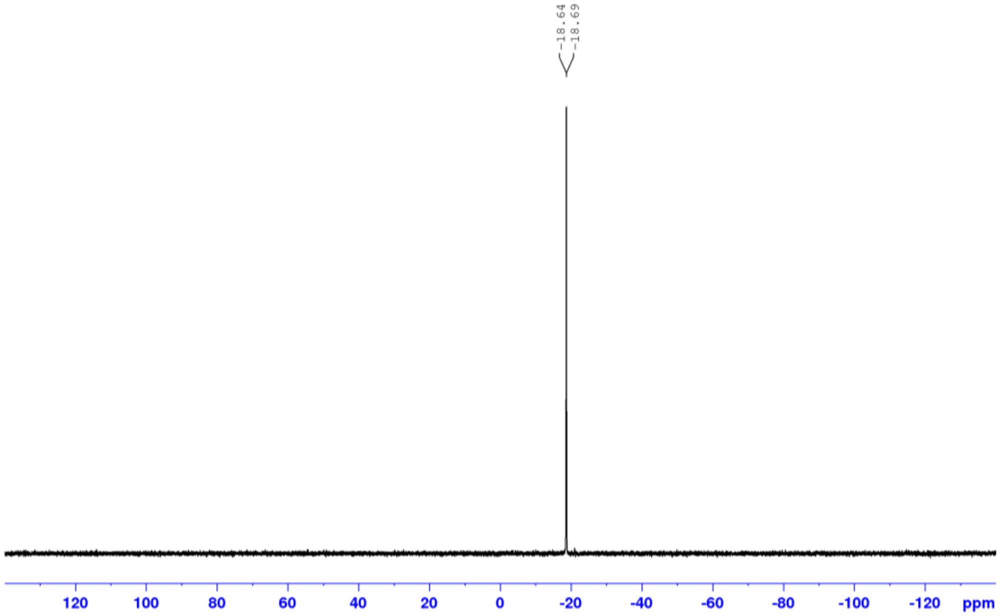


**Figure S82:** $^{31}P$ NMR spectrum for isolated iPr(H)NC(PPh$_2$)NiPr, in $C_6D_6$

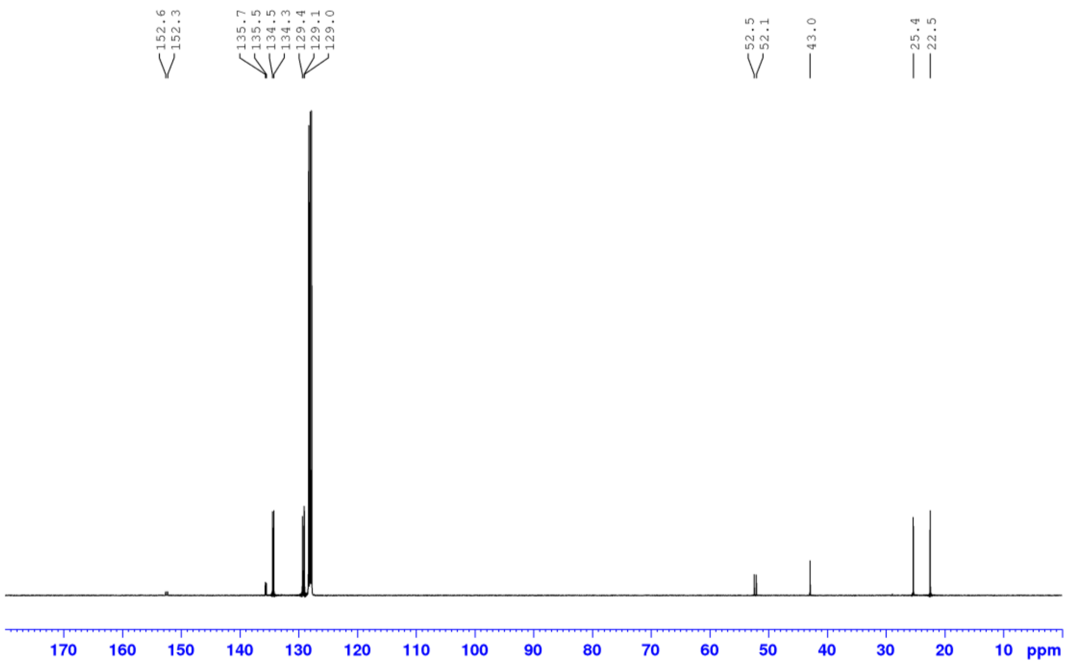


**Figure S83:** $^{13}C$ NMR spectrum for isolated iPr(H)NC($PPh_2$)NiPr, in $C_6D_6$

## N,N-dicyclohexyl carbodiimide catalysed by **2**

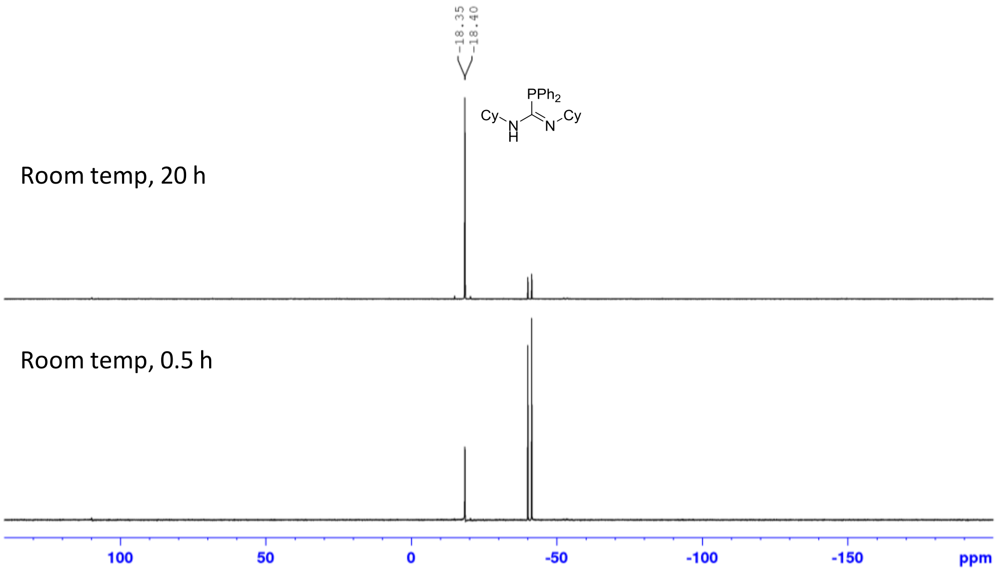


**Figure S84:** $^{31}P$ NMR spectrum for the hydrophosphination of dicyclohexyl carbodiimide catalysed by **2** (5 mol%), in $d_8$-toluene

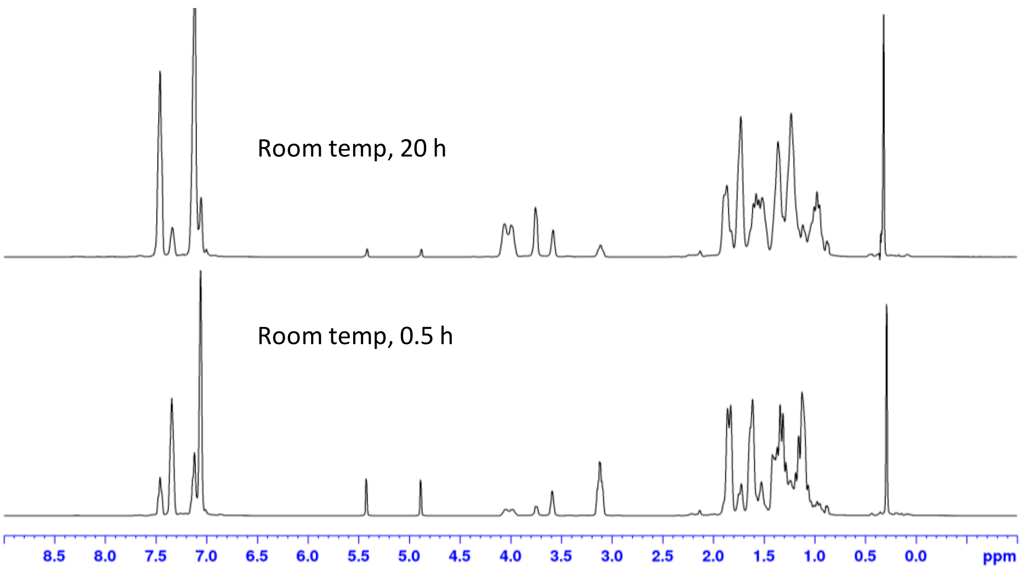


**Figure S85:** $^{1}$H NMR spectrum for the hydrophosphination of dicyclohexyl carbodiimide catalysed by **2** (5 mol%), in $d_8$-toluene

## Unreactive substrates tested

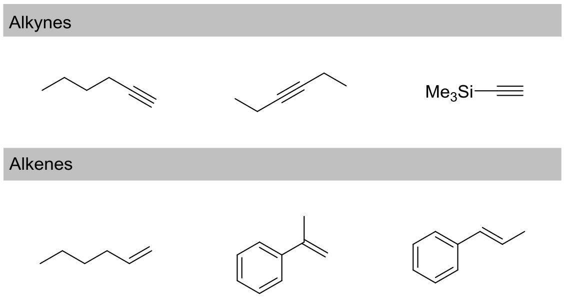


## Deuterium labelling experiment

$iBu_3AlPPh_2Li(THF)_3$ (10 mol%, 0.05 mmol, 0.0302 g) was added to 0.5 mL of $H_8$-toluene solution containing diphenyl acetylene (0.6 mmol; 0.1069 g) and **D**$PPh_2$ (0.5 mmol, 0.09 mL). This mixture was transferred to a sealed J. Young's tap NMR tube and the reaction heated at 110 °C was regularly monitored by $^{2}$H and $^{31}$P NMR spectroscopy until the complete consumption of starting materials was observed.

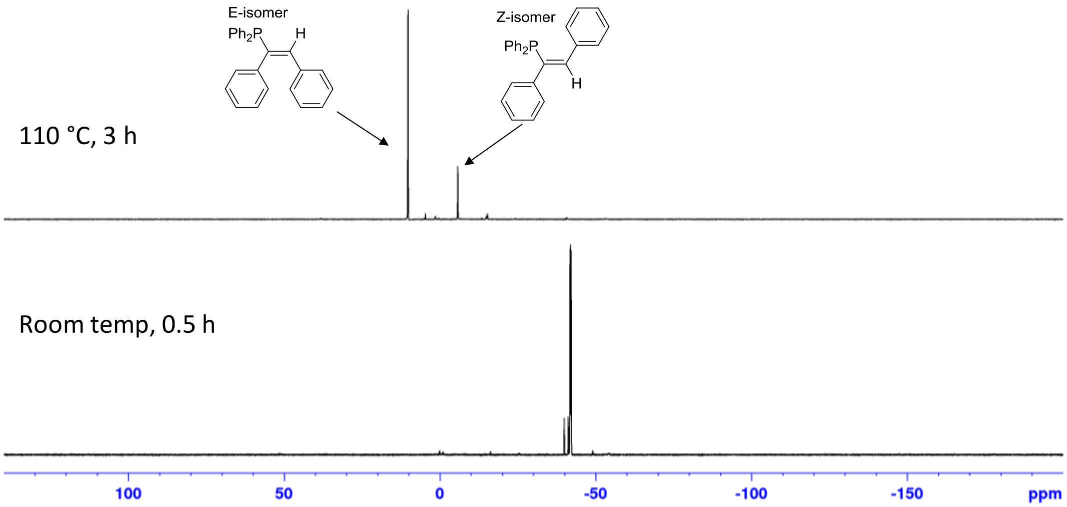


**Figure S86:** $^{31}$P NMR spectrum for the hydrophosphination of diphenyl acetylene by DPPh$_2$, catalysed by **2** (10 mol%), in H$_8$-toluene

The vinyl phosphine product was isolated by filtering over a plug of silica and recrystallizing from pentane. Isolated crystalline yield 0.1069 g; 0.3 mmol; 60 %.

$^{1}$H NMR (400 MHz, CDCl$_3$, 300 K): δ 6.90 – 6.92 (m, 2H, Ar); 7.06 – 7.10 (m, 3H, Ar); 7.15 -7.17 (m, 5H, Ar); 7.31 – 7.34 (m, 6H, Ar); 7.43 – 7.47 (m, 4H, Ar) ppm.

$^{2}$H NMR (61.4 MHz, CHCl$_3$, 300 K): δ 6.65 (s) ppm.

$^{31}$P NMR (162 MHz, CDCl$_3$, 300 K): δ 8.53 ppm.

$^{13}$C NMR (151 MHz, CDCl$_3$, 300 K): δ 127.1 (s, Ar C–H); 127.4 (s, Ar C–H); 128.0 (s, Ar C–H); 128.5 (s, Ar C–H); 128.6 (s, Ar C–H); 129.1 (s, Ar C–H); 129.2 (d, J = 6.50 Hz, Ar C–H); 129.4 (s, Ar C–H); 134.4 (d, J = 19.34 Hz, Ar C–H); 135.5 (d, J = 12.30 Hz, quat. C); 136.9 (d, J = 6.87 Hz, quat. C); 138.9 (d, J = 18.86 Hz, residual **C**(H)=C); 140.1 (d, J = 16.26 Hz, quat. C); 140.4 (d, J = 18.29 Hz, quat. C) ppm. Note **C**(D)=C could not be identified to due splitting from $^{2}$H and $^{31}$P, however weak signals in the $^{13}$C NMR spectrum in the region 137 – 138 ppm would be consistent with this signal.

m/z (GCMS EI): 364.3 [M - H]$^{+}$

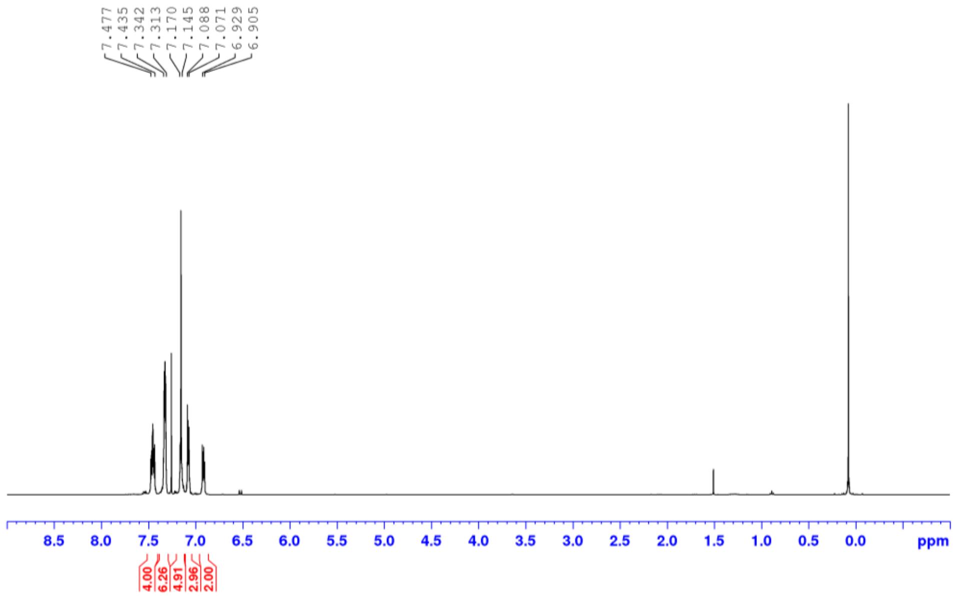


**Figure S87:** $^{1}$H NMR spectrum for isolated Ph($PPh_2$)C=C(D)Ph, in $CDCl_3$

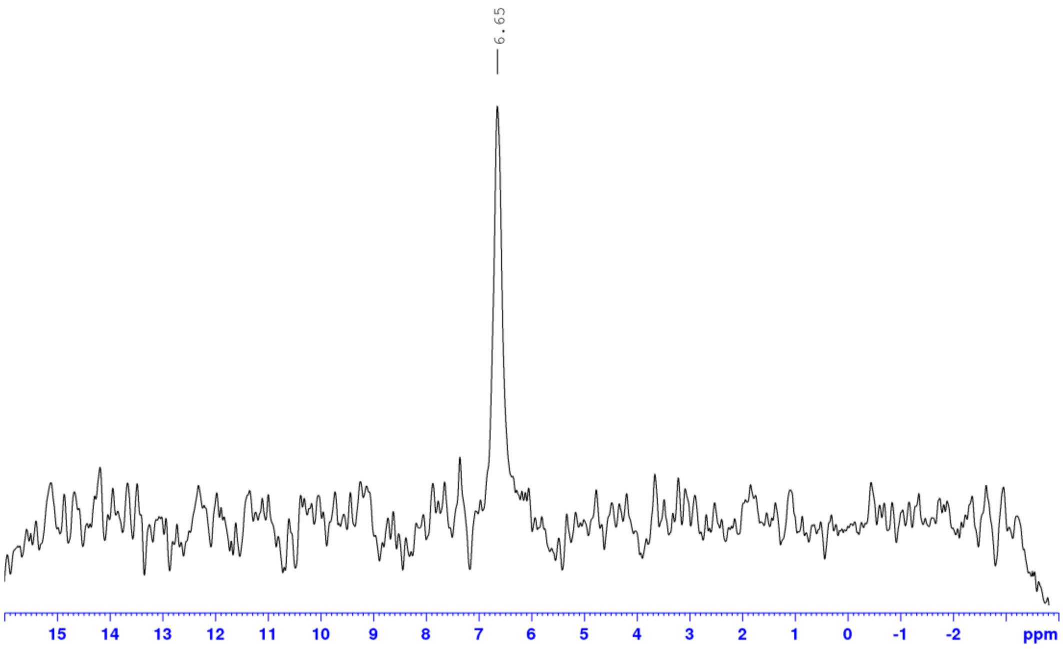


**Figure S88:** $^{2}$H NMR spectrum for isolated Ph($PPh_2$)C=C(D)Ph, in $CHCl_3$

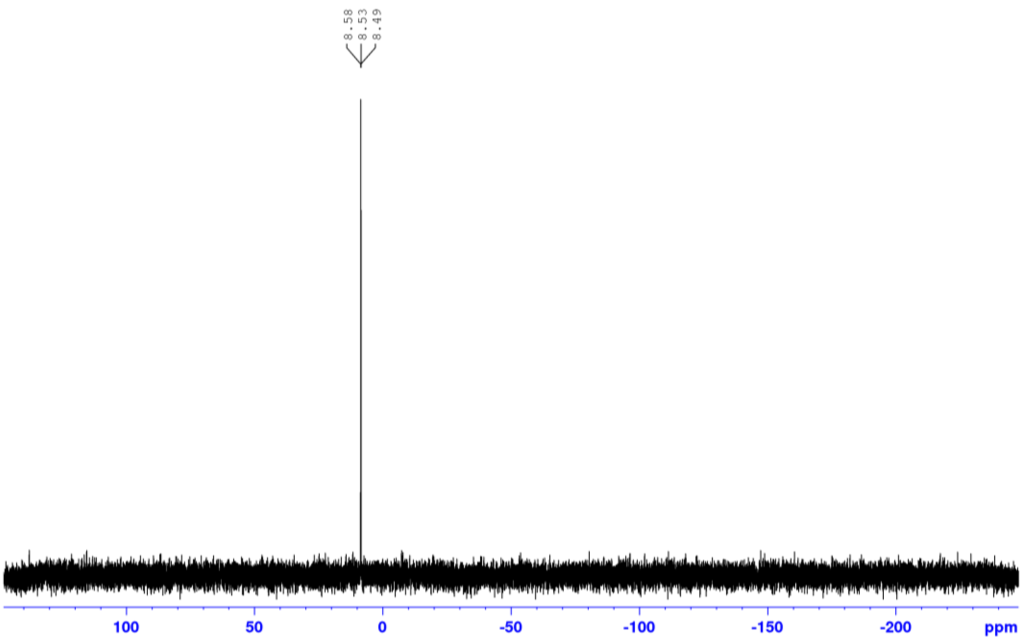


**Figure S89:** $^{31}$P NMR spectrum for isolated $Ph(PPh_2)C{=}C(D)Ph$, in $CDCl_3$

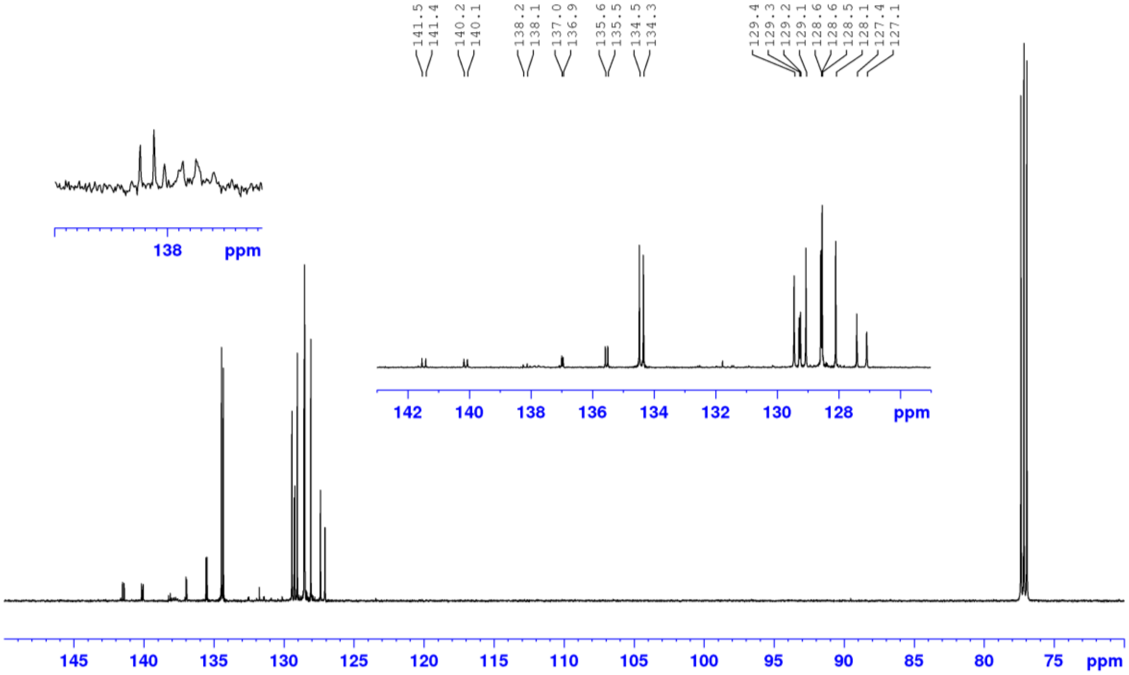


**Figure S90:** $^{13}$C NMR spectrum for isolated $Ph(PPh_2)C{=}C(D)Ph$, in $CDCl_3$

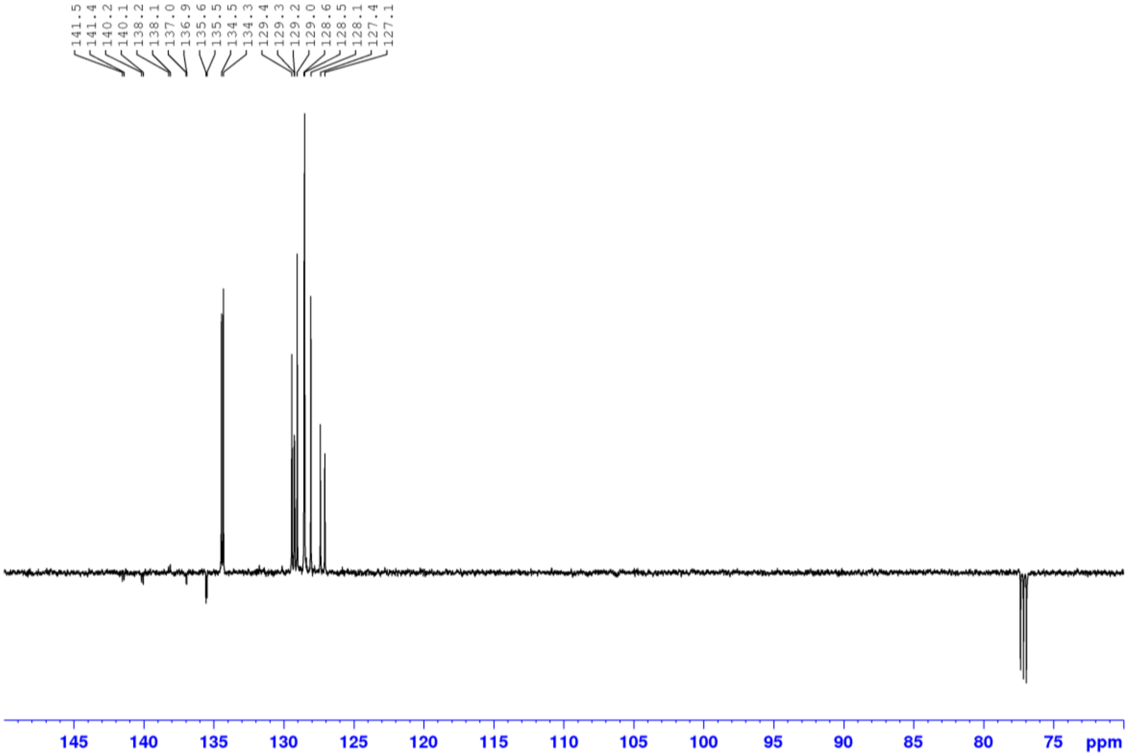


**Figure S91:** $^{13}C$ JMOD NMR spectrum for isolated $Ph(PPh_2)C{=}C(D)Ph$, in $CDCl_3$

## Donor Screening Experiments

To a solution of [*i*$Bu_3AlHLi]_2$, **11**, (0.05 mmol), diphenylacetylene (0.6 mmol) and $HPPh_2$ (0.5 mmol) in $d_8$-toluene (0.5 mL) was added the desired amount of Lewis donor ligand, and the reaction mixture loaded into a sealed J Young's NMR tube. The reaction was heated to 110 °C and monitored regularly by $^1H$ and $^{31}P$ NMR spectroscopies.

## 10 mol% of $iBu_3AlHLi(PMDETA)$

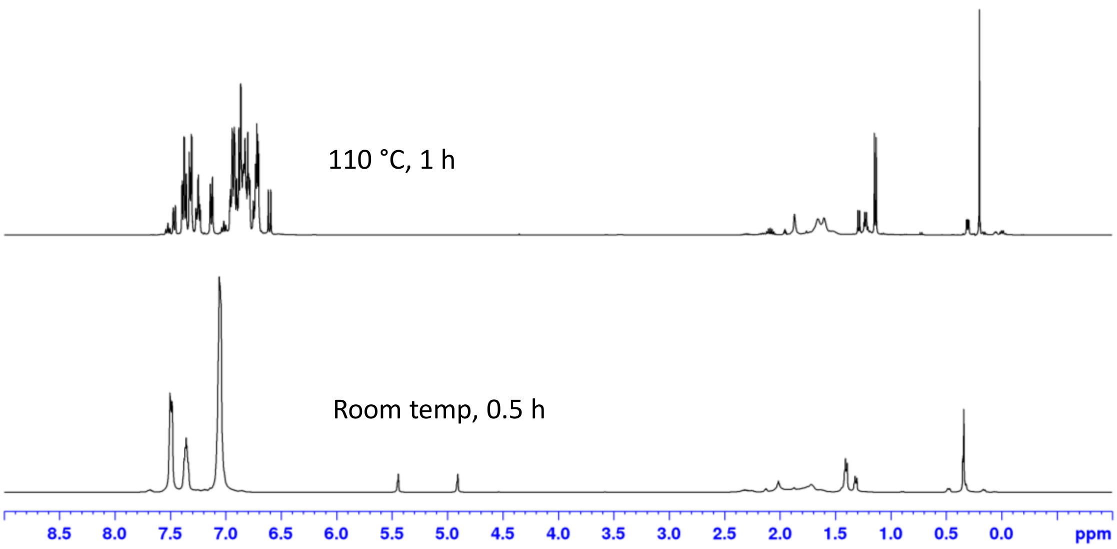


**Figure S92:** $^1H$ NMR spectrum for the hydrophosphination of diphenylacetylene catalysed by $iBu_3AlHLi(PMDETA)$ (10 mol%), in $d_8$-toluene.

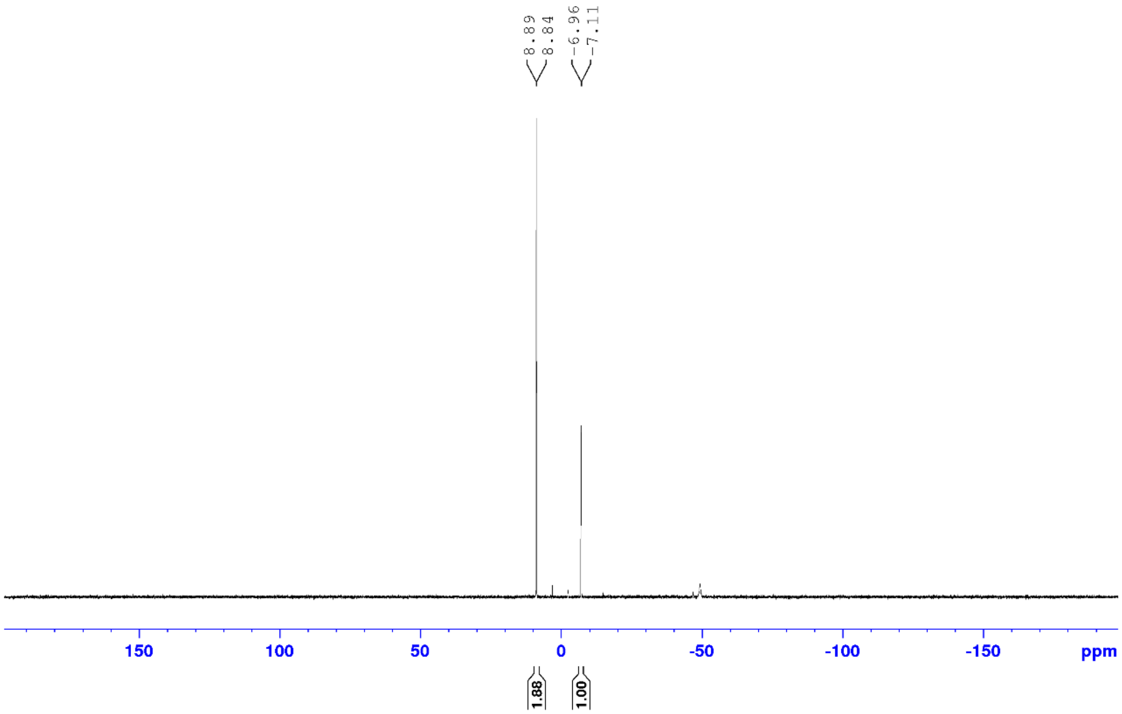


**Figure S93:** $^{31}P$ NMR spectrum for the hydrophosphination of diphenylacetylene catalysed by $iBu_3AlHLi(PMDETA)$ (10 mol%), in $d_8$-toluene

## 20 mol% TMEDA addition to **1**

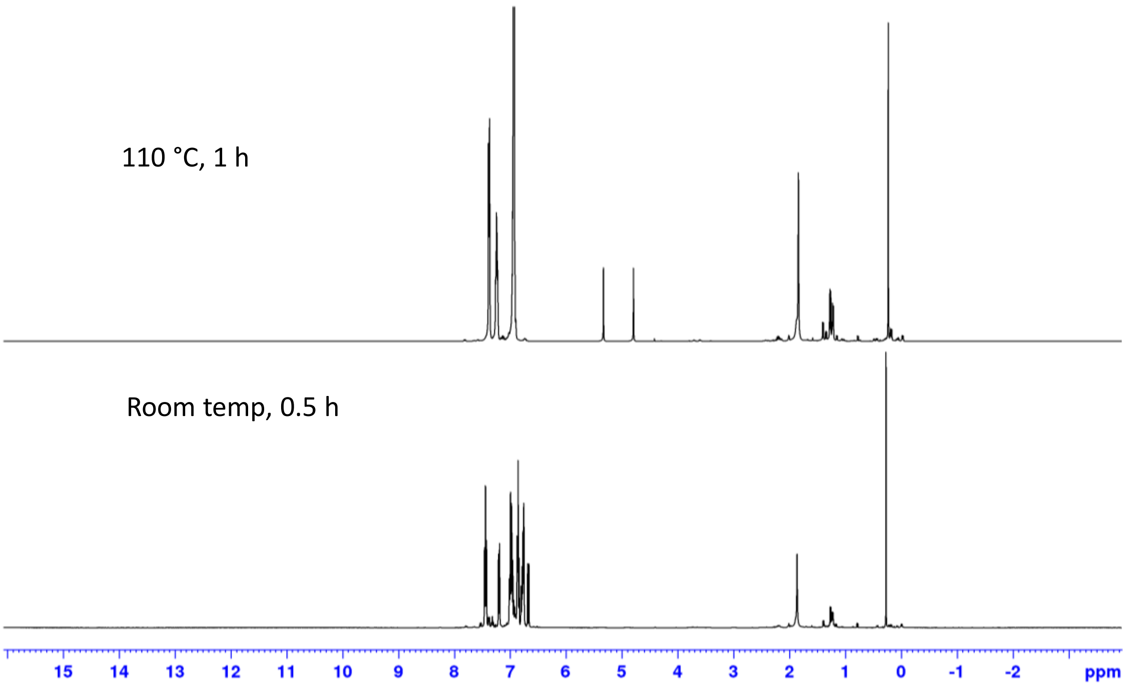


**Figure S94:** $^{1}$H NMR spectrum for the hydrophosphination of diphenylacetylene catalysed by **1** (10 mol%), in the presence of 20 mol% of TMEDA, in $d_8$-toluene

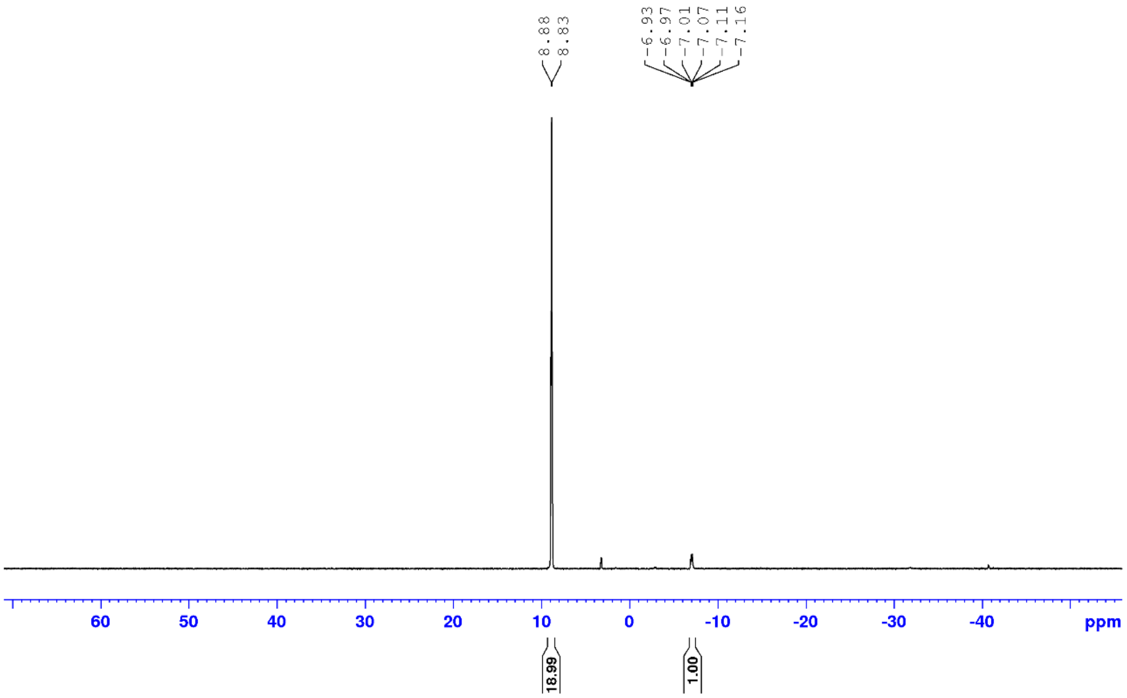


**Figure S95:** $^{31}P$ NMR spectrum for the hydrophosphination of diphenylacetylene catalysed by **1** (10 mol%), in the presence of 20 mol% of TMEDA, in $d_8$-toluene

## 10 mol% 12-crown-4 addition to **1**

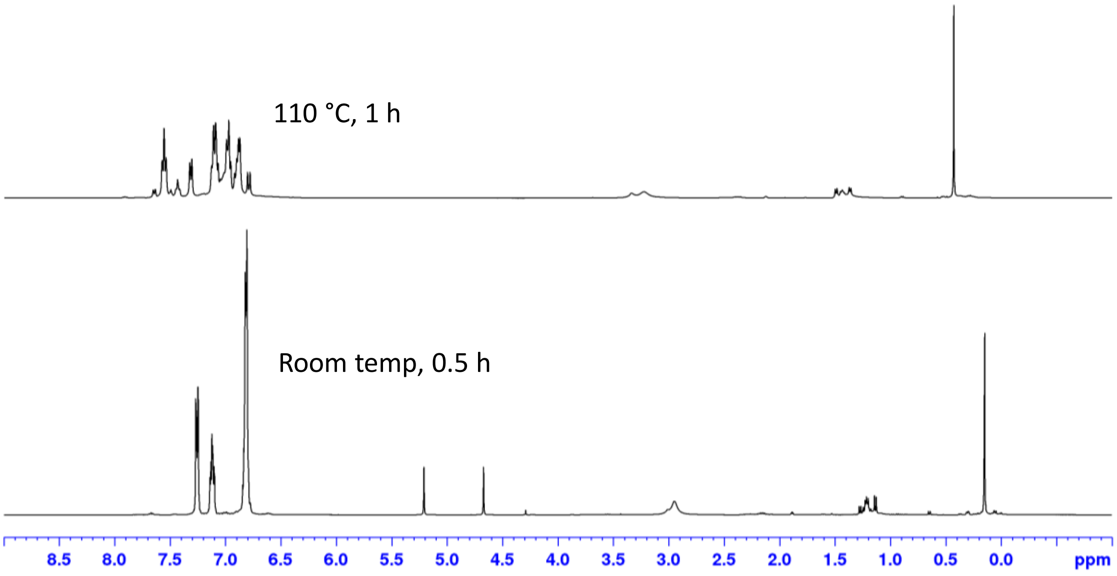


**Figure S96:** $^{1}H$ NMR spectrum for the hydrophosphination of diphenylacetylene catalysed by **1** (10 mol%), in the presence of 10 mol% of 12-crown-4, in $d_8$-toluene

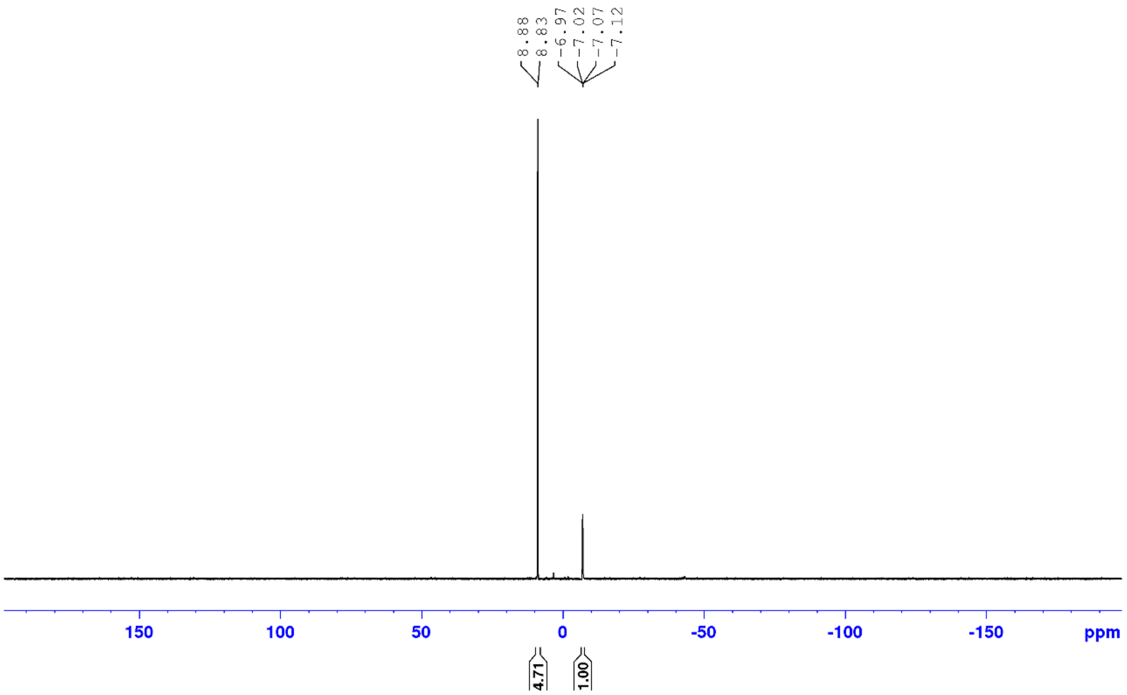


**Figure S97:** $^{31}P$ NMR spectrum for the hydrophosphination of diphenylacetylene catalysed by **1** (10 mol%), in the presence of 10 mol% of 12-crown-4, in $d_8$-toluene

## 10 mol% $Me_6$-TREN addition to **1**

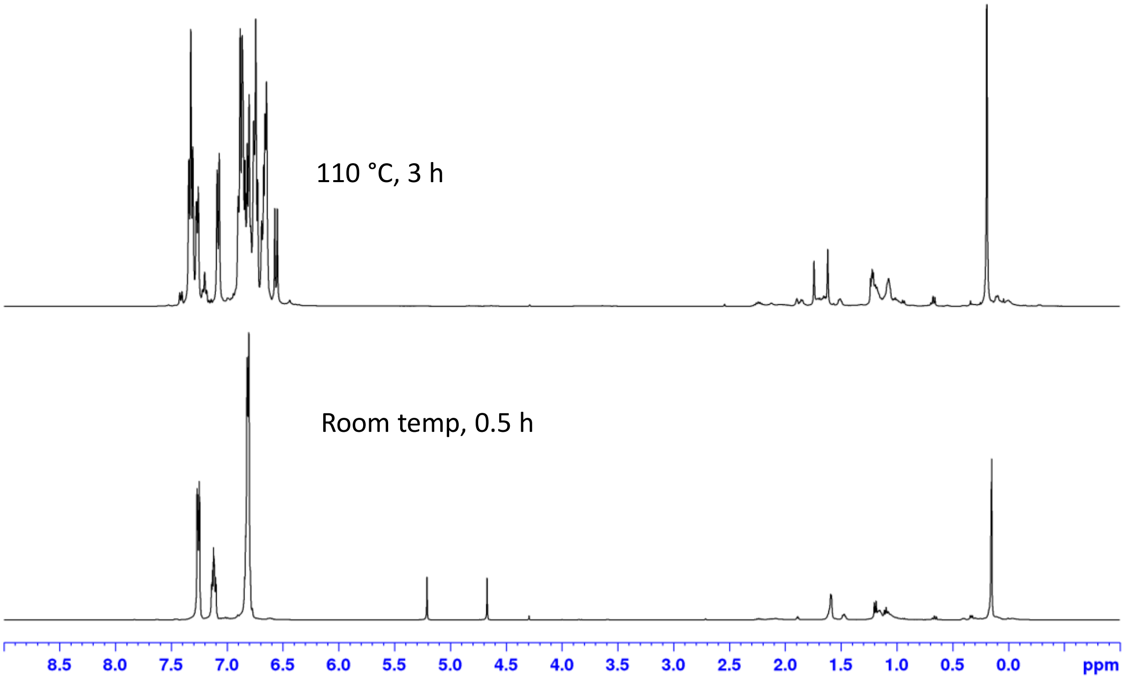


**Figure S98:** $^{1}H$ NMR spectrum for the hydrophosphination of diphenylacetylene catalysed by **1** (10 mol%), in the presence of 10 mol% of $Me_6$-TREN, in $d_8$-toluene

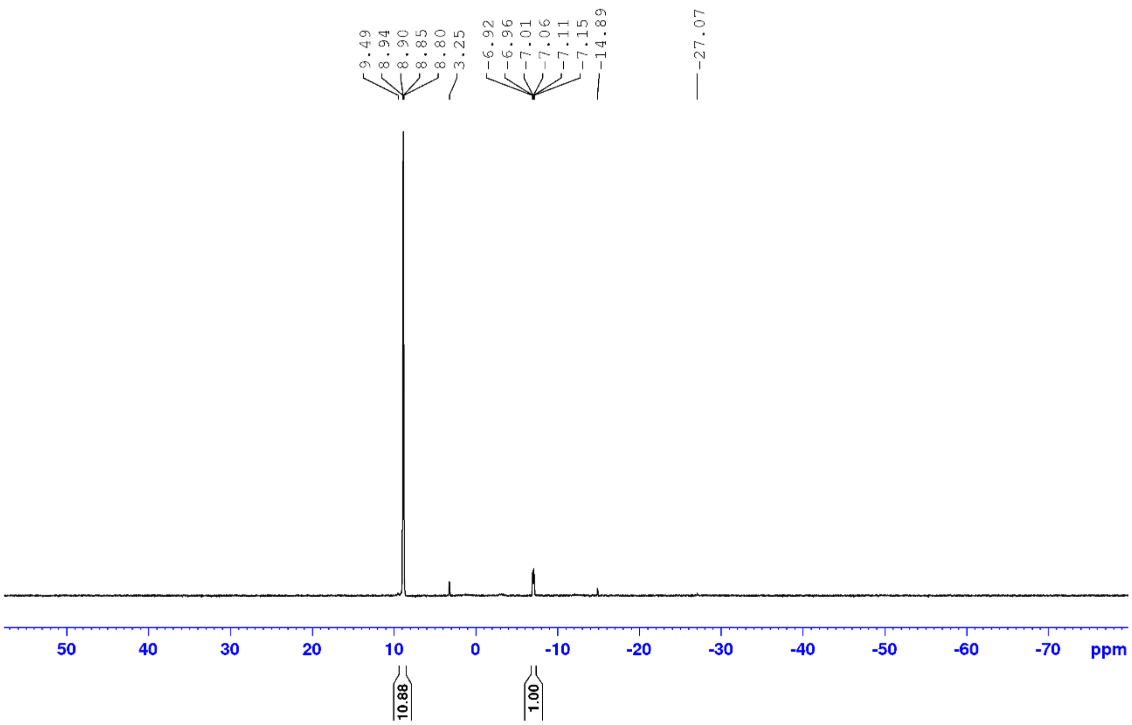


**Figure S99:** $^{31}$P NMR spectrum for the hydrophosphination of diphenylacetylene catalysed by **1** (10 mol%), in the presence of 10 mol% of $Me_6$-TREN, in $d_8$-toluene

## 20 mol% dppe addition to **1**

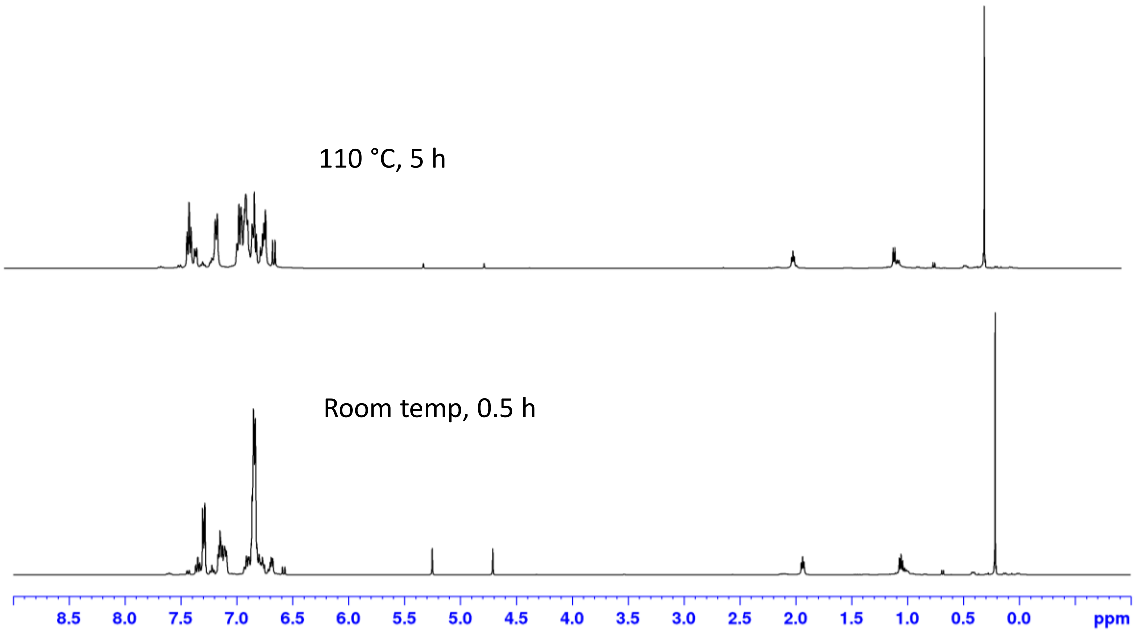


**Figure S100:** $^{1}$H NMR spectrum for the hydrophosphination of diphenylacetylene catalysed by **1** (10 mol%), in the presence of 20 mol% of dppe, in $d_8$-toluene

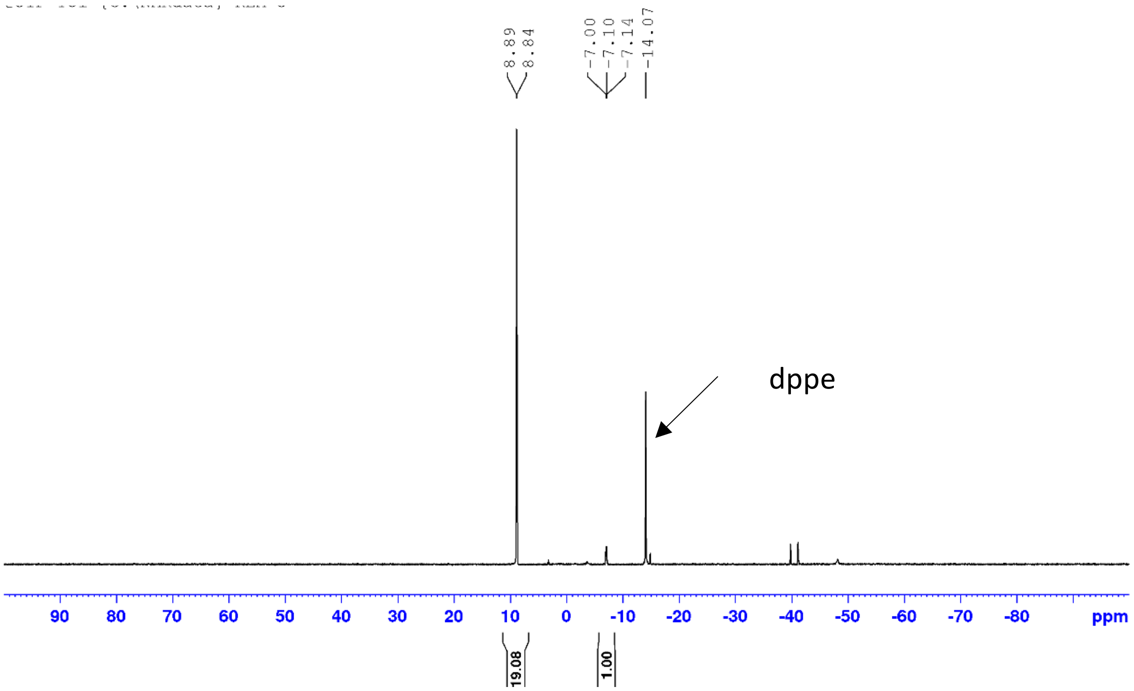


**Figure S101:** $^{31}P$ NMR spectrum for the hydrophosphination of diphenylacetylene catalysed by **1** (10 mol%), in the presence of 20 mol% of dppe, in $d_8$-toluene

## 30 mol% $PPh_3$ addition to **2**

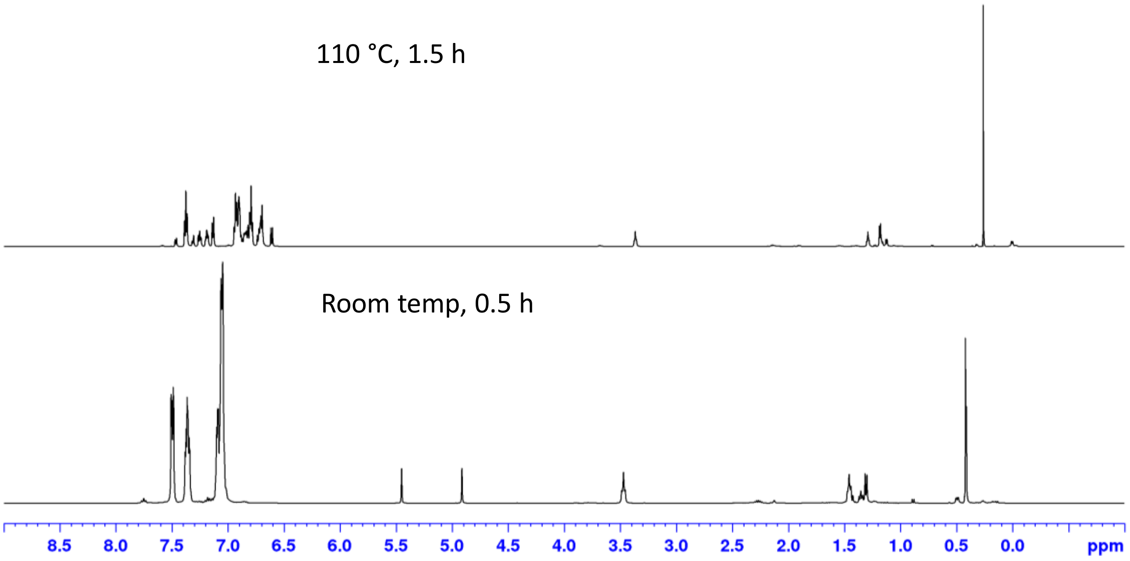


**Figure S102:** $^{1}H$ NMR spectrum for the hydrophosphination of diphenylacetylene catalysed by **1** (10 mol%), in the presence of 30 mol% of $PPh_3$, in $d_8$-toluene

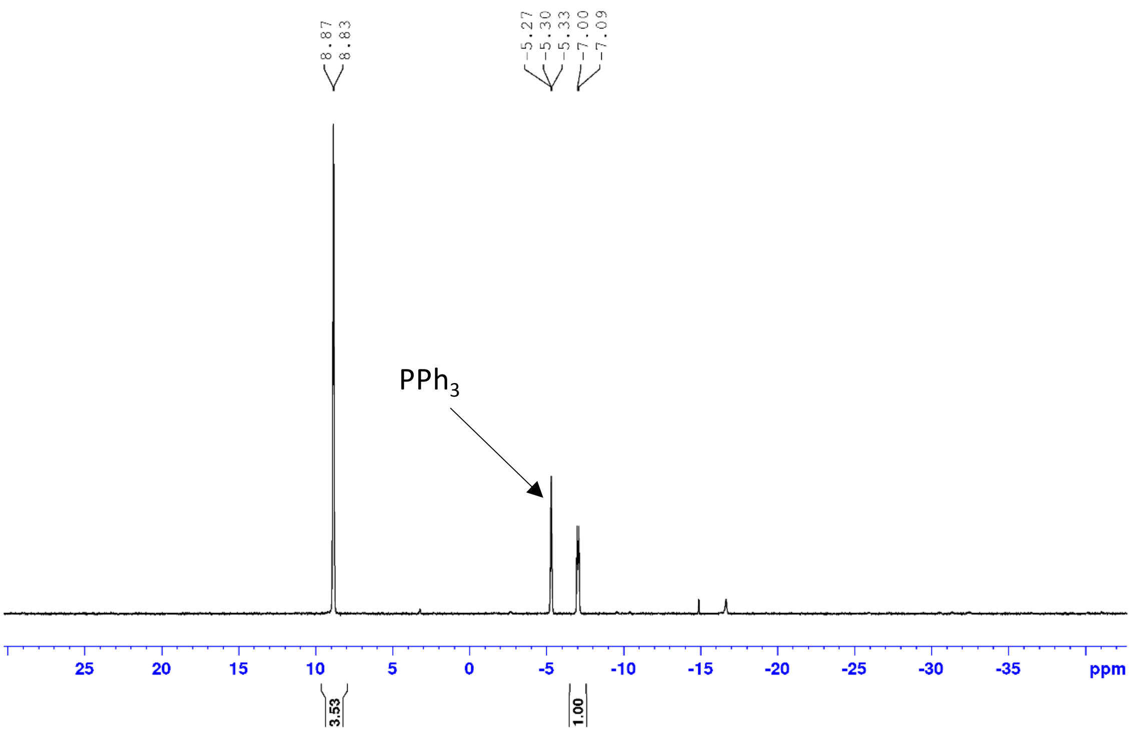


**Figure S103:** $^{31}P$ NMR spectrum for the hydrophosphination of diphenylacetylene catalysed by **1** (10 mol%), in the presence of 30 mol% of $PPh_3$, in $d_8$-toluene

## Kinetic Isotope Effect (KIE) experiment

$iBu_3AlPPh_2Li(THF)_3$ (10 mol%, 0.05 mmol, 0.0302 g) was added to 0.5 mL of $d_8$-toluene **or** $d_0$-toluene solution containing diphenyl acetylene (0.6 mmol; 0.1069 g) and $HPPh_2$ **or** $DPPh_2$ (0.5 mmol, 0.09 mL), as appropriate. This mixture was transferred to a sealed J. Young's tap NMR tube, heated to 100 °C, and monitored by $^{31}P$ NMR spectroscopy every 120 s. Spectra were Fourier transformed, phased, and base line corrected using Bruker Topspin software (version 3.57). The reaction rates were determined by monitoring the consumption of diphenyl phosphine and the formation of the vinyl phosphine product(s) over more than three half-lives. Reaction rate constants were derived from the plot of ln[diphenyl phosphine] vs time by using linear trend lines generated by Microsoft Excel software.

Rates for $HPPh_2$ and $DPPh_2$ used for the calculation of KIE are the average of two runs.

$HPPh_2$: average reaction rate (7.2 ± 0.4) x$10^{-4}$ $s^{-1}$

$DPPh_2$: average reaction rate (5.2 ± 0.4) x$10^{-4}$ $s^{-1}$

$$\mathrm{KIE} = \frac{k_H}{k_D} = \frac{7.2\mathrm{x}10^{-4}}{5.2\mathrm{x}10^{-4}} = 1.38 \pm 0.13$$

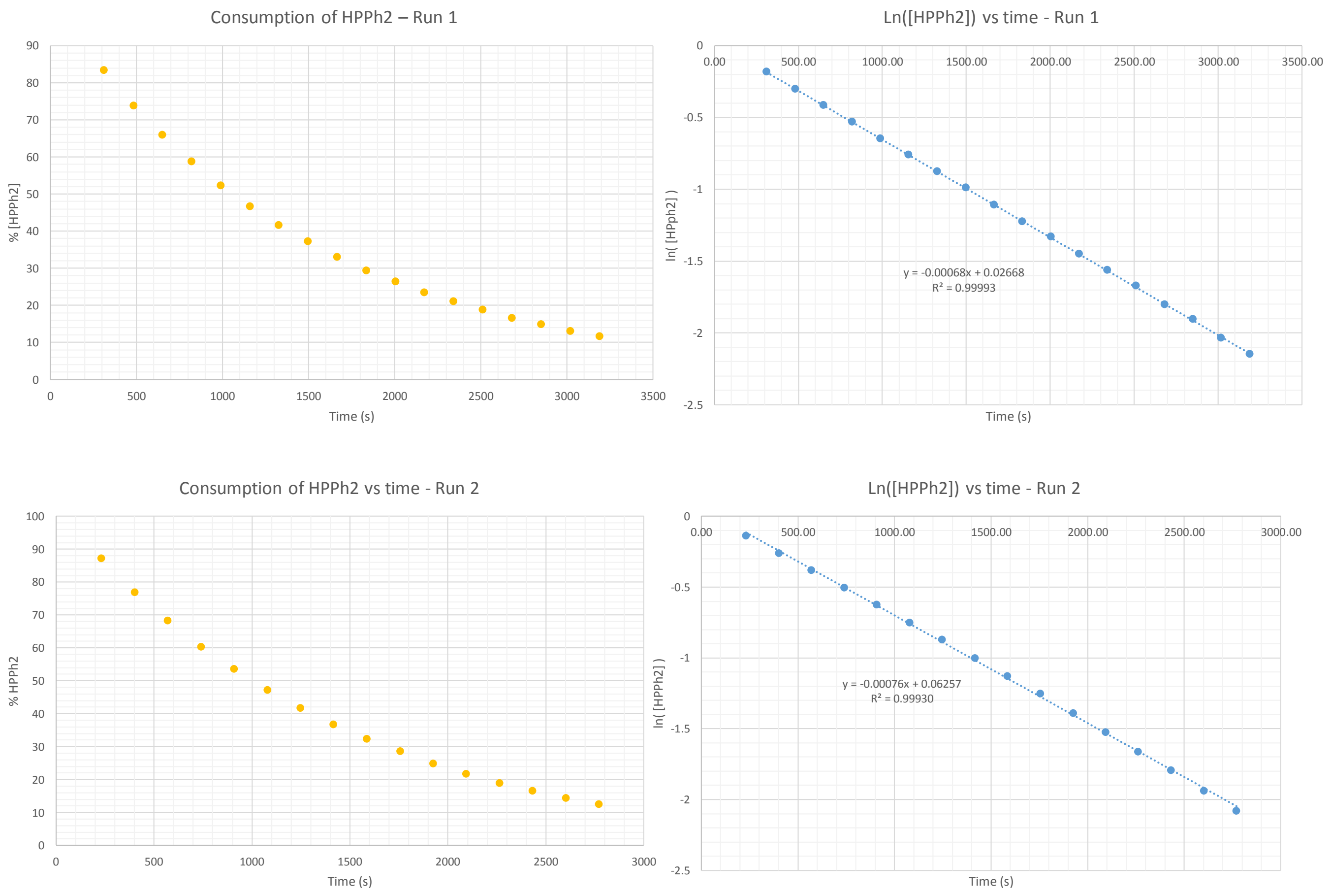


**Figure S104:** Kinetic data for the hydrophosphination of diphenyl acetylene using $HPPh_2$ at 100 ̊C for run 1 and run 2

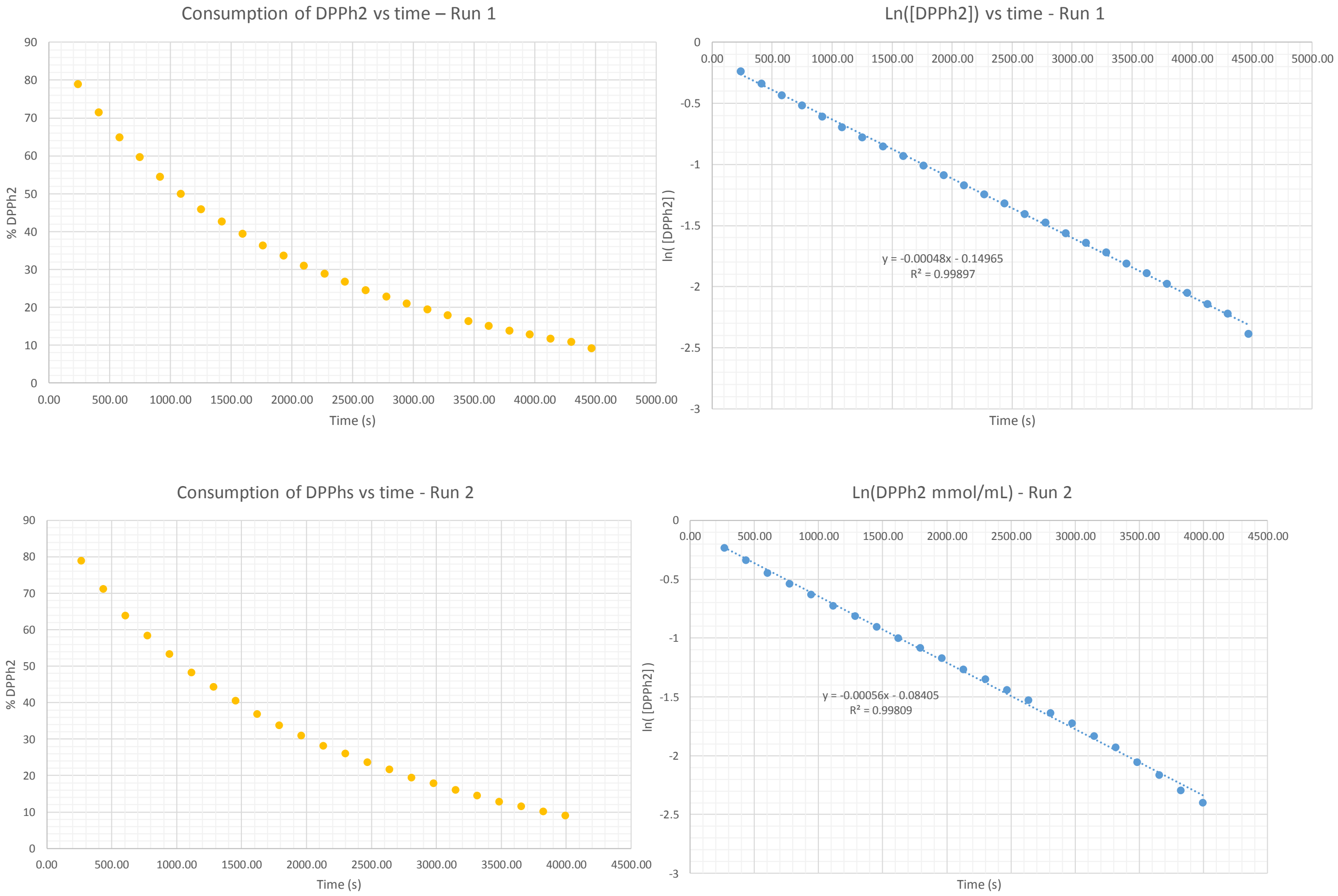


**Figure S105:** Kinetic data for the hydrophosphination of diphenyl acetylene using $DPPh_2$ at 100 °C for run 1 and run 2

## Kinetics

A reaction mixture of the desired concentrations of diphenylacetlyene, diphenyl phosphine and catalyst (**22**), with a total volume of 0.5 mL in $d_8$-toluene were transferred to a sealed J. Young's tap NMR tube, heated to 373 K, and monitored by $^{31}P$ NMR spectroscopy every 120 seconds and the consumption of diphenyl phosphine monitored over three half-lives. Spectra were Fourier transformed, phased, and base line corrected using Bruker Topspin software (version 3.57). Orders in reagents were examined using the Burés method with Microsoft Excel software.[8]

| [$HPPh_2$] (M) | [alkyne] (M) | [22] (M) | $r$ x $10^4$ (mol $s^{-1}$) |
|---|---|---|---|
| 1.1 | 1.2 | 0.1 | 5.34 |
| 1.0 | 1.2 | 0.1 | 9.19 |
| 0.9 | 1.2 | 0.1 | 10.23 |
| 0.8 | 1.2 | 0.1 | 14.70 |
| 0.7 | 1.2 | 0.1 | 15.21 |

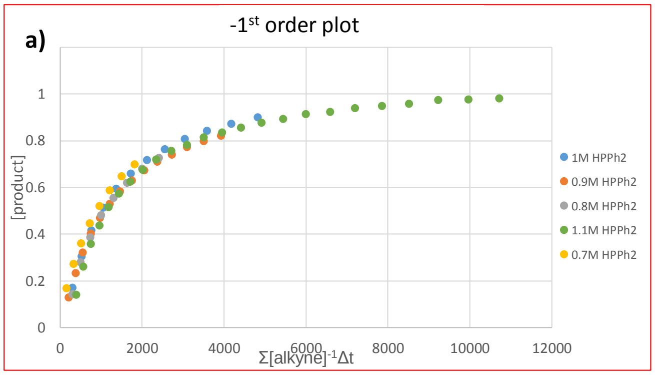


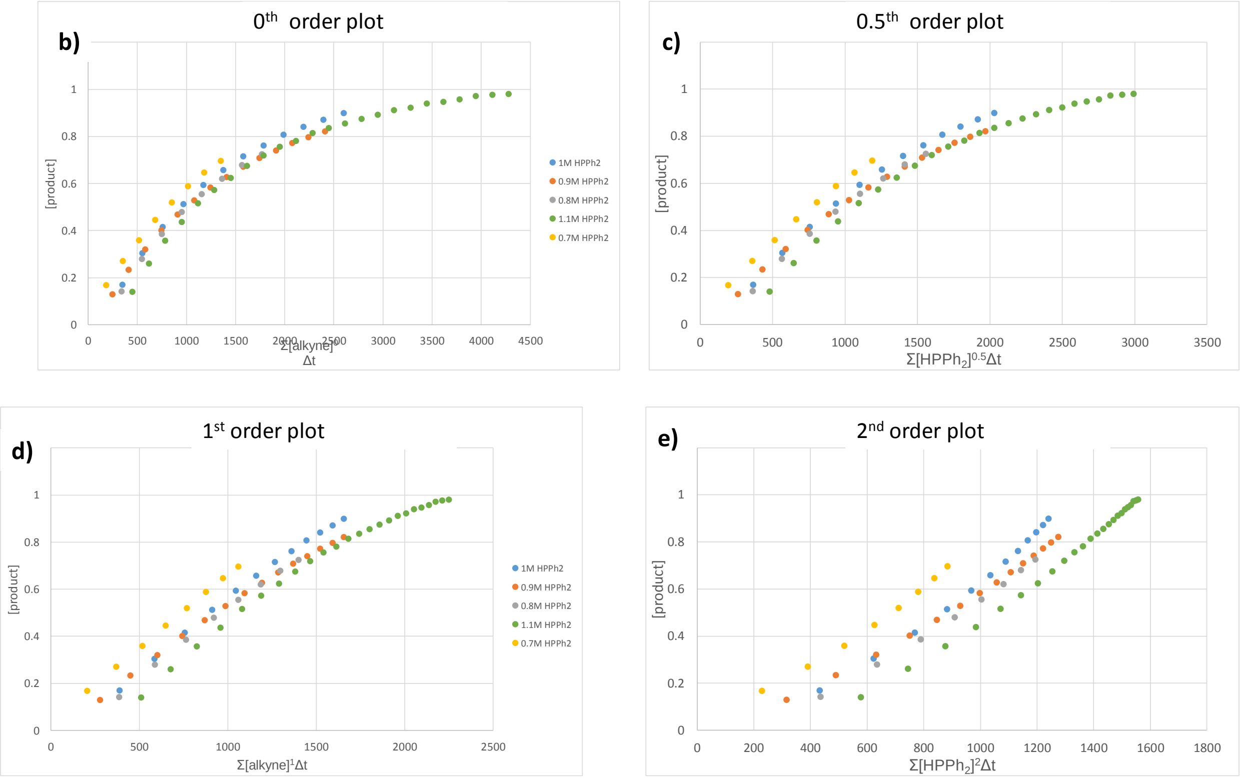


**Figure S106:** Effect of changing phosphine concentration on reaction rate. Variable Time Normalisation Analysis visual kinetic analysis plots for reaction rate dependence on order in $HPPh_2$ showing plot for **a)** -1$^{st}$ order; **b)** 0$^{th}$ order; **c)** 0.5$^{th}$ order; **d)** 1$^{st}$ order, and **e)** 2$^{nd}$ order plots.

| [HPPh$_2$] (M) | [alkyne] (M) | [22] (M) | *r* x 10$^4$ (mol s$^{-1}$) |
|---|---|---|---|
| 1.0 | 1.0 | 0.1 | 4.92 |
| 1.0 | 1.2 | 0.1 | 9.20 |
| 1.0 | 1.3 | 0.1 | 11.47 |
| 1.0 | 1.4 | 0.1 | 14.05 |

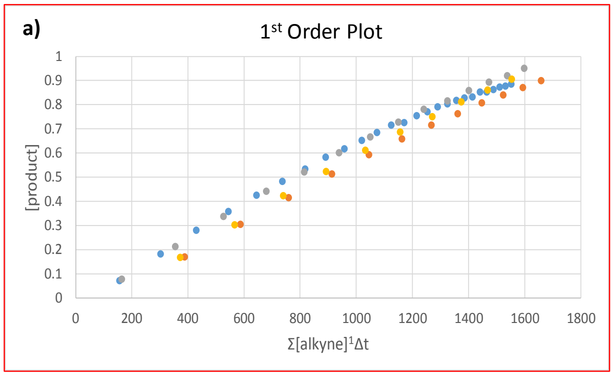


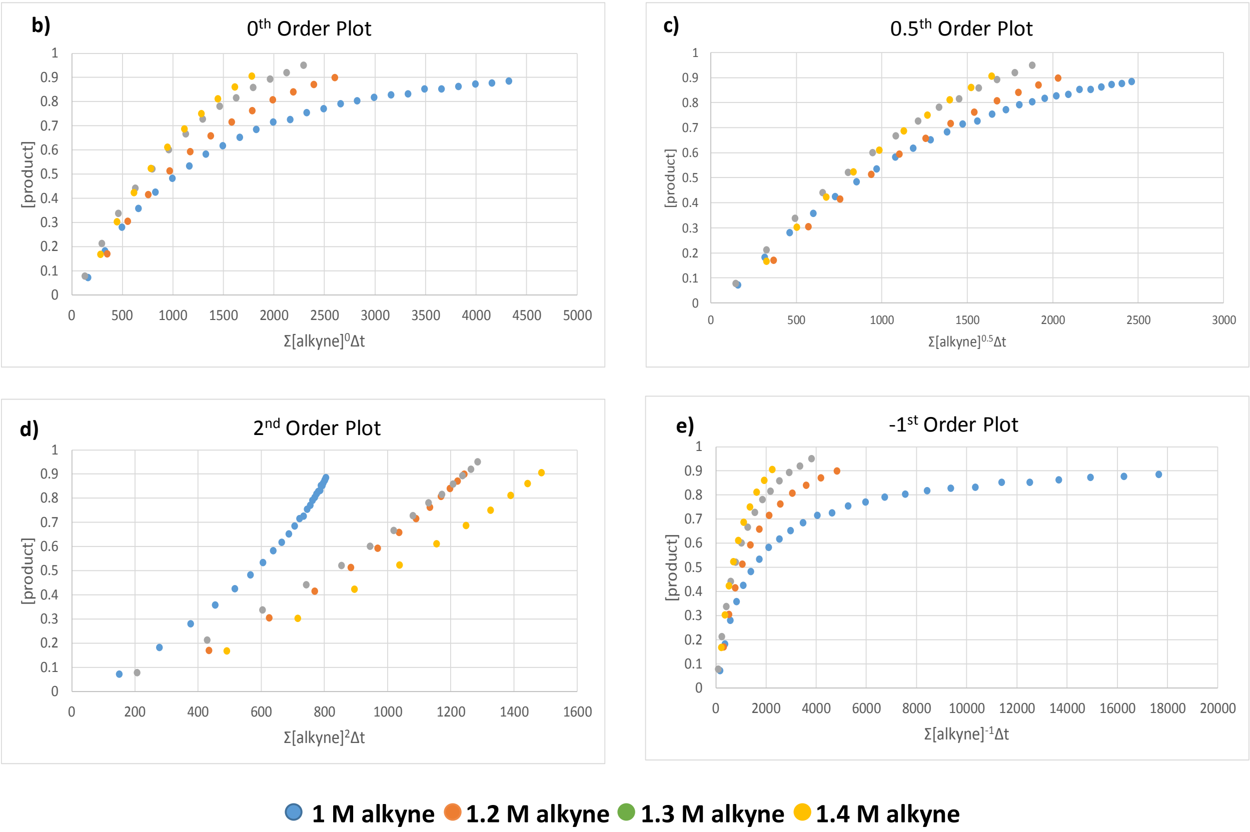


**Figure S107:** Effect of changing diphenylacetylene concentration on reaction rate. Variable Time Normalisation Analysis visual kinetic analysis plots for reaction rate dependence on order in HPPh$_2$ showing plot for **a)** 1$^{st}$ order; **b)** 0$^{th}$ order; **c)** 0.5$^{th}$ order; **d)** 2$^{nd}$ order, and **e)** -1$^{st}$ order plots.

| [$HPPh_2$] (M) | [alkyne] (M) | [22] (M) | $r \times 10^4$ (mol $s^{-1}$) |
|---|---|---|---|
| 1.0 | 1.2 | 0.05 | 3.542 |
| 1.0 | 1.2 | 0.075 | 6.035 |
| 1.0 | 1.2 | 0.1 | 9.19 |
| 1.0 | 1.2 | 0.125 | 12.02 |

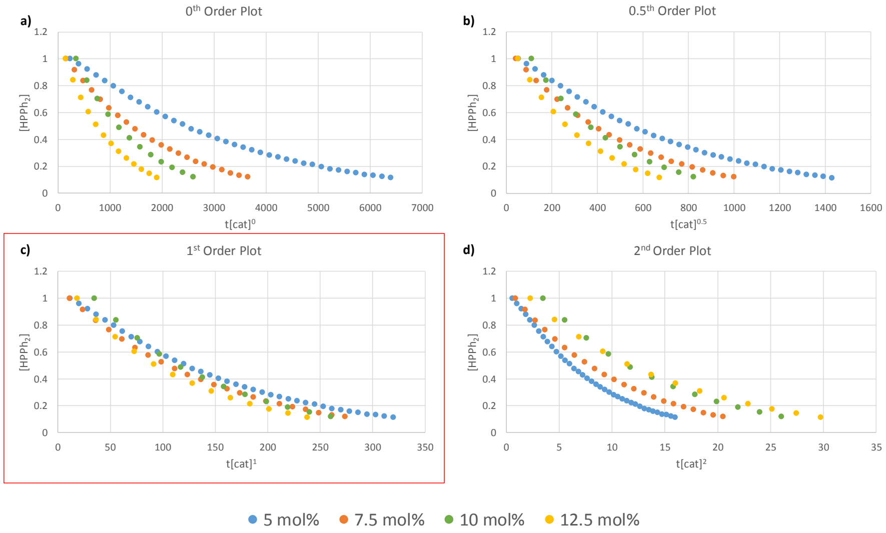


**Figure S108:** Effect of changing catalyst loading (**22**) on reaction rate. Variable Time Normalisation Analysis visual kinetic analysis plots for reaction rate dependence on order in $HPPh_2$ showing plot for **a)** $0^{th}$ order; **b)** $0.5^{th}$ order; **c)** $1^{st}$ order, and **d)** $2^{nd}$ order plots.

## DFT Calculations

Run on the full system with the internal alkyne, diphenylacetylene, used as the model substrate, the calculations were performed at the B3LYP-D3/[9] 6-311G(d,p)[10] level of theory employing a continuum solvent with the dielectric constant of toluene within the IEFPCM model.[11]

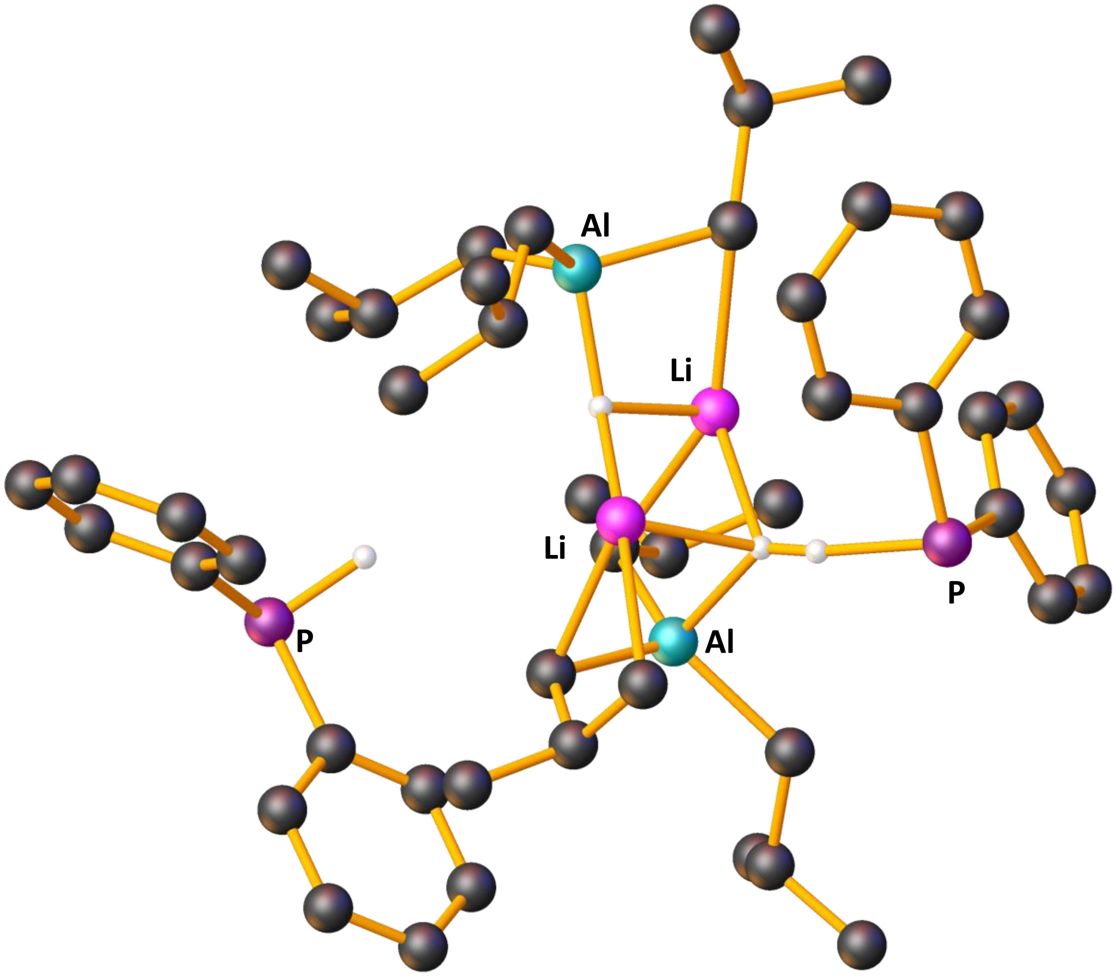


**Figure S109:** Molecular structure for the highpoint from the bond scan used to calculate the indicative barrier in the deprotonation of $HPPh_2$ by **1**. Hydrogen atoms except for P – H and hydrides have been omitted for clarity.

## Control reactions

Preformed under experimental conditions (0.6 mmol substrate, 0.5 mmol $HPPh_2$, 0.5 mL $d_8$-toluene, 110 °C), in the absence of any catalyst.

## Phenylacetylene

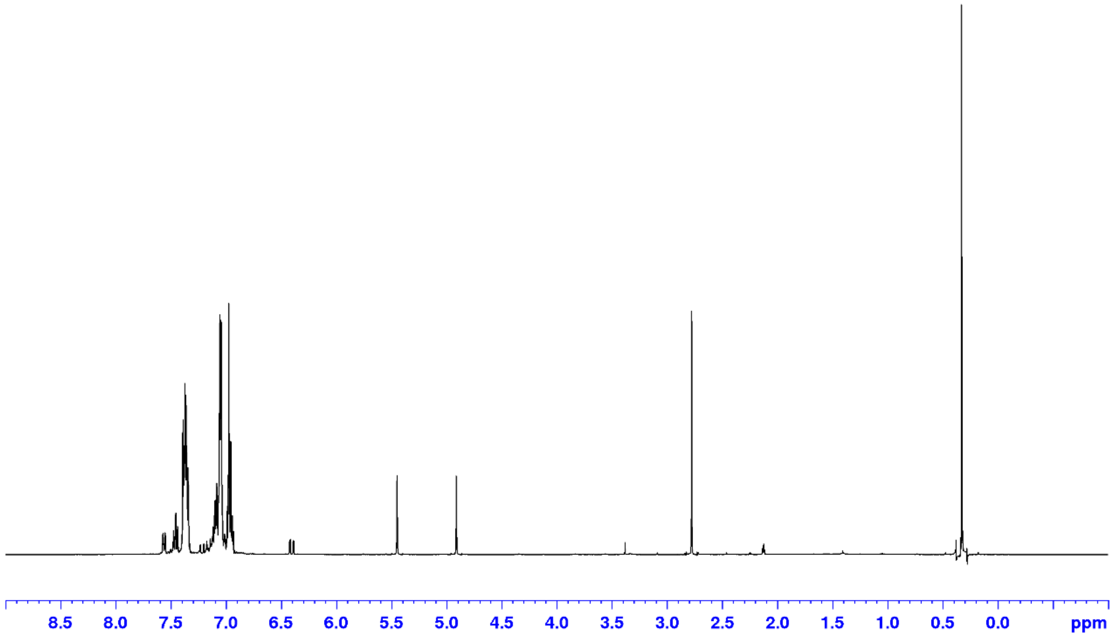


**Figure S110:** $^1H$ NMR spectrum for the hydrophosphination of phenylacetylene after 20 h at 110 °C in the absence of catalyst, in $d_8$-toluene.

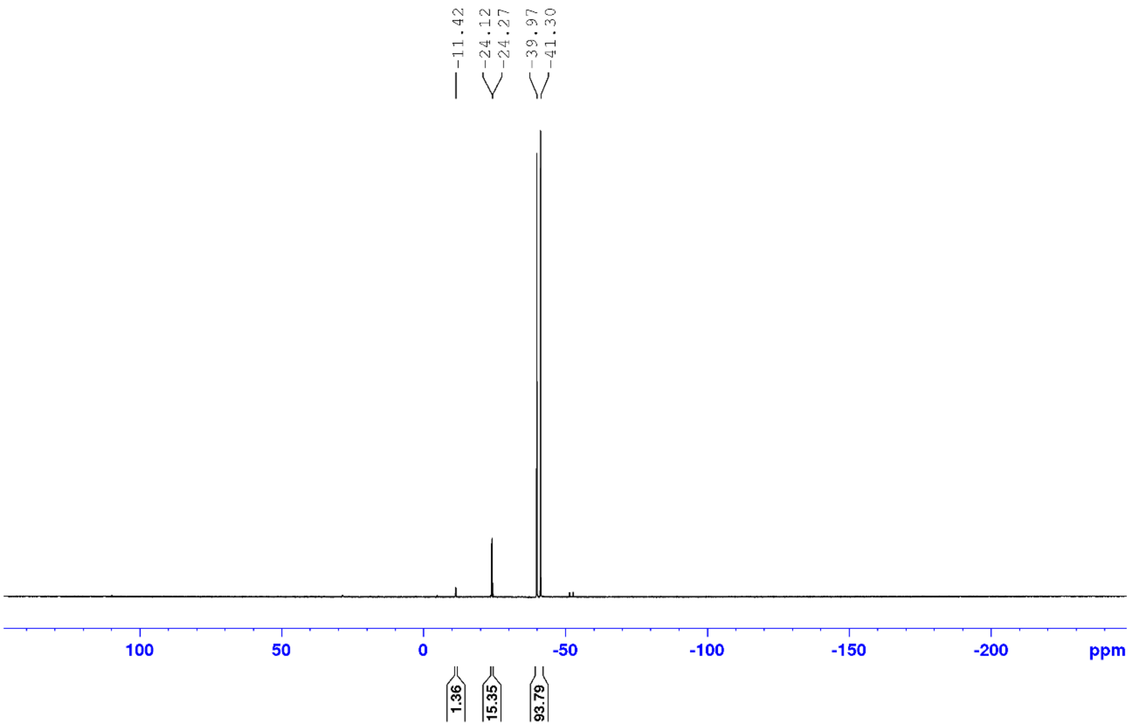


**Figure S111:** $^{31}$P NMR spectrum for the hydrophosphination of phenylacetylene after 20 h at 110 °C in the absence of catalyst, in $d_8$-toluene.

## Diphenylacetylene

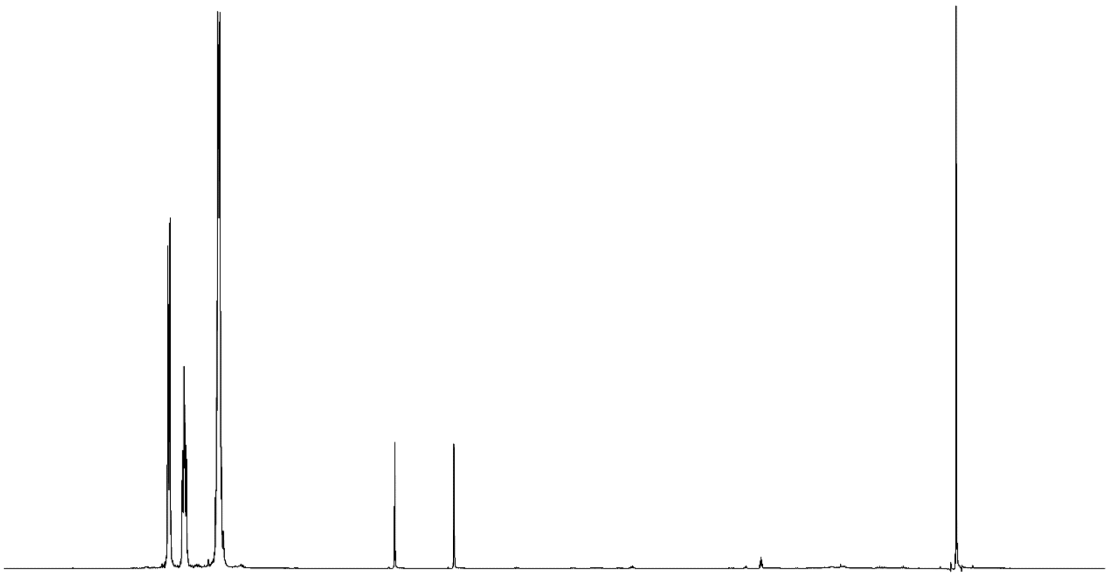

**Figure S112:** $^{1}$H NMR spectrum for the hydrophosphination of diphenylacetylene after 5 h at 110 °C in the absence of catalyst, in $d_8$-toluene.

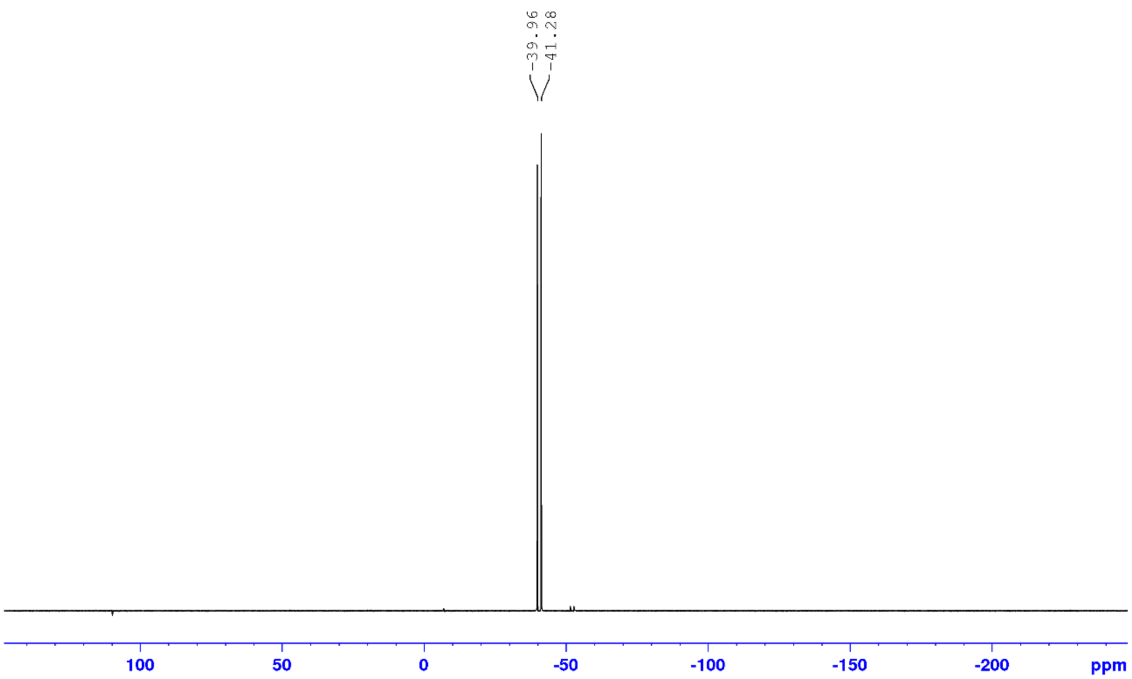


**Figure S113:** $^{31}P$ NMR spectrum for the hydrophosphination of diphenylacetylene after 5 h at 110 °C in the absence of catalyst, in $d_8$-toluene.

## Styrene

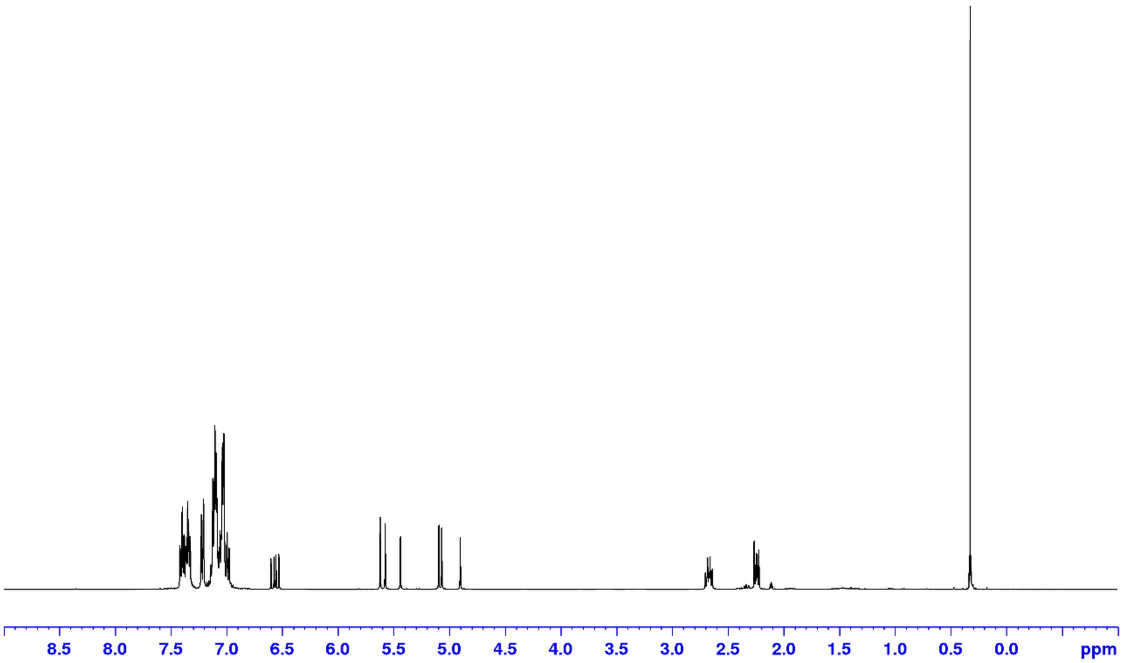


**Figure S114:** $^{1}H$ NMR spectrum for the hydrophosphination of styrene after 6 h at 110 °C in the absence of catalyst, in $d_8$-toluene.

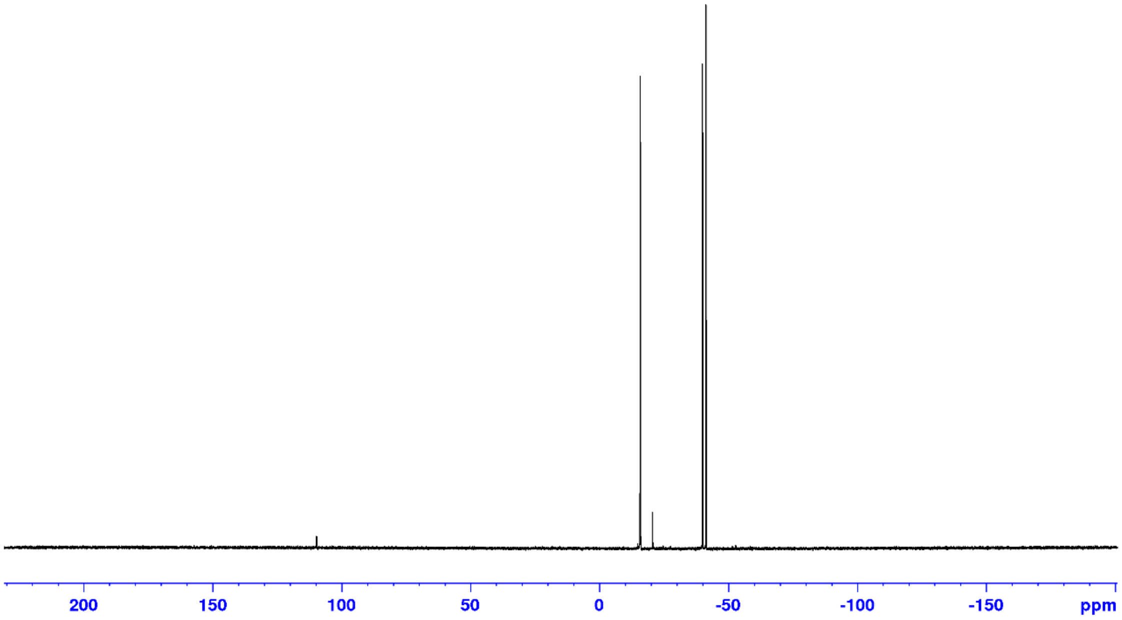


**Figure S115:** $^{31}P$ NMR spectrum for the hydrophosphination of styrene after 5 h at 110 °C in the absence of catalyst, in $d_8$-toluene.

## Diisopropylcarbodiimide

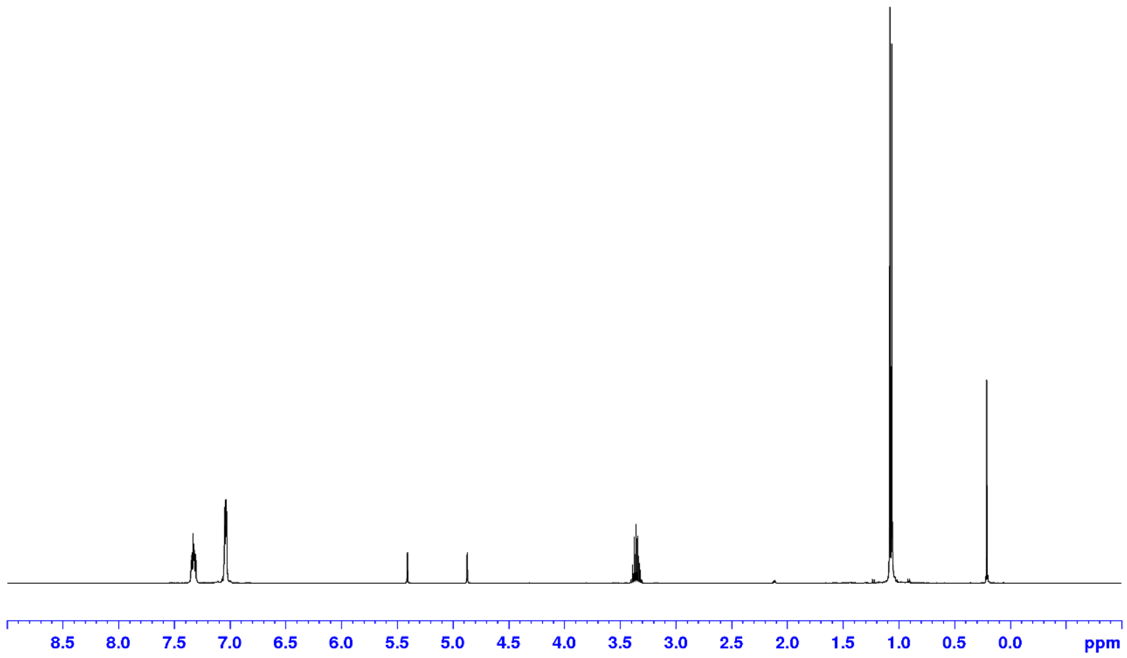


**Figure S116:** $^{1}H$ NMR spectrum for the hydrophosphination of diisopropylcarbodiimide after 22 h at 50 °C in the absence of catalyst, in $d_8$-toluene. Note that the catalytic reactions preformed here are at room temperature.

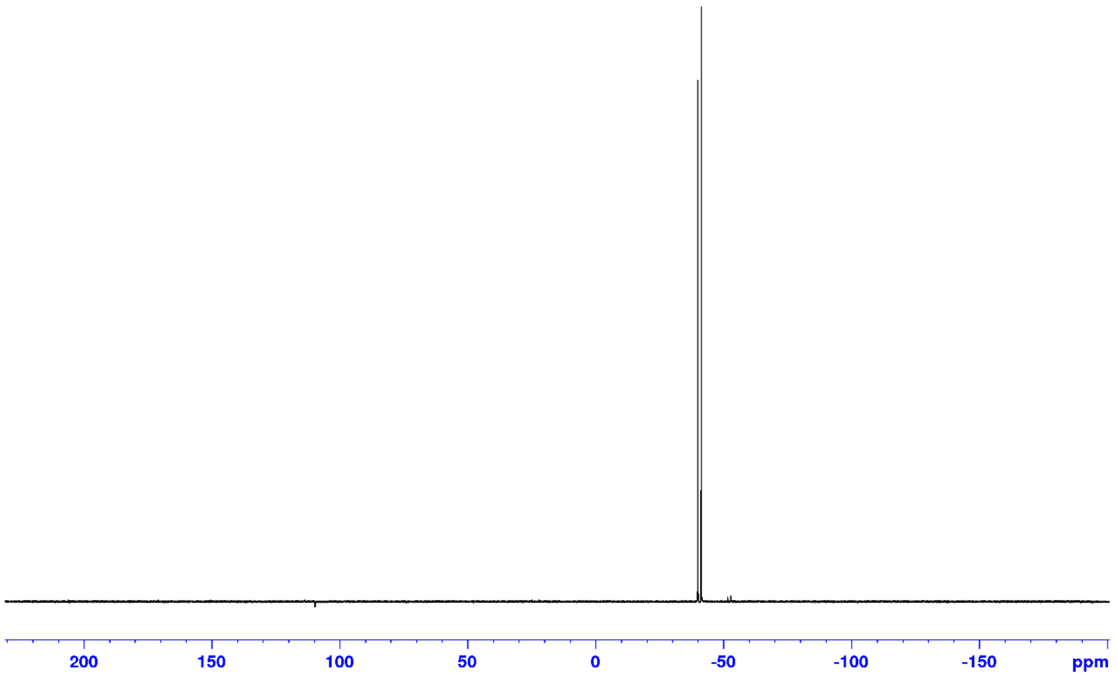


**Figure S117:** $^{31}P$ NMR spectrum for the hydrophosphination of diisopropylcarbodiimide after 22 h at 50 °C in the absence of catalyst, in $d_8$-toluene. Note that the catalytic reactions preformed here are at room temperature.

[1] V. A. Pollard, M. A. Fuentes, A. R. Kennedy, R. McLellan, R. E. Mulvey, *Angew. Chem. Int. Ed.* **2018**, *57*, 10651-10655.
[2] S. A. Sangokoya, W. T. Pennington, G. H. Robinson, D. C. Hrncir, *J. Organomet. Chem.* **1990**, *385*, 23-31.
[3] A. Schmidt, A. R. Nödling, G. Hilt, *Angew. Chem. Int. Ed.* **2015**, *54*, 801-804.
[4] R. Neufeld, D. Stalke, *Chem. Sci.* **2015**, *6*, 3354-3364.
[5] a) G. M. Sheldrick, *Acta Crystallogr.* **2008**, *A64*, 112 - 122; b) G. M. Sheldrick, *Acta Crystallogr.* **2015**, *C71*, 3 -8
[6] A. P. Dove, V. C. Gibson, E. L. Hormnirun, E. L. Marshall, J. A. Segal, A. J. P. White, D. J. Williams, *Dalton Trans.* **2003**, 3088 - 3097.
[7] O. V. Dolomanov, L. J. Bourhis, R. J. Gildea, J. A. K. Howard, H. Puschmann, *J. Appl. Crystallogr.* **2009**, *42*, 339 - 341.
[8] a) J. Burés, *Angew. Chem. Int. Ed.* **2016**, *55*, 2028-2031; b) J. Burés, *Angew. Chem. Int. Ed.* **2016**, *55*, 16084-16087; c) C. D. T. Nielsen, J. Burés, *Chem. Sci.* **2019**, *10*, 348-353.
[9] a) A. D. Becke, *Phys. Rev. A* **1988**, *38*, 3098-3100; b) A. D. Becke, *J. Chem. Phys.* **1993**, *98*, 5648-5652; c) C. Lee, W. Yang, R. G. Parr, *Phys. Rev. B* **1988**, *37*, 785-789; d) S. H. Vosko, L. Wilk, M. Nusair, *Can. J. Phys.* **1980**, *58*, 1200-1211; e) P. J. Stephens, F. J. Devlin, C. F. Chabalowski, M. J. Frisch, *J. Phys. Chem.* **1994**, *98*, 11623-11627; f) R. H. Hertwig, W. Koch, *Chem. Phys. Lett.* **1997**, *268*, 345-351; g) S. Grimme, J. Antony, S. Ehrlich, H. Krieg, *J. Chem. Phys.* **2010**, *132*, 154104.
[10] a) A. D. McLean, G. S. Chandler, *J. Chem. Phys.* **1980**, *72*, 5639-5648; b) R. Krishnan, J. S. Binkley, R. Seeger, J. A. Pople, *J. Chem. Phys.* **1980**, *72*, 650-654; c) M. J. Frisch, J. A. Pople, J. S. Binkley, *J. Chem. Phys.* **1984**, *80*, 3265-3269.
[11] J. Tomasi, B. Mennucci, R. Cammi, *Chem. Rev.* **2005**, *105*, 2999-3094.
